# A systematic review of artificial intelligence in small ruminant production systems: applications, performance outcomes, and reported implementation challenges

**DOI:** 10.1186/s12917-026-05806-z

**Published:** 2026-08-20

**Authors:** Abdullah Ali Ghazy, Ibrahim Atta Abu El-Naser

**Affiliations:** 1 Department of Animal Production, Faculty of Agriculture, Suez Canal University, Ismailia, 41522 Egypt; 2 Department of Animal, Poultry and Fish Production, Faculty of Agriculture, Damietta University, Damietta, Egypt

**Keywords:** Artificial Intelligence, Machine Learning, Small Ruminants, Sheep, Goats, Precision Livestock Farming, Automated Monitoring, Systematic Review

## Abstract

**Background:**

Sheep and goats are essential components of global livestock systems, supporting smallholder livelihoods and contributing substantially to meat, milk, and fiber production. However, many small ruminant production systems remain extensive and constrained by limited infrastructure, labor availability, and challenges in continuous animal monitoring. Artificial Intelligence (AI) offers new opportunities for precision livestock management through automated data analysis, early detection of production- and health-related changes, and improved decision support. This systematic review aimed to characterize current AI applications in sheep and goat production, summarize reported performance outcomes across all major application domains, and identify barriers affecting practical implementation.

**Results:**

A systematic search of Web of Science, Scopus, and PubMed identified peer-reviewed studies published between January 2020 and December 2025. From 11,035 records screened, 92 studies met the inclusion criteria and were synthesized narratively; meta-analysis was not conducted due to substantial methodological heterogeneity across studies. AI applications spanned six domains: behavior and activity recognition (26.1%, *n *= 24; mean accuracy 92.4%, range 66.7–100%), individual animal identification (19.6%, *n* = 18; mean accuracy 97.3%, range 93.3–99.9%), health, welfare, and disease detection (19.6%, *n* = 18; mean accuracy 89.7%, range 62.0–99.0%), growth and body measurement (9.8%, *n* = 9; mean R^2^ = 0.86), genomics and molecular biology (8.7%, *n* = 8; mean accuracy 97.8%), and production, technical, and environmental applications (16.3%, *n* = 15; mean accuracy 93.1%). Convolutional Neural Networks, YOLO-based models, and Random Forest algorithms were the most frequently applied approaches. Publications grew markedly over the review period, with 28.3% published in 2025 alone. Fewer than half of included studies used fully independent external validation. Key implementation barriers included limited dataset diversity, class imbalance, environmental complexity, and hardware constraints.

**Conclusions:**

Current evidence indicates that AI has strong potential to enhance small ruminant production, particularly for automated monitoring and biometric identification under controlled conditions. However, translation into sustainable real-world applications remains limited by methodological inconsistencies, restricted validation across farms and breeds, and insufficient representation of extensive production environments. Future progress will require standardized public datasets, transparent domain-appropriate metric reporting, rigorous independent validation, and deployment-focused evaluation under practical farming conditions.

**Supplementary Information:**

The online version contains supplementary material available at 10.1186/s12917-026-05806-z.

## Background

### Global context and structural constraints in small ruminant systems

Sheep (*Ovis aries*) and goats (*Capra hircus*) occupy a central position in global agricultural systems, sustaining the livelihoods of millions of smallholder farmers and contributing substantially to meat, milk, and fiber production worldwide [1]. Beyond their economic value, small ruminants play a critical role in food security, climate resilience, and the utilization of marginal lands unsuitable for intensive cropping [2]. Despite this global importance, technological innovation in sheep and goat production has historically lagged behind that of intensively managed species such as poultry, swine, and dairy cattle [3, 4].

This innovation gap is closely linked to the structural characteristics of small ruminant production systems. Flocks are frequently managed under extensive or semi-extensive conditions, characterized by dispersed grazing, variable environmental exposure, limited infrastructure, and significant labor constraints [5, 6]. These structural realities restrict continuous individual animal monitoring and delay timely health or reproductive interventions. As a result, production inefficiencies persist, including elevated neonatal mortality associated with undetected dystocia [7], substantial economic losses due to gastrointestinal parasitism [8, 9], and delayed detection of locomotion disorders such as lameness [10]. Collectively, these constraints highlight the urgent need for scalable, non-invasive, and automated monitoring solutions capable of functioning reliably within heterogeneous and resource-constrained production environments.

### AI and the emergence of precision small ruminant farming

The rapid evolution of Artificial Intelligence (AI), embedded within Precision Livestock Farming (PLF) systems, offers a paradigm shift from reactive herd management toward predictive, data-driven decision-making [4]. In this review, the term AI is used as an umbrella concept encompassing machine learning (ML), deep learning (DL), and closely related computational approaches. ML refers to algorithms that learn patterns from data without being explicitly programmed (e.g., Random Forest, SVM, Gradient Boosting). DL denotes a subclass of ML employing multi-layer neural architectures capable of automated feature extraction (e.g., CNNs, LSTMs, Transformers). Studies relying exclusively on rule-based expert systems or conventional statistical modeling without an adaptive learning component were excluded. By integrating wearable sensors, inertial measurement units (IMUs), computer vision systems, acoustic monitoring technologies, and advanced machine learning algorithms, AI enables automated behavior classification, reproductive event detection, health status assessment, and performance prediction at the individual animal level.

While AI adoption has accelerated markedly in dairy cattle and poultry systems [4], its application in sheep and goat production remains comparatively fragmented and unevenly distributed across research domains. An important distinction must be drawn between technical feasibility and operational readiness: technical feasibility (accuracy under controlled conditions) is necessary but insufficient for practical deployment, which additionally requires robustness under environmental variability, cross-breed generalizability, economic affordability, and end-user acceptance. The reviewed literature predominantly demonstrates technical feasibility; evidence of operational readiness under real-world farm conditions remains limited. Importantly, frameworks developed for intensively managed dairy systems cannot be directly transferred to extensive small ruminant systems without adaptation. Differences in management intensity, environmental variability, flock dynamics, and data availability necessitate species-specific methodological approaches and validation strategies [11, 12]. Consequently, although emerging studies demonstrate promising advances across multiple thematic areas, broader generalization and translational scalability remain constrained by ecological and structural realities unique to small ruminant production.

### Fragmentation, methodological diversity, and the need for species-specific synthesis

Recent investigations into AI applications for sheep and goats span diverse thematic domains. These include behavior and activity recognition [5, 13], reproductive event detection [14, 15], biometric identification using facial, acoustic, and retinal modalities [16–19], health and disease detection [8–10], growth and production trait prediction [20–23], and environmental or system-level monitoring [24]. A comprehensive list of all included studies, categorized by domain, is provided in Supplementary Tables S1 through S6.

The expanding literature is characterized by substantial thematic imbalance, with research efforts disproportionately concentrated in behavioral monitoring and individual identification tasks, collectively accounting for nearly half of the corpus, while health prognosis, production modeling, growth prediction, and genomic integration remain comparatively underrepresented despite their critical importance to sustainable production. Quantitative details of study distribution are provided in the Results section.

Beyond thematic fragmentation, the literature exhibits marked methodological heterogeneity. Studies differ considerably in sensing platforms, data dimensionality, sample size, breed composition, environmental context, algorithmic architecture, cross-validation schemes, use of independent test sets, and reporting transparency. Performance metrics vary across accuracy, precision, recall, F1-score, AUC, regression-based R^2^, and relative error measures, complicating cross-study comparability. Moreover, a substantial proportion of published studies lack rigorous external validation procedures, raising concerns regarding generalizability across farms, breeds, and climatic conditions [8, 11].

Despite the rapid growth of AI-related publications–with 92 studies identified between 2020 and 2025–no comprehensive synthesis has systematically evaluated methodological rigor, comparative model performance, translational readiness, and systemic adoption barriers specifically within sheep and goat production systems. Existing reviews either focus on single domains (e.g., behavior recognition) or extrapolate from cattle-focused PLF literature [4], overlooking the unique structural and ecological constraints of small ruminant systems. A species-specific and critically integrative review is therefore essential to consolidate current knowledge and guide future research directions.

### Aim and contribution of this review

In response to methodological heterogeneity, thematic imbalance, and uneven translational progress identified across the literature, the present study adopts a Systematic Narrative Review framework to provide a comprehensive and critically integrated synthesis of AI applications in sheep and goat production systems. The review period was defined as January 2020 to December 2025 to capture the contemporary AI paradigm–characterized by widespread adoption of deep learning architectures, transformer-based models, and large-scale computer vision frameworks–while minimizing inclusion of legacy rule-based systems no longer representative of current methodological standards. This boundary is explicitly incorporated into the eligibility criteria (Sect. "Search strategy and data acquisition").

This review makes several distinct contributions. First, it systematically maps the intellectual landscape of the field, organizing 92 peer-reviewed studies published between 2020 and 2025 into coherent thematic domains while quantifying temporal growth and research distribution patterns. Second, it synthesizes reported performance metrics across six application areas–behavior recognition, individual identification, health monitoring, growth prediction, genomics, and production systems–enabling structured comparison of algorithmic efficacy while contextualizing results within differences in study design and validation rigor. Third, it critically evaluates methodological robustness–including dataset characteristics, validation protocols, and external testing–to assess the maturity and generalizability of current AI solutions.

Beyond methodological evaluation, this review interrogates translational readiness by examining technical constraints, economic feasibility, infrastructural requirements, and socio-institutional barriers that may hinder large-scale adoption in extensive and smallholder production contexts. By integrating empirical performance evidence with implementation considerations, the study bridges the gap between experimental innovation and practical deployment.

Ultimately, this work provides a forward-looking research roadmap aimed at fostering scalable, welfare-oriented, economically viable, and environmentally sustainable integration of AI technologies within small ruminant production systems. In doing so, this review positions AI not merely as a technological advancement, but as a strategic instrument capable of addressing the structural vulnerabilities of small ruminant–enhancing resilience, productivity, and sustainability in a sector critical to global food security and rural livelihoods.

## Methods

### Protocol and reporting framework

This review was conducted in accordance with the PRISMA 2020 Statement [25] to ensure transparent and structured reporting of study identification, screening, eligibility assessment, and evidence synthesis. Given the pronounced methodological heterogeneity, variability in reported performance metrics, and diversity of sensing modalities and algorithmic architectures characterizing Artificial Intelligence (AI) applications in small ruminant systems, a Systematic Narrative Review (SNR) framework was adopted. This design is particularly appropriate for emerging technological domains in which quantitative meta-analysis is constrained by inconsistent outcome definitions, non-standardized validation procedures, and limited cross-study comparability.

A review protocol was developed a priori outlining the research objectives, eligibility criteria, search strategy, screening procedures, and data extraction plan. The protocol was registered with the Open Science Framework (OSF) on March 7, 2026, following the initial database search (August 2025) and a supplementary search (March 2026), but prior to formal screening and data extraction. The supplementary search was designed to ensure comprehensive coverage of studies published through December 2025, as the initial search (August 2025) did not capture the final months of that year. We therefore characterize this as partially prospective registration and acknowledge this as a limitation of the current study design. The protocol is available at: https://osf.io/vfpqk (DOI: 10.17605/OSF.IO/VFPQK) [26].

Although technology-focused livestock reviews are generally not eligible for registration in clinical registries such as PROSPERO, registration in OSF ensures transparency, provides a permanent and citable record of the methodology, and aligns with open science practices.

### Search strategy and data acquisition

The literature search was conducted across three major multidisciplinary databases: Web of Science, Scopus, and PubMed. These platforms were selected due to their broad coverage of peer-reviewed research spanning artificial intelligence, computer science, agricultural engineering, and animal production sciences. Although specialized agricultural databases such as AGRICOLA and CAB Abstracts may index additional region-specific material, comparative evidence suggests that the combined use of the three selected databases captures the substantial majority of high-impact interdisciplinary research. The potential omission of non-English or regionally indexed publications is acknowledged as a limitation.

To maximize sensitivity and avoid prematurely excluding relevant cross-species or comparative studies, the initial search strategy adopted a broad livestock-related terminology framework, intentionally incorporating terms for cattle, poultry, and swine alongside sheep and goat terms. This does not reflect ambiguity about the review's scope; it is a deliberate methodological choice to capture studies involving sheep or goats within broader multi-species datasets, consistent with standard systematic review practice. Species-specific restriction was applied during the structured screening phase (Sect. "Study selection and refinement for small ruminants"). This approach ensured comprehensive capture of AI applications across animal production systems before species-specific refinement. The deliberate inclusion of multiple livestock categories at the search stage was intended to reduce selection bias and prevent the inadvertent omission of studies that may involve sheep and goats within broader multi-species datasets.

Systematic searches were conducted in late August 2025 and yielded 11,035 records prior to deduplication (Scopus: 2,978; PubMed: 1,490; Web of Science: 6,567). To capture studies published after this initial search date and within the defined review window (through December 2025), a targeted supplementary search was conducted in early March 2026 using the identical Boolean strings, with date filters restricted to the period September 2025 through December 2025, immediately following the August 2025 primary search cutoff to ensure complete and non-overlapping coverage of the defined review window, subject to the same eligibility screening. All records were imported into EndNote X20 for bibliographic management, where automated and manual duplicate detection based on title, authorship, and DOI resulted in the removal of 1,648 duplicates, leaving 9,387 unique studies. Subsequent data cleaning and structured record management were performed using Spider within the Anaconda environment, facilitating harmonization of heterogeneous metadata formats and preparation for screening.

A dual-concept Boolean strategy was applied, combining: (i) AI-related terms ("Artificial Intelligence", "Machine Learning", "Deep Learning", "Neural Network*", "Computer Vision", "Natural Language Processing", "Large Language Model*") with (ii) livestock-related terms ("Precision Livestock Farming", "Cattle", "Bovine", "Swine", "Pig", "Poultry", "Sheep", "Ovis aries", "Goat", "Capra hircus"). Each database was queried using its native syntax and field tags to maximize retrieval precision. The verbatim search strings applied to each database were as follows:

- Web of Science (Advanced Search; Topic field tag TS =, covering title, abstract, author keywords, and Keywords Plus):
  - TS = ("Artificial Intelligence" OR "Machine Learning" OR "Deep Learning" OR "Neural Network*" OR "Computer Vision" OR "Natural Language Processing" OR "Large Language Model*") AND TS = ("Precision Livestock Farming" OR "Cattle" OR "Bovine" OR "Swine" OR "Pig" OR "Poultry" OR "Sheep" OR "Ovis aries" OR "Goat" OR "Capra hircus") AND PY = (2020–2025) AND DT = (Article) AND LA = (English)
- Scopus (Advanced Search; TITLE-ABS-KEY field, covering title, abstract, and author keywords; loose phrase matching via double quotation marks; all filters applied inline within the query string):
  - TITLE-ABS-KEY("Artificial Intelligence" OR "Machine Learning" OR "Deep Learning" OR "Neural Network*" OR "Computer Vision" OR "Natural Language Processing" OR "Large Language Model*") AND TITLE-ABS-KEY("Precision Livestock Farming" OR "Cattle" OR "Bovine" OR "Swine" OR "Pig" OR "Poultry" OR "Sheep" OR "Ovis aries" OR "Goat" OR "Capra hircus") AND PUBYEAR > 2019 AND PUBYEAR < 2026 AND DOCTYPE("ar") AND LANGUAGE("english")
- PubMed (Advanced Search; combining MeSH controlled vocabulary with [tiab] free-text field tags to maximize both precision and sensitivity; MeSH terms exploit automatic Explode functionality for hierarchical term expansion; notably, "Livestock"[MeSH] encompasses all domesticated animals including equines, which may retrieve records beyond the intended scope — these additional records are addressed through species-specific screening applied at the title and abstract stage (Sect. "Study selection and refinement for small ruminants"); [tiab] tags capture emerging terminology not yet indexed in MeSH, such as "Large Language Model"; all filters applied inline within the query string):
  - ("Artificial Intelligence"[MeSH] OR "Machine Learning"[MeSH] OR "Deep Learning"[MeSH] OR "Artificial Intelligence"[tiab] OR "Machine Learning"[tiab] OR "Deep Learning"[tiab] OR "Neural Network*"[tiab] OR "Computer Vision"[tiab] OR "Natural Language Processing"[tiab] OR "Large Language Model*"[tiab]) AND ("Livestock"[MeSH] OR "Cattle"[MeSH] OR "Swine"[MeSH] OR "Poultry"[MeSH] OR "Sheep"[MeSH] OR "Goats"[MeSH] OR "Precision Livestock Farming"[tiab] OR "Cattle"[tiab] OR "Bovine"[tiab] OR "Swine"[tiab] OR "Pig"[tiab] OR "Poultry"[tiab] OR "Sheep"[tiab] OR "Ovis aries"[tiab] OR "Goat"[tiab] OR "Capra hircus"[tiab]) AND ("2020/01/01"[PDAT]:"2025/12/31"[PDAT]) AND English[lang] AND Journal Article[pt].

The use of database-native syntax ensured that each query was optimized for the respective retrieval architecture: the TS = field tag in Web of Science indexes across four metadata fields simultaneously; TITLE-ABS-KEY in Scopus applies loose phrase matching appropriate for synonym-rich terminology; and the PubMed strategy combined MeSH controlled vocabulary – which activates automatic hierarchical term expansion (Explode) – with free-text [tiab] terms to capture contemporary AI terminology not yet fully integrated into MeSH indexing. Date and document-type filters were applied inline within each query string rather than through post-hoc interface filters, ensuring that the restrictions are fully reproducible independent of interface settings. Language was restricted to English in all three databases at the query level.

Eligibility criteria were then applied in a staged manner. Studies were limited to publications between January 2020 and December 2025 to ensure focus on contemporary AI paradigms – including deep learning architectures, transformer-based models, and advanced computer vision frameworks – while minimizing inclusion of legacy rule-based approaches no longer representative of current methodological standards. Non-English publications were excluded to maintain analytical consistency and data extraction reliability, as translation introduces additional risk of misinterpretation of technical details. We acknowledge this decision may have led to the omission of relevant research from regions such as the Middle East, North Africa, South America, and parts of Asia, where small ruminant production is economically and culturally significant but research may be published in Arabic, Spanish, Portuguese, or Chinese. This represents a recognized limitation, further discussed in Sect. "Discussion" (Limitations).

Species-specific restriction to sheep (*Ovis aries*) and goats (*Capra hircus*) was implemented during the screening phase (Sect. "Study selection and refinement for small ruminants"). This two-stage strategy — broad capture followed by targeted refinement — allowed comprehensive identification of potentially relevant studies while preserving alignment with the defined scope of small ruminant production systems.

Studies were included if they: (i) were peer-reviewed original research articles; (ii) published between January 2020 and December 2025; (iii) written in English; (iv) focused on sheep (*Ovis aries*) or goats (*Capra hircus*) as the primary or explicitly included species; (v) employed artificial intelligence, machine learning, or deep learning as a core methodological component; and (vi) addressed applications directly relevant to livestock production contexts (e.g., behavior monitoring, health detection, identification, production prediction).

### Study selection and refinement for small ruminants

Building on the cleaned dataset, a structured multi-stage screening process was conducted to identify original research specifically addressing AI applications in sheep and goat production systems.

All 9,387 unique records were managed in EndNote X20, where duplicate detection and metadata verification ensured dataset integrity. Temporal (January 2020—December 2025) and language (English only) filters were applied as predefined eligibility criteria. For records with incomplete metadata, publication year information was programmatically verified using structured text parsing in Python.

To refine the dataset toward small ruminant research while maintaining search sensitivity, a species-classification procedure was implemented. Keyword dictionaries representing major livestock categories (cattle, poultry, pigs, sheep, and goats) were applied to titles and abstracts. Studies were initially categorized according to species term frequency; however, this automated classification served only as a screening aid rather than a definitive exclusion criterion.

Importantly, studies involving multiple livestock species were retained if sheep or goats were explicitly included and if species-specific data or analyses could be identified at the abstract or full-text level. The automated keyword-based species classification served exclusively as a screening aid to prioritize records for human review; it was not used as a standalone exclusion criterion. All records flagged as potentially relevant, including those with ambiguous species terms, were retained for human screening. A random sample of 200 records classified as non-ruminant by the automated procedure was manually reviewed to estimate false negative rates; 6 records (3%) were found to involve sheep or goats as a secondary species and were re-included for full-text assessment. This audit confirmed that the automated classification adequately minimized false negative exclusions.

This species-refinement stage yielded 3,474 records in which sheep or goats were identified as a primary or explicitly included research focus.

Non-original publications–including reviews, meta-analyses, conference abstracts, editorials, short communications, and book chapters–were subsequently excluded, removing 522 records and leaving 2,952 original research articles.

An additional application-oriented screening step retained only studies in which artificial intelligence constituted a central methodological component and was directly applied to livestock production contexts (e.g., detection, prediction, monitoring, classification, health, welfare, production, or identification). This refinement reduced the dataset to 777 records.

Titles and abstracts were then screened using Rayyan [27] and Elicit [28] to facilitate structured evaluation. Rayyan was used exclusively as a collaborative platform for managing and recording screening decisions made by human reviewers; it did not make autonomous inclusion or exclusion decisions. Elicit was employed as a supplementary tool to assist with structured abstract summarization; all final eligibility decisions were made by human reviewers. Two independent reviewers assessed eligibility, with disagreements resolved through consensus discussion. Inter-reviewer agreement for title and abstract screening was assessed using Cohen's kappa (κ = 0.81, 95% CI: 0.76–0.86), indicating strong agreement; for full-text assessment, kappa was κ = 0.78. This stage identified 126 studies in which sheep or goats were clearly the dominant analytical focus and AI methods were substantively implemented.

Full-text assessment was conducted to exclude studies lacking species-specific data, insufficient methodological detail, or peripheral AI application. The final corpus comprised 92 peer-reviewed original research articles meeting all predefined inclusion criteria.

**Fig. 1:**
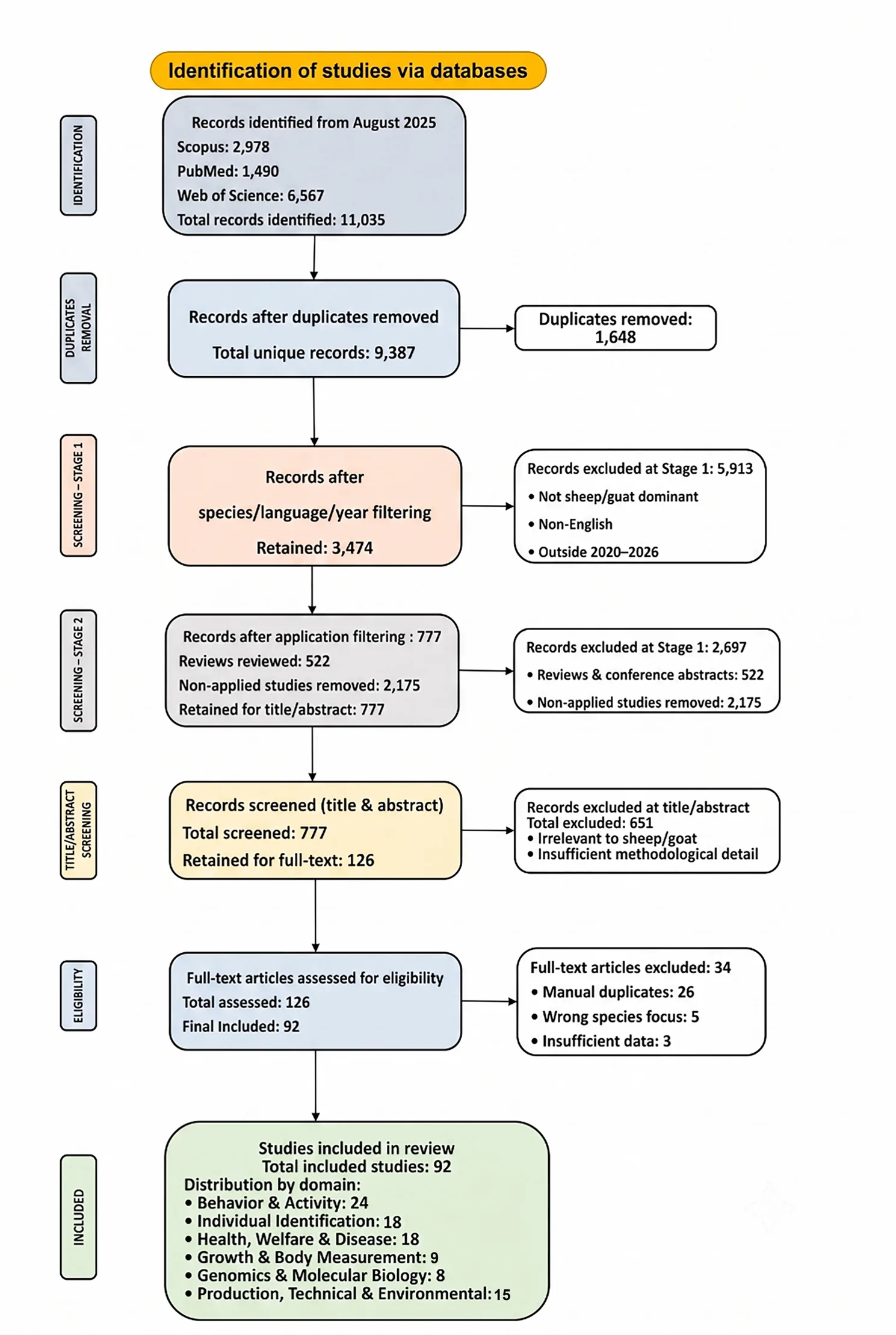
PRISMA 2020 flow diagram of the systematic review process, detailing the identification, screening, eligibility, and inclusion stages for studies on AI applications in sheep and goat production (2020–2025). A total of 92 studies met the inclusion criteria

Throughout the workflow, Spider within the Anaconda environment supported database harmonization, keyword-assisted species tagging, structured filtering, and dataset export. Rayyan and Elicit facilitated collaborative screening, conflict resolution, and final verification. This integrated, staged approach (summarized in Fig. 1) prioritized search sensitivity in early phases and specificity in later refinement, thereby strengthening methodological transparency and reproducibility.

### Data extraction and thematic categorization

A structured data extraction framework was developed and implemented in Microsoft Excel to ensure consistent and transparent capture of study-level information across all 92 included articles. The extraction template was predefined prior to full data collection and refined during pilot testing on a subset of studies to ensure completeness and clarity.

For each article, bibliographic information was recorded, including first author, year of publication, journal, and country of the corresponding author where available. Study-level characteristics were extracted to identify research objectives, target species (sheep, goats, or both), and production context (e.g., dairy, meat, extensive, intensive systems).

To enable structured synthesis, studies were classified into six thematic domains derived from iterative scoping of the literature: (i) Behavior and Activity Recognition; (ii) Individual Animal Identification; (iii) Health, Welfare, and Disease; (iv) Growth, Weight, and Body Measurement; (v) Genomics and Molecular Biology; and (vi) Technical Methods, Production, and Environment. Domain assignment was based on the primary research objective explicitly stated in the study. For multi-domain studies, classification reflected the dominant analytical focus, while secondary applications were noted descriptively to preserve contextual nuance.

The six thematic domains differ systematically in the types of traits analyzed and ML architectures commonly applied. For example, Behavior domain studies typically employ CNNs or LSTMs on accelerometer or video data to classify activities such as grazing, lying, or ruminating; Identification studies use CNNs or Vision Transformers on facial or retinal images; Health studies apply Random Forest, SVM, or neural networks to clinical records, sensor data, or images; Growth studies apply tree-based ensembles (e.g., Random Forest, ExtraTree, XGBoost) or regression models to morphometric and meteorological data; Genomics studies leverage Random Forest, ANNs, or hybrid deep learning (e.g., CNN-GRU) on transcriptomic or SNP data; and Technical domain studies employ CNNs, Swin-Transformers, or SVR on UAV/satellite imagery, milk spectra, or farm records. Domain-specific methodological patterns are highlighted in Sects. "AI applications in behavior and activity recognition"–"AI applications in technical methods, production, and environment". Technical attributes were systematically extracted, including AI methodologies (e.g., convolutional neural networks, YOLO-based detectors, Random Forest models, Support Vector Machines, long short-term memory networks, and transformer architectures), input data modalities (images, video, accelerometer signals, GPS data, acoustic recordings, genomic or transcriptomic data), and reported sample sizes. The distribution of input data types across included studies is summarized in Figure S15. Where available, information regarding validation strategy (e.g., cross-validation, independent test sets) was also recorded.

Outcome measures were captured to summarize reported performance metrics, including accuracy, precision, recall, F1-score, correlation coefficients, mean absolute error, or other domain-specific indicators. Author-reported limitations and implementation constraints were documented to inform cross-study comparison and thematic synthesis.

To enhance reliability and minimize extraction bias, data collection was performed by a primary reviewer (AAG) and independently verified by a second reviewer for a randomly selected 20% subset of studies (n = 18). Inter-reviewer agreement for the extraction subset was 94.4% (17/18 studies fully concordant); the one discordant case involved domain classification assignment and was resolved by consensus discussion. This partial double-extraction approach balances methodological rigor with feasibility and is consistent with established practices in systematic narrative reviews.

### Data synthesis

Substantial heterogeneity was observed across the 92 included studies, including variation in study design, AI methodologies, input data modalities, validation procedures, outcome measures, and reporting practices. The absence of standardized effect size metrics and comparable experimental frameworks precluded meaningful statistical aggregation. Accordingly, formal quantitative meta-analysis was not considered methodologically appropriate.

A structured narrative synthesis was therefore conducted, consistent with established methodological guidance for synthesizing complex and heterogeneous evidence [29]. Narrative synthesis is particularly suitable in interdisciplinary domains characterized by methodological diversity and contextual variability, where the objective extends beyond effect estimation to understanding patterns, relationships, and implementation contexts.

The synthesis was organized according to the six thematic domains defined during data extraction: Behavior and Activity Recognition; Individual Animal Identification; Health, Welfare, and Disease; Growth, Weight, and Body Measurement; Genomics and Molecular Biology; and Technical Methods, Production, and Environment. Within each domain, studies were comparatively examined with attention to associations among research objectives, data modalities, AI architectures, validation strategies, and reported performance metrics.

Rather than pooling quantitative outcomes, the analysis focused on identifying convergent methodological trends, performance variability, recurring limitations, and domain-specific knowledge gaps. Cross-domain comparison further enabled evaluation of structural patterns influencing generalizability and translational potential. This domain-oriented synthesis framework supports structured interpretation of heterogeneous evidence while preserving contextual nuance and study-specific characteristics.

### Software, packages, and data visualization

All data processing, statistical analysis, and visualization were performed using Python (version 3.13.9) within the Anaconda distribution (Anaconda Inc., Austin, TX, USA). The integrated development environment Spyder (version 5.5.1) was employed for script development, execution, and debugging throughout the systematic review workflow [30].

Data manipulation and analysis were conducted using the Pandas (version 2.2.0) [31] library. Bibliographic record management and initial screening were facilitated by EndNote X20 (Clarivate Analytics, Philadelphia, PA, USA), while collaborative screening and conflict resolution were supported by Rayyan (Ouzzani et al., 2016) [27] and Elicit [28].

All figures were generated using the Matplotlib (version 3.8.2) [32] and Seaborn (version 0.13.2) [33] visualization libraries. The PRISMA flow diagram (Fig. 1) was constructed using custom Python scripts with Matplotlib patches to ensure accurate representation of the multi-stage screening process. Domain distribution visualizations employed donut charts with integrated percentage labels and external legends. Heatmaps, scatter plots, and box plots were generated to illustrate methodological patterns, performance variability, and cross-domain comparisons. Where applicable, NetworkX (version 3.2.1) [34] was used for preliminary exploration of keyword co-occurrence networks.

All main figures (Figs. 1, 2, 3 and 4) and supplementary figures (Figures S1–S19) were created following the same approach, ensuring consistency and a high-quality output (600 dpi) suitable for publication. The complete set of Python scripts used for data processing, statistical analysis, and figure generation is available from the corresponding author upon reasonable request, ensuring full reproducibility of all graphical outputs presented in this review.

**Fig. 2:**
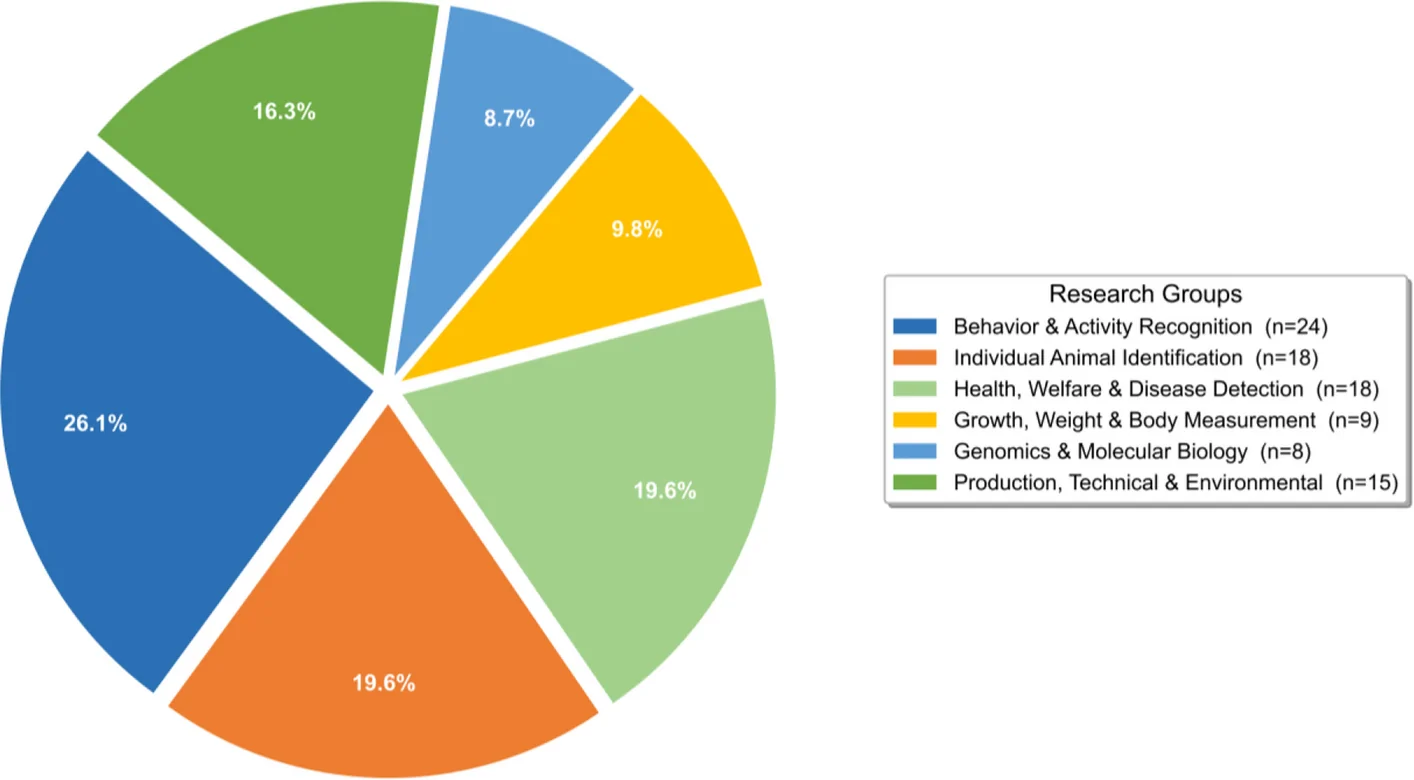
Domain distribution of artificial intelligence research in sheep and goat production (*n* = 92 studies)

**Fig. 3:**
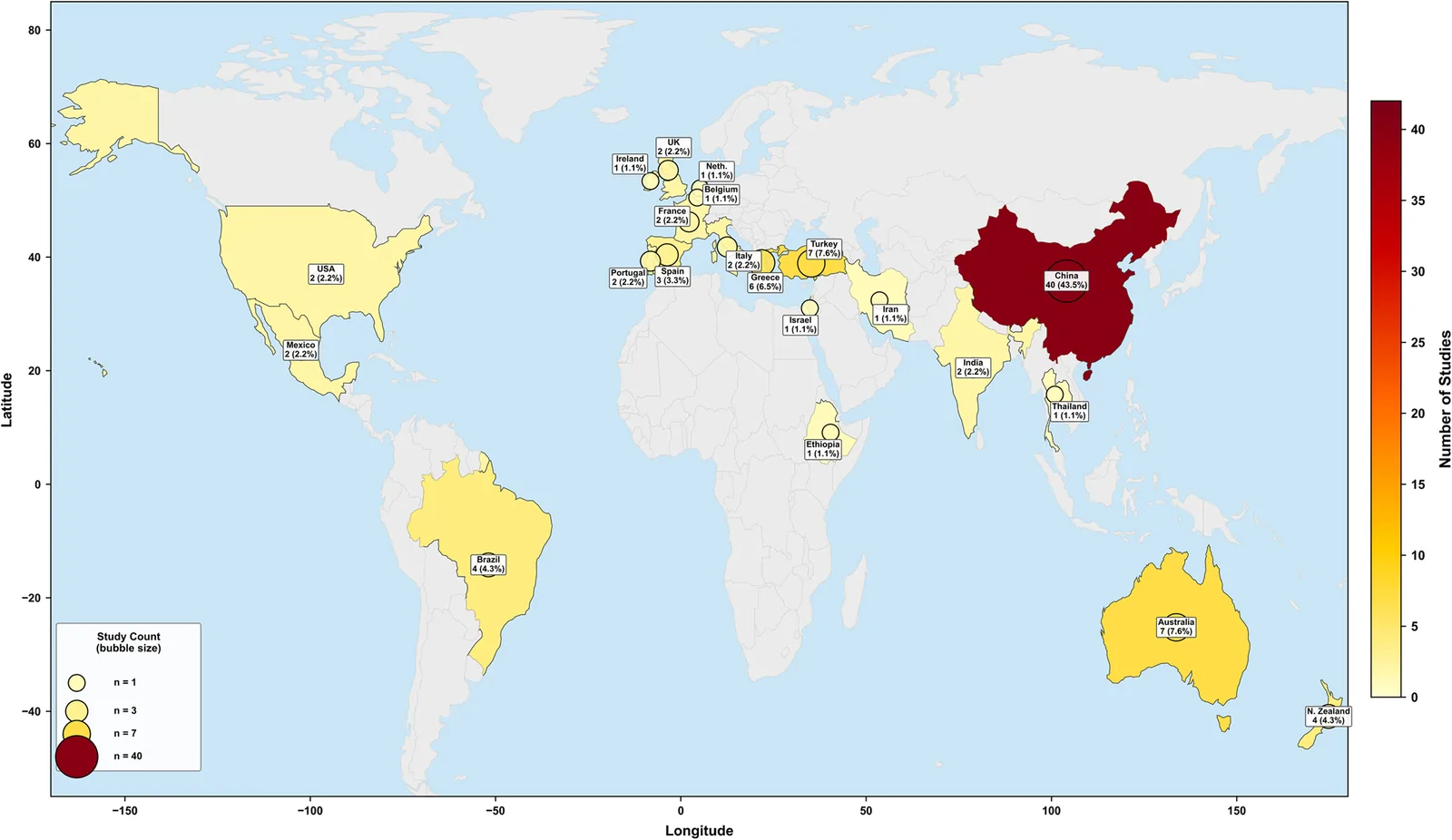
World Map—Geographic distribution of corresponding author affiliations across 92 included studies (2020–2025). Studies originate from 21 countries | Circle size & color represent study count

**Fig. 4:**
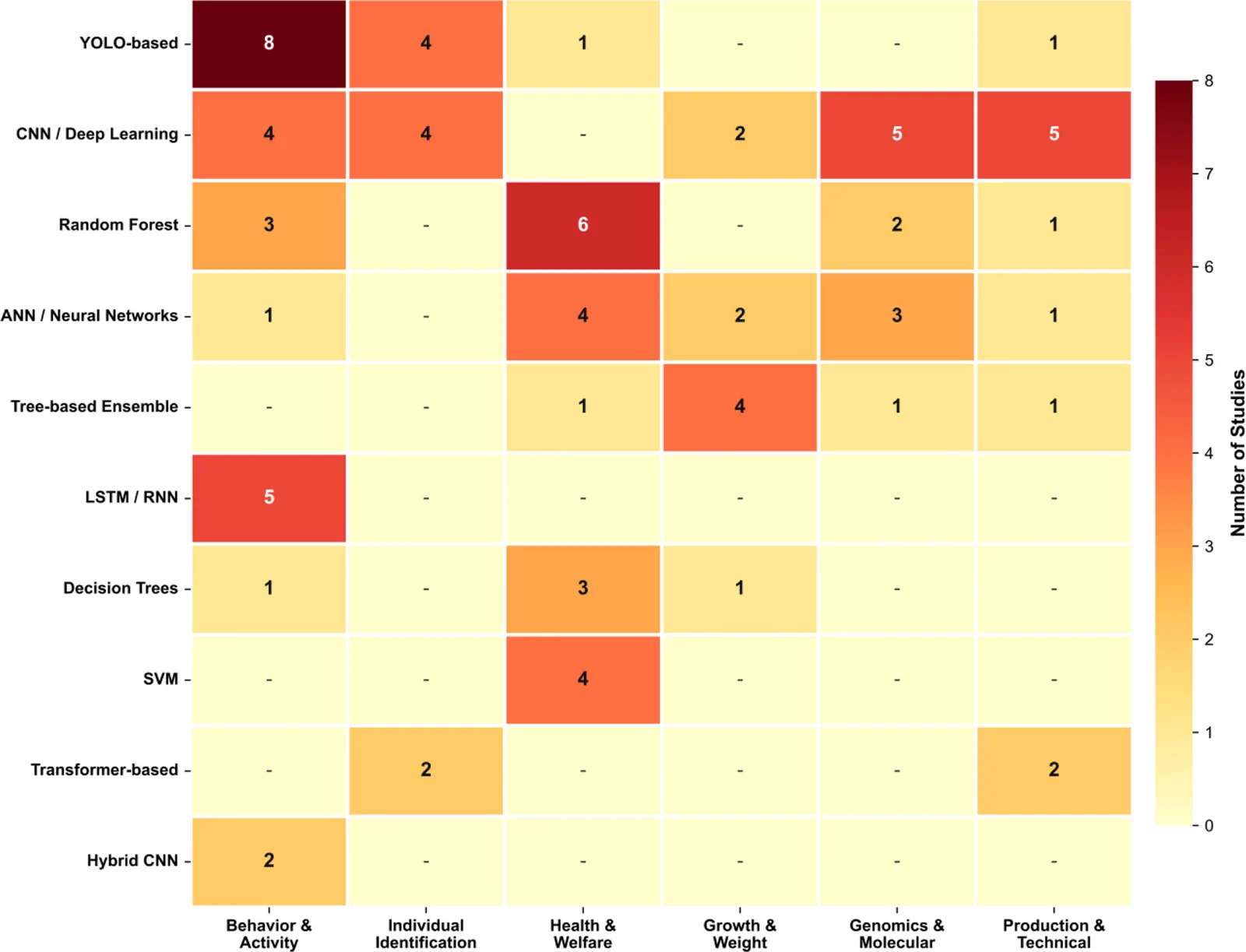
Heatmap illustrating the distribution and prevalence of artificial intelligence techniques across the six application domains in sheep and goat research (*n* = 92 studies). The color intensity represents the number of studies employing a given technique within each domain, based on data extracted from Supplementary Tables S1–S6. YOLO is concentrated in Behavior & Activity (*n* = 8) and Individual Identification (*n* = 4). LSTM/RNN appears exclusively in Behavior & Activity (*n* = 5). SVM is nearly exclusive to Health & Welfare (*n* = 4). CNN/DL is the most cross-cutting technology, appearing in Genomics (*n* = 5), Production (*n* = 5), Behavior (*n* = 4), and Identification (*n* = 4). Random Forest dominates Health & Welfare (*n* = 6)

### Quality assessment

Methodological quality of included studies was assessed using domain-specific appraisal tools adapted for AI applications in livestock research. Given the absence of a universally accepted quality assessment tool for AI-based animal studies, a customized framework was developed based on key methodological dimensions: (i) dataset characteristics and sample size adequacy, (ii) clarity of AI methodology description, (iii) validation strategy (e.g., cross-validation, independent test sets), (iv) reporting of performance metrics, and (v) consideration of generalizability and external validity. The final instrument comprised eight domains (D1–D8), each scored 0–2 (0 = absent/inadequate; 1 = partially addressed; 2 = fully addressed), yielding a composite score of 0–16. Based on a priori thresholds, studies were categorized as: Low quality (≤ 7), Moderate quality (8–12), or High quality (≥ 13). The complete quality assessment instrument and study-level ratings for all 92 studies are provided in Supplementary Table S8 (Parts A–F). Two independent reviewers rated each study, with disagreements resolved through consensus. Quality assessment results are presented narratively; study-level scores across all eight domains are reported in Supplementary Table S8 (Part B) and summarized by tier in Supplementary Tables S1–S6 (Quality Tier column). The overall quality distribution is illustrated in Figure S1, the distribution of composite quality scores in Figure S3, mean domain scores across research groups in the heatmap (Figure S4), and the spread of scores within each group in box plots (Figure S6). Strength patterns consistently observed across high-quality studies are illustrated in Figure S17, and yearly publication trends in Figure S14.

## Results

### Study selection and literature overview

Table 1 provides a domain-level summary of reported performance metrics; detailed study-level characteristics (species, sample sizes, AI methods, input modalities, validation strategies, and quality tiers) are provided in Supplementary Tables S1–S6.

**Table 1:** Summary of reported performance metrics across 92 included studies, categorized by application domain

Application Domain	N	Reports	Primary Metric(s)	Range	Key Representative Findings
Classification-based domains					
Behavior & Activity Recognition	24	21	Accuracy	66.7–100%	CNN-LSTM 99.3% [13]; FESS-YOLOv8n [5]; BLSTM F1 = 0.84 [12]; Microcontroller DT > 91% [35]
Individual Animal Identification	18	16	Accuracy	93.3–99.9%	DWT-GoatNet 99.89% [36]; ViT-Sheep 97.9% [16]; MobileViTFace 97.13% [37]; Retinal 99% [17]
Health, Welfare & Disease	18	8 (Acc)	Accuracy	62.0–99.0%	miRNA + ML 99% [38]; SVM mastitis 96.3% [39]
		4 (AUC)	AUC	0.82–0.99	Ensemble model AUC 0.99 [40]
		2 (F1)	F1-score	0.84–0.87	BLSTM F1 = 0.84 [12]
Genomics & Molecular Biology	8	3 (Acc)	Accuracy	95.6–98.9%	scRNA CNN-GRU 98.89% [41]
		2 (AUC)	AUC	0.97	4-gene ANN AUC 0.97 [42]
Technical, Production & Environment	15	8 (Acc)	Accuracy	86.0–99.0%	UAV CNN 98% recall [43]; Swin-T mAP 94.3% [44]
		4 (F1/mAP)	F1-score/mAP	0.83–0.96	SMOreg *r* = 0.96 [45]; GPS + Landsat RF [24]
Regression-based domain					
Growth & Body Measurement	9	3	R^2^	0.78–0.98	ExtraTree R^2^ = 0.81 [22]; NN-GBLUP methane R^2^: 0.09 → 0.30 [23]
		6	MAPE/Relative Error	< 6%	3D point cloud < 4% error [20]
Overall Summary	92	77 (83.7%)	Mixed (see notes)	62.0–100%	Quality: 27 High (29.3%), 60 Moderate (65.2%), 5 Low (5.4%)

Performance metrics are reported as they appear in the original studies. Classification-based domains (Behavior, Identification, Health, Genomics, Technical) and the regression-based domain (Growth) are presented separately to avoid inappropriate mixing of metric types. Health and Genomics domains include studies reporting accuracy, AUC, and F1-score; these are shown as distinct sub-rows. The Growth domain reports only native regression metrics (R^2^, MAPE, relative error); no conversion to "accuracy equivalents" has been attempted. No overall mean accuracy is reported due to heterogeneity of tasks, datasets, validation strategies, and metric types across domains. The "Accuracy" values shown for Health, Genomics, and Technical domains are derived only from studies that explicitly reported accuracy as their primary metric; studies reporting only AUC, F1, mAP, or regression metrics are excluded from those accuracy figures. The quality distribution (High/Moderate/Low) is derived from the eight-domain quality assessment (Supplementary Table S8, Part C). N = total number of studies in domain; Reports = number of studies reporting quantitative metrics suitable for synthesis; Acc = accuracy; AUC = area under the curve; F1 = F1-score; mAP = mean average precision; R^2^ = coefficient of determination; MAPE = mean absolute percentage error. Quality classifications follow Supplementary Table S8 (Parts A–F), based on eight-domain scores (0–16) with thresholds: High ≥ 13, Moderate 8–12, Low ≤ 7

The systematic search identified 11,035 records across Web of Science, Scopus, and PubMed. Following deduplication (*n* = 1,648), temporal restriction (2020–2025), species-specific classification, exclusion of non-original publications (*n* = 522), application-focused filtering, and structured screening in Rayyan [27], a total of 92 peer-reviewed original studies met all eligibility criteria and were included in the final synthesis. The complete selection process is presented in the PRISMA flow diagram (Fig. 1).

A critical overarching caveat applies to all performance figures presented in this section: direct cross-domain comparison of numerical accuracy values is not methodologically justified due to fundamental differences in task types (classification vs. regression), data modalities (sensor, image, genomic), class balance, validation strategies, and environmental conditions. The descriptive domain-specific averages provided below are intended only to illustrate central tendencies within each domain and should not be interpreted as pooled estimates or as evidence of algorithmic superiority across applications.

The characteristics of all included studies–including bibliographic information, primary objectives, AI techniques, sample sizes, and key outcomes–are detailed in Supplementary Tables S1–S6, organized according to the six thematic domains established during data extraction. The distribution of studies across these domains is illustrated in Fig. 2.

Analysis of the literature revealed a pronounced concentration of AI research in two primary domains.

Behavior and Activity Recognition emerged as the dominant focus, comprising 26.1% of the included studies (*n* = 24), followed by Individual Animal Identification at 19.6% (*n* = 18). Health, Welfare, and Disease accounted for 19.6% (*n* = 18), Growth, Weight, and Body Measurement for 9.8% (*n* = 9), Genomics and Molecular Biology for 8.7% (*n* = 8), and Production, Technical Methods, and Environment formed a methodologically distinct cluster at 16.3% (*n* = 15). The ten most frequently reported application types are summarized in Figure S13, and species distribution across research groups is shown in Figure S11.

Geographic distribution of included studies is illustrated in Fig. 3 (choropleth map showing study counts per country); detailed country-level and continental

 breakdowns are provided in Figures S7 and S8, respectively; the intersection of country and research group is illustrated in Figure S19.China dominates the field (*n* = 40, 43.5%), followed by Turkey and Australia (*n* = 7, 7.6%).

### AI applications in behavior and activity recognition

As outlined in Sect. "Data extraction and thematic categorization", behavior recognition studies predominantly use CNNs and LSTMs on accelerometers or video data. Behavior and activity recognition emerged as the most extensively represented thematic domain, accounting for 24 of the 92 included studies (26.1%). Across this body of literature, artificial intelligence has been primarily employed to monitor sheep (*Ovis aries*) and goats (*Capra hircus*) as behavioral indicators of health status, welfare conditions, and reproductive events. Among the 21 studies that explicitly reported quantitative evaluation metrics suitable for synthesis, mean classification accuracy reached 92.4%, with reported values ranging from 66.7% to 100%. This considerable variability reflects differences in environmental settings, behavioral definitions, sensing modalities, data quality, and validation strategies rather than purely algorithmic superiority.

A clear methodological gradient is observable across literature. Studies reporting the highest predictive performance typically combined multi-modal sensor inputs–such as inertial measurement units positioned on different body regions or integrated vision-based pose estimation–with deep learning architectures capable of modeling spatio-temporal dependencies. Convolutional neural networks (CNNs), long short-term memory networks (LSTMs), and spatio-temporal graph convolutional models were frequently employed to capture both spatial posture characteristics and sequential movement dynamics. These integrated systems often achieved accuracies exceeding 95%, particularly in controlled or semi-controlled housing environments [13, 46].

In contrast, another subset of studies prioritized computational efficiency and practical deployability over maximal accuracy. Lightweight object detection frameworks and classical machine learning algorithms, including Decision Tree–based classifiers and ensemble approaches such as Random Forest, demonstrated performance levels around 90% while substantially reducing model size and energy requirements [6, 35]. Such approaches appear better aligned with on-animal wearable devices and edge-computing environments.

A number of investigations focused specifically on high-impact management events, including lambing detection [7, 14], estrus identification through mounting behavior [15], and peripartum behavioral changes [47]. These event-specific models frequently reported strong evaluation metrics, including high F1-scores and Matthews Correlation Coefficients. Nevertheless, rare-event detection studies were often based on relatively small or farm-specific datasets, limiting external generalizability.

Overall, the breadth of reported performance underscores the decisive influence of contextual and methodological factors on model outcomes. Environmental noise, occlusion, stocking density, and class imbalance were recurrently associated with reduced predictive stability [11, 12]. A particularly noteworthy infrastructure contribution is the openly available goat behavior dataset published by Mauny et al. [48], combining synchronized accelerometer recordings with expert-labelled ethogram annotations across multiple behavioral states. Unlike most datasets in this domain that remain proprietary or unpublished, this resource enables direct algorithm comparison, external replication, and model training without bespoke data collection – addressing one of the principal bottlenecks to translational progress identified in this review. A comprehensive overview of the 24 included studies is provided in Supplementary Table S1.

### AI applications in identification and facial recognition

As outlined in Sect. "Data extraction and thematic categorization", identification studies predominantly use CNNs or Vision Transformers on facial or retinal images.

Individual animal identification constitutes the second major thematic domain within AI applications in small ruminant systems, encompassing 18 studies. Non-invasive biometric approaches–particularly facial recognition–have emerged as a leading strategy, combining high classification accuracy with reduced stress and handling requirements. Across the 16 studies reporting quantitative performance metrics, mean accuracy reached 97.3%, with values ranging from 93.3% to 99.9%. This variability reflects differences in dataset size, image quality, animal age, and algorithmic architecture.

Facial recognition systems consistently leverage deep learning frameworks capable of extracting fine-grained visual features. Convolutional neural networks integrated with MTCNN and FaceNet modules have demonstrated accuracies exceeding 95% [49], while transformer-based architectures, such as Vision Transformer models enhanced with LayerScale, have achieved comparable or superior performance with fewer annotated samples [16]. Lightweight networks optimized for edge deployment have also been implemented to balance inference speed and computational load [50, 51].

Beyond visual modalities, alternative biometric strategies offer complementary solutions. Ntalampiras and Pesando [18] provide the most methodologically distinctive acoustic study: applying CNN to goat vocalisation spectrograms, they achieved 95.8% classification accuracy while uniquely incorporating Concept Relevance Propagation (CRP) – an explainable AI technique that maps which time–frequency features drove each classification decision. This XAI integration is significant beyond the accuracy figure: it renders model attention interpretable in acoustically meaningful terms, enabling practitioners to identify which vocalisations carry diagnostic weight and directly supporting clinical adoption. This is the only included identification paper combining competitive accuracy with post-hoc interpretability, and its CRP framework should serve as a benchmark for future acoustic biometric work. Acoustic identification achieved 95.8% accuracy [18], whereas retinal-based identification demonstrated near-perfect accuracies (99%) leveraging lifetime-stable vascular patterns [17].

Despite these encouraging results, a fundamental practical limitation of the majority of facial and biometric recognition systems reviewed here is the closed-set identification paradigm employed. In most studies, all animals whose images appear in the test set also contributed images to the training set, meaning the model is evaluated on recognizing known individuals rather than classifying new, unseen animals. In real farm deployment, new animals are continuously introduced, necessitating either full model retraining or open-set recognition architectures. Fewer than 20% of identification studies reviewed here addressed this deployment-critical limitation. Future work must prioritize open-set and few-shot learning approaches to achieve operational viability. Additionally, the high degree of morphological similarity among sheep or goat individuals can limit discriminative capacity, particularly in large flocks or across different breeds. Environmental factors, such as lighting conditions, occlusions, and variable animal postures, further contribute to performance heterogeneity [19, 47]. A comprehensive overview of the 18 included studies is provided in Supplementary Table S2.

### AI applications in health, welfare, and disease detection

Health, welfare, and disease detection represent the third major thematic cluster, comprising 18 studies. These applications aim to detect specific pathological or physiological conditions, often characterized by multifactorial etiology and subtle early-stage manifestations. Among the 14 studies reporting explicit quantitative performance metrics, mean accuracy reached 89.7%, with a notably wide range from 62.0% to 99.0%. The lower bound (62.0%) represents a reported specificity value (ZareBidaki et al., 2022) [52] rather than overall classification accuracy and should be interpreted accordingly. This broader variability reflects the inherent biological complexity of disease processes, heterogeneity of target conditions, and variation in data modalities.

At the individual-animal level, wearable sensor–based systems have primarily focused on detecting behavioral deviations associated with lameness and parasitic infections. Accelerometer-derived activity patterns analyzed through machine learning classifiers demonstrated moderate-to-high discrimination capacity [8, 10]. However, performance generally declined when attempting to detect early or subclinical conditions.

At the field and farm level, deployable diagnostic systems have emerged with practical implications. Smartphone-based image analysis for parasitological egg counting achieved high concordance with laboratory standards [9, 53], supporting targeted selective treatment strategies.

At the molecular scale, AI models have been applied to high-dimensional omics data. Circulating miRNA signatures enabled near-perfect discrimination of stress-related physiological states [38]. Infrared thermography combined with object detection frameworks demonstrated moderate accuracy for identifying subclinical mastitis [54].

At the population and systems level, predictive modeling extended toward herd benchmarking and epidemiological risk mapping. Machine learning algorithms modeled bulk-tank milk quality and mastitis prevalence with high reported accuracies [39, 55]. Epidemiological niche modeling demonstrated the capacity of AI to forecast disease distribution at continental scales [40].

A comprehensive overview of the 18 included studies is provided in Supplementary Table S3.

### AI applications in growth, weight, and body measurement

As outlined in Sect. "Data extraction and thematic categorization", growth studies apply tree-based ensembles (e.g., Random Forest, ExtraTree, XGBoost) or regression models to morphometric and meteorological data. Performance metrics in this domain are regression-based (R^2^, MAPE, relative error) and are not comparable to classification accuracy values reported in other domains.

Growth prediction, body weight estimation, and morphometric assessment constitute the fourth thematic cluster, encompassing 9 studies. Across the 9 included studies, performance metrics varied by study objective. Three studies reported coefficients of determination (R^2^) ranging from 0.78 to 0.98 (mean = 0.86). The remaining six studies reported mean absolute percentage error (MAPE) or relative measurement error, with several models achieving errors below 6%.

At the phenotyping level, non-contact 3D imaging systems have emerged as a prominent methodological approach. Multi-camera depth-sensing systems combined with point cloud–based deep learning architectures demonstrated low relative errors (often < 4%) across key morphometric traits [20]. Low-cost imaging platforms implemented in lambs achieved weight estimation errors below 6% [56].

Regression-based machine learning models have been extensively employed for predicting growth trajectories and live weight. Tree-based algorithms generally achieved R^2^ values ranging from approximately 0.78 to 0.81 when incorporating environmental and meteorological variables [21, 22]. Models integrating contextual features consistently outperformed those relying solely on biometric inputs.

Computer vision and deep learning frameworks have further enabled automated trait segmentation and classification. Transformer-based architectures and advanced convolutional networks demonstrated high detection and segmentation accuracy [57].

Emerging integrative models extend beyond phenotypic and environmental predictors to incorporate genomic and microbial information. Neural network–based GBLUP frameworks combining host genomic variation with rumen microbiome composition improved predictive accuracy for methane emissions and residual feed intake [23]. A comprehensive overview of the 9 included studies is provided in Supplementary Table S4.

### AI applications in genomics and molecular biology

Genomics and molecular biology constitute a methodologically sophisticated thematic cluster, comprising 8 studies that apply AI to high-dimensional omics datasets. Among the 5 studies reporting explicit quantitative performance metrics, mean accuracy reached 97.8%, with values ranging from 95.6% to 98.9%.

At the cellular level, a hybrid deep learning framework applied to single-cell RNA sequencing data achieved 98.89% accuracy in classifying goat granulosa cells as fertility-supporting or non-supporting [41]. At the gene-network level, integration of RNA sequencing, weighted gene co-expression network analysis, and supervised machine learning enabled investigation of gene–environment interactions associated with hypoxia adaptation [58].

In disease susceptibility, multi-omics integration with Random Forest models identified potential biomarkers associated with *Escherichia coli* F17 infection in sheep [59]. At the diagnostic level, an artificial neural network developed from four hub genes achieved 95.6% accuracy in diagnosing intrauterine growth restriction [42].

For complex trait prediction, neural network GBLUP frameworks integrating host genomic variation with rumen microbial composition improved prediction accuracy for methane emissions and residual feed intake [23]. At the multi-omics integration level for breed characterization, integration of genome and transcriptome data with neural network–based feature selection identified key functional genes underlying double-coated fleece formation [60]. A comprehensive overview of the 8 included studies is provided in Supplementary Table S5.

### AI applications in technical methods, production, and environment

The final thematic cluster, comprising 15 studies, extends AI applications beyond direct animal-level monitoring toward technical optimization, production modeling, and environmental assessment. Reported performance metrics indicate generally high predictive capacity, particularly in structured modeling tasks and image-based detection frameworks.

At the molecular and product-analysis level, foundation models and transfer learning approaches enabled prediction of peptide transportability (accuracy 0.89) and taste-profile classification (up to 0.99 accuracy) [61]. In production modeling, support vector–based regression achieved a correlation coefficient of 0.96 for total milk yield prediction [45].

Technical advancements in high-density and large-scale monitoring environments were also evident. Self-supervised learning improved individual localization accuracy without requiring additional labeled data [62]. UAV-based sheep detection systems increased recall rates up to 98% [43]. Transformer-based segmentation models applied to satellite imagery achieved improvements in mean intersection over union [63].

At the precision management level, lightweight two-stage machine learning systems demonstrated Matthews Correlation Coefficients up to 0.93 for lambing detection [14]. Decision support systems enabled feeding cost reductions of 5.5% through optimized grouping strategies [64].

At the landscape scale, integration of GPS tracking data with satellite imagery and Random Forest modeling enabled quantification of grazing impacts on vegetation dynamics [24]. In carcass processing, multi-scale dual attention U-Net architectures achieved 93.76% precision for hind leg segmentation [65]. Swin-Transformer–based classification of mutton multi-parts achieved mean average precision of 94.3% [44]. A comprehensive overview of the 15 included studies is provided in Supplementary Table S6.

#### Methodological landscape: AI techniques across domains

Figure 4 presents a heatmap illustrating the distribution of primary AI techniques across the six application domains; a ranked summary of the ten most frequently employed techniques is provided in Figure S12. Convolutional Neural Networks (CNNs) emerge as the most widely adopted architecture, appearing in 20 studies across all domains – most prominently in Genomics (*n* = 5), Production (*n* = 5), Behavior (*n* = 4), and Individual Identification (*n* = 4), with additional representation in Health and Disease (*n* = 1) and Growth (*n* = 1). YOLO-based detectors are predominantly concentrated in behavior recognition (*n* = 8) and identification tasks (*n* = 4), reflecting their suitability for real-time object detection. Classical machine learning methods, particularly Random Forest (*n* = 12) and SVM (*n* = 4), maintain a presence in health, growth, and production domains where tabular sensor data predominates. Transformer-based architecture, while less frequent overall (*n* = 4), show increasing adoption in identification and production tasks, indicating a methodological shift toward attention-based modeling. The temporal evolution of AI technique adoption across the review period (2020–2025) is illustrated in Figure S16.

### Summary of reported performance metrics

Table 1 provides a consolidated overview of reported performance metrics across the 92 included studies, categorized by application domain. Important note on metric interpretation: performance figures across domains are NOT directly comparable, as studies differ fundamentally in task type, data modality, outcome definition, and validation strategy. Classification metrics (accuracy, AUC, recall) reported for behavior recognition or identification tasks are not equivalent to regression metrics (R^2^, MAPE) reported for growth prediction, nor to diagnostic metrics reported for health applications. Among 77 studies (83.7%) reporting quantitative metrics, the distribution of reported performance scores by research group is illustrated in Figure S6, and the ten best-performing studies are ranked in Figure S18.

The final quality distribution across all 92 studies is: 27 high-quality (29.3%), 60 moderate-quality (65.2%), and 5 low-quality (5.4%) studies, based on the eight-domain quality assessment (Supplementary Table S8, Part C).

Mean classification accuracy for studies reporting accuracy as their primary metric ranged from 89.7% (Health domain, based on *n* = 8 accuracy-reporting studies) to 97.3% (Identification domain, based on *n* = 16 accuracy-reporting studies). These figures are descriptive domain-specific averages only, subject to the non-comparability caveat stated above. For domains where studies reported mixed metrics (e.g., AUC, F1, mAP in Health, Genomics, and Technical domains), no single mean accuracy is calculated. For the Growth domain, regression metrics (R^2^, MAPE, relative error) are reported separately and are not converted to accuracy equivalents (see Table 1). No overall mean accuracy across all domains is reported, as pooling classification and regression metrics—or even accuracy across fundamentally different classification tasks–is not methodologically justified. As shown in Table 1, the Health, Genomics, and Technical domains include studies reporting accuracy, AUC, F1-score, and mAP; these are presented as distinct sub-rows to avoid inappropriate mixing of metric types.

Performance variability was observed both within and across thematic domains. The highest reported accuracies were generally associated with structured tasks conducted under controlled experimental or semi-controlled farm conditions. For instance, multi-sensor fusion approaches for behavior classification achieved 99.3% accuracy [13], deep learning–based cellular phenotyping models reached 98.89% accuracy [41], and advanced facial recognition systems reported performance exceeding 99% [36].

In contrast, lower accuracy values were more frequently reported in studies addressing biologically complex or indirectly inferred outcomes. Tasks such as maternal success prediction [66] and subclinical health detection based on behavioral proxies [52] demonstrated reduced performance, reflecting variability in biological expression and indirect measurement strategies.

The overall quality distribution (Figure S1) shows that the majority of studies are of Moderate quality (60 studies, 65.2%), followed by High quality (27 studies, 29.3%) and Low quality (5 studies, 5.4%). The distribution of composite scores (Figure S3) reveals a mode at score 12 (*n* = 36, 39.1%). Mean domain scores (Figure S4) indicate that D6 (Experimental Design) is the weakest domain overall (grand mean 0.98). Validation strategy is the strongest predictor of study quality (Figure S5): studies with D3 = 2 (independent hold-out or cross-farm validation) are predominantly High quality (77.8%), whereas all D3 = 0 studies are Low quality. Box plots of quality scores by group (Figure S6) show that Growth & Measurement has the tightest distribution, while Behavior & Activity exhibits the widest spread and most outliers.

### Systemic barriers to AI adoption

Although the reviewed studies demonstrate substantial predictive capacity across behavioral monitoring, identification, health assessment, growth modeling, genomics, and production systems, cross-domain synthesis indicates recurring structural constraints that limit scalability beyond experimental or pilot implementations. These constraints consistently emerge across thematic clusters and can be categorized into technical/data-related, methodological, and socioeconomic dimensions. Barrier frequencies reported in the abstract (e.g., limited dataset diversity: *n* = 30; class imbalance: *n* = 16; poor generalization: *n* = 13; occlusion/environment: *n* = 11; hardware constraints: *n* = 9) were derived from narrative coding of limitation sections within each included study, cross-referenced against domain-specific data in Supplementary Tables S1–S6.

#### Technical and data-related constraints

A dominant cross-cutting limitation is the absence of standardized and interoperable data ecosystems. Across studies, substantial heterogeneity was observed in sensor modalities (e.g., inertial measurement units, RGB and RGB-D imaging systems, thermal cameras, GNSS tracking devices, and transcriptomic sequencing platforms), sampling frequencies, labeling protocols, and preprocessing pipelines. As reflected in the wide dispersion of reported performance metrics (Sect. "Summary of reported performance metrics"), predictive variability is closely associated with differences in data acquisition quality, annotation consistency, and environmental stability.

Hardware-related constraints further influence deployment feasibility. Wearable systems frequently involve trade-offs between sampling frequency and battery longevity, with reduced sampling rates negatively affecting classification robustness in behavior monitoring applications. In addition, the limited availability of large-scale, publicly accessible, and uniformly annotated datasets for sheep and goats constrains deep learning generalization and cross-study comparability. Although isolated dataset-sharing initiatives have improved transparency and reproducibility, systematic data standardization remains limited. A curated list of publicly available datasets relevant to AI research in small ruminants (*n* = 24) is provided in Supplementary Table S7.

#### Methodological limitations

Methodological heterogeneity represents a second systemic barrier. As summarized in Table 1, evaluation metrics varied considerably not only in magnitude but also in reporting consistency. Fewer than half of the included studies validated models on fully independent external datasets (Figure S10), restricting inference regarding cross-farm or cross-breed transferability.

As already noted (Sect. "Data synthesis"), the narrative rather than pooled nature of this synthesis means the mean values reported within each domain should be interpreted strictly as descriptive central tendencies, not as pooled estimates with inferential validity. Furthermore, the inclusion of studies published between September 2025, and December 2025 depended on a supplementary targeted search conducted in March 2026, which may not have achieved equivalent sensitivity to the primary search.

Non-English publications were excluded to maintain analytical consistency, which may have led to omission of relevant research from regions such as the Middle East, North Africa, and Asia where small ruminant production is highly significant. Additionally, frequent reliance on overall accuracy as the primary performance indicator was observed, even in contexts characterized by class imbalance (e.g., rare disease events or abnormal behavioral states). In such scenarios, accuracy alone may inadequately reflect minority-class detection performance. Studies reporting complementary metrics–such as precision, recall, F1-score, Matthews correlation coefficient (MCC), R^2^, or mean absolute percentage error (MAPE)–provided more comprehensive evaluation frameworks. While certain investigations employed synthetic data augmentation, multimodal fusion, or imbalance mitigation techniques, these methodological enhancements were not uniformly implemented across domains.

Collectively, the methodological landscape suggests that some high-performance reports may reflect optimized experimental configurations rather than externally validated, deployment-ready systems.

#### Socioeconomic and structural barriers

Beyond algorithmic performance, adoption feasibility is shaped by economic and structural factors. High initial costs associated with sensors, imaging infrastructure, computational hardware, and data management systems may limit accessibility, particularly in smallholder or extensive production systems. This contributes to uneven technological diffusion across production scales.

Environmental compatibility also influences scalability. Vision-based and sensor-intensive systems are frequently validated under controlled or semi-controlled conditions, whereas extensive grazing environments introduce variability in illumination, terrain, animal dispersion, and network connectivity, complicating stable deployment.

Model interpretability further affects stakeholder acceptance. Black-box deep learning architectures may limit user confidence in automated decision-support systems. Although explainable AI (XAI) approaches have been introduced in selected studies, interpretability frameworks remain inconsistently integrated across applications. The absence of explainability is particularly problematic for health-related applications, where automated disease alerts must be actionable and trusted by farm managers and veterinarians. Decision thresholds in diagnostic systems should be calibrated to reflect the asymmetric cost of error types: in disease detection, a false negative (missed case) typically carries greater welfare and economic consequences than a false positive (unnecessary intervention), and sensitivity should therefore be weighted more heavily than overall accuracy. Risk of bias in AI studies warrants explicit discussion. Overfitting is a significant concern in studies with small datasets and highly parameterized deep learning models, particularly when performance is evaluated only on internal cross-validation rather than truly independent external test sets. For example, several identification studies in this review achieved accuracies exceeding 99% on internal test sets derived from the same video sessions as the training data; performance on genuinely unseen animals would likely be substantially lower. Data leakage represents a further risk when images or sensor records from the same individual animal contribute to both training and test partitions, artificially inflating accuracy estimates. Non-independent cross-validation (e.g., k-fold without individual-level stratification) is particularly susceptible to this bias. Fewer than 50% of included studies employed fully independent external validation sets, limiting inference regarding cross-farm or cross-breed transferability. For health-related applications specifically, false negatives (missed disease cases) carry greater welfare and economic cost than false positives; models should report sensitivity, specificity, and positive predictive value separately, with decision thresholds calibrated to the asymmetric clinical cost of error types.

#### Synthesis

Across domains, the reviewed evidence indicates that predictive accuracy alone does not determine practical viability. Scalability depends on coordinated advances in data standardization, external validation practices, infrastructure compatibility, economic accessibility, and interpretability. The interaction of these technical, methodological, and structural dimensions shapes the transition from experimental proof-of-concept systems to operational tools within diverse small ruminant production contexts.

## Discussion

### Principal findings and thematic synthesis

This systematic narrative review synthesizes 92 original studies published between 2020 and 2025 that apply AI to sheep and goat production systems. As detailed in Sect. "Study selection and literature overview", the publication landscape has expanded rapidly, with annual output growing from 4 studies in 2020 to a peak of 26 studies in 2025. The field reached 52.2% of total output by the end of 2023, exceeding the 50% threshold, and 2025 was the most productive year (26 studies, 28.3%). While publication volume has accelerated, the distribution of research effort across application domains remains imbalanced, and translational maturity varies considerably among thematic clusters.

Two domains dominate the literature: Behavior and Activity Recognition (26.1%, *n* = 24) and Individual Animal Identification (19.6%, *n* = 18). This concentration reflects the foundational role of automated monitoring and individual identification in precision livestock systems, where scalable behavioral monitoring provides a functional entry point for digital management in small ruminants. As noted by Guo et al. [5], behavioral patterns serve as integrative indicators of physiological and health status. Similarly, Yuan et al. [49] emphasized the importance of reliable individual recognition for precision interventions at the animal level.

The species distribution (Figure S11) shows that sheep are studied more frequently than goats across all six research domains. This imbalance likely reflects the larger global sheep population, greater commercial investment in sheep production systems, and the relatively higher economic impact of sheep-borne diseases. Future research should address this gap to ensure that AI-driven innovations benefit both species equitably.

Performance metrics within these domains were comparatively high. Mean accuracies reached 92.4% for behavior recognition and 97.3% for identification tasks, with several studies reporting values exceeding 97%. Architectures based on convolutional neural networks, YOLO variants, and Vision Transformers were predominant. Notably, Jin et al. [13] reported 99.3% accuracy using multi-sensor fusion for behavior classification, while Zhang et al. [16] achieved 97.9% accuracy in facial recognition using an optimized transformer-based model. However, these high performances were typically observed under controlled or semi-controlled conditions, often with curated datasets and stable environmental parameters. External validation across diverse breeds, climatic regions, and management systems remains limited.

These two leading domains consistently achieved the highest mean accuracies across the included studies, reflecting a research trajectory that has prioritised scalable monitoring infrastructure over predictive health analytics.

A key cross-domain finding is the substantial variability in reported performance metrics. As summarized in Sect. "Summary of reported performance metrics", accuracy values ranged widely, particularly in biologically complex tasks such as disease detection. This heterogeneity reflects differences in data modality (sensor-based, image-based, molecular), environmental control (indoor vs. extensive grazing systems), and task definition (binary classification vs. multi-class recognition vs. regression). High accuracies were most consistently observed in structured classification tasks using high-quality, multimodal datasets in stable environments, whereas reduced performance characterized indirect inference tasks or applications implemented under heterogeneous field conditions.

Taken together, these findings indicate that the current AI landscape in small ruminant production is characterized by strong technical maturity in foundational monitoring and identification tasks, contrasted with emerging but less mature applications in health prediction, genomic modeling, and resilience analytics. The gap between controlled experimental validation and heterogeneous real-world farm environments represents a central translational challenge and frames the need for future research directions discussed below.

### Behavior and activity recognition: from sensing to understanding

The prominence of behavior and activity recognition within the reviewed literature reflects a broader conceptual shift: animal behavior is increasingly treated not merely as observable movement patterns, but as an integrative proxy for physiological state, welfare status, and reproductive dynamics. Applications range from generic activity classification to targeted detection of high-impact biological events, including lambing and estrus-related mounting behavior [6, 14, 15].

A central methodological development is the integration of multi-modal sensing and data fusion strategies. Rather than relying on single accelerometer streams, recent studies combine inertial measurement units (IMUs), positional data, and environmental inputs to capture richer behavioral signatures. For example, Jin et al. [13] demonstrated that fusing neck- and leg-mounted IMUs with positional information enabled classification of six grazing behaviors with 99.3% accuracy, even at a reduced sampling rate of 1 Hz. This finding is particularly relevant for field deployment, as it addresses the trade-off between data granularity and device autonomy.

Similarly, vision-based systems increasingly employ layered architectures that integrate object detection, pose estimation, and spatio-temporal modeling. Zhang et al. [67] combined YOLOv5-based detection, AlphaPose for pose estimation, and spatio-temporal graph convolutional networks (ST-GCN) to capture dynamic posture transitions, illustrating how computer vision frameworks can move beyond static detection toward structured behavioral interpretation. Gu et al. [46] further advanced this paradigm with a two-stage recognition method achieving > 98% accuracy for six behaviors, demonstrating that detection followed by classification can maintain high performance with model sizes below 130 MB.

Another defining trend is the prioritization of computational efficiency and edge deployability. Lightweight architectures such as FESS-YOLOv8n Guo et al. [5] achieved improved detection accuracy while reducing model size, enabling potential deployment on resource-constrained hardware. Decision-tree–based models optimized for microcontrollers [35] achieved > 91% global accuracy using only 10 sensor features, demonstrating that parsimonious models can be highly effective. Teacher-to-student information recovery strategies for low-frequency sampling [68] further illustrate how algorithmic design is increasingly shaped by hardware and energy constraints, achieving 3.33–7.6% percentage-point improvements at reduced sampling rates. These developments indicate a shift from purely performance-driven optimization toward deployment-aware model design.

The detection of biologically critical events represents one of the most impactful application areas. Lambing detection models have achieved high Matthews Correlation Coefficients under optimized conditions [14], while multi-stream systems integrating GNSS, accelerometry, and weather data have demonstrated detection rates up to 91% in grazing systems [7]. Estrus-related mounting detection under occlusion and variable illumination has also benefited from architectural enhancements incorporating spatial attention and multi-scale feature fusion [15], achieving 94.2% mAP with robust performance under low-light conditions. Sun et al. [47] extended this to periparturient behavior recognition, achieving 95.7% F1-score using 12-keypoint pose estimation.

Despite these advances, performance dispersion remains substantial. Reported accuracies range from 70.9% in extensive commercial settings [6] to near-perfect classification in tightly controlled experimental settings. This heterogeneity underscores the influence of environmental noise, breed variability, sensor placement, and annotation consistency on model robustness. Turner et al. [12] systematically addressed class imbalance using SMOTE augmentation, demonstrating that deep learning models (LSTM, BLSTM) significantly outperformed Random Forest (F1-score 0.84 vs. 0.65) when generalizing to unseen sheep. Bonneau et al. [11] similarly addressed imbalance through hierarchical LSTM architectures, achieving 87.84% F-score while noting that rare behaviors (0.42% of dataset) remained challenging.

One additional study meeting inclusion criteria (Esener & Eşki, 2025 [69]) was identified after the completion of data extraction and quantitative synthesis. It reports ensemble machine learning for early weight prediction in nomadic sheep flocks (R^2^ up to 0.86) and is consistent with the findings of the Growth domain, though it was not incorporated into the summary statistics in Table 1 due to post hoc identification.

Taken together, the evolution of behavior recognition in small ruminants reflects a progression from basic motion sensing toward structured behavioral inference supported by multi-modal integration and deployment-aware architectures. However, consolidation of the field will depend on standardized data collection frameworks, cross-site validation studies, and shared benchmark datasets capable of supporting reproducible and transferable model development.

### Identification and facial recognition: the quest for non-invasive biometrics

The strong representation of individual animal identification (19.6% of included studies) reflects a broader shift toward non-invasive biometric systems as alternatives to conventional tagging methods. Traditional identification approaches, including ear tags and physical markers, are susceptible to loss, injury, and manipulation, and may introduce welfare and labor constraints. In response, recent research has explored image-based, acoustic, and retinal biometric modalities as scalable digital solutions [49].

Across modalities, reported accuracies are consistently high, frequently exceeding 95%. However, interpretation of these values requires contextualization, as most studies were conducted under controlled acquisition conditions with limited breed diversity and relatively small population sizes.

Facial recognition has emerged as the dominant approach, largely due to its compatibility with standard RGB camera systems and its balance between accuracy and non-invasiveness. Yuan et al. [49] established a benchmark with Sheep FaceRec (97.14% accuracy), integrating MTCNN for detection and FaceNet for embedding extraction, maintaining 94.71% accuracy even at ≤ 30° face rotation. Transformer-based architectures have further enhanced feature representation capacity; Zhang et al. [16] reported 97.9% accuracy using a Vision Transformer framework (ViT-Sheep) with LayerScale module, illustrating the transferability of state-of-the-art computer vision models to livestock contexts. Lightweight variants designed for edge deployment, such as VLFaceNet (Li et al., 2024b) [19], demonstrate that inference speed (2.58 ms) and model compactness can be optimized without substantial performance degradation (97.75% accuracy). MobileViTFace [37] combining CNN and transformer structures achieved 97.13% accuracy with 5 × fewer parameters than ResNet-50, enabling real-time deployment on edge platforms.

Structured data acquisition pipelines generating large image datasets [70] highlight the importance of dataset scale and curation in achieving robust detection performance. Yadav et al. [71] demonstrated YOLOv8's superiority over VGG16 and CNN architectures across 35,204 images, achieving 95.8% accuracy. Huang et al. [36] reported exceptional performance (99.74% daytime, 99.89% nighttime) using DWT-GoatNet, which integrates wavelet transform with convolutional networks, outperforming classical CNNs.

Beyond facial recognition, alternative biometric modalities contribute complementary strengths. Acoustic identification approaches have demonstrated that vocalization patterns encode discriminative identity and emotional state information; Ntalampiras and Pesando [18] reported 95.8% accuracy while incorporating explainable AI techniques (Concept Relevance Propagation) to identify salient time–frequency features. Retinal imaging represents the highest-accuracy modality reported in the review, with Mustafi et al. [17] achieving 99% accuracy using stable vascular patterns around the optic disc. However, retinal systems require specialized equipment and controlled animal positioning, potentially limiting scalability despite their tamper-resistant properties.

The coexistence of multiple high-performing modalities suggests that identification performance is less constrained by algorithmic capability than by acquisition conditions, infrastructure availability, and deployment context. Facial recognition may offer the most practical balance for routine farm management, whereas acoustic or retinal systems may serve niche applications requiring enhanced robustness or security.

Nevertheless, external validation across heterogeneous production systems remains limited. Many models are trained on farm-specific datasets, raising questions regarding cross-breed generalizability, age-related morphological variation, and long-term robustness under changing environmental conditions. Li et al. [19] addressed open-set, multi-view recognition, achieving 96.13% accuracy across varying facial angles, representing progress toward more realistic deployment scenarios. Sun et al. [47] developed LAD-RCNN for livestock face detection and normalization, handling arbitrary face directions with rotation angle deviation < 6.42°, addressing the challenge of uncooperative animals.

As with behavior recognition, standardization of data acquisition protocols and cross-site benchmarking will be critical for transitioning biometric identification systems from high-accuracy experimental prototypes to interoperable components of integrated precision livestock platforms.

### Health, welfare, and disease detection: performance variability and translational challenges

Although comprising 19.6% of the included studies (*n* = 18), AI applications in health, welfare, and disease detection span multiple biological and management scales, from individual-level behavioral monitoring to molecular diagnostics and population-level risk modeling. This multi-scale distribution distinguishes the domain conceptually from behavior recognition or identification, as it attempts to infer latent physiological states rather than observable structural features.

At the individual animal level, sensor-based approaches aim to detect subtle behavioral deviations associated with pathology. Kaler et al. [10] demonstrated that ear-mounted accelerometers and gyroscopes could distinguish lame from non-lame sheep with accuracies exceeding 80% (84.91% for lying, 81.15% for standing, 76.83% for walking), revealing quantifiable movement asymmetries that are difficult to detect visually. Similarly, Fogarty et al. [8] explored accelerometer-derived activity patterns as indicators of *Haemonchus contortus* infection, identifying statistically significant–though modest–associations with fecal egg counts and packed cell volume. These findings illustrate the capacity of AI to detect behaviorally mediated physiological perturbations; however, the indirect nature of such proxies limits immediate diagnostic certainty.

At the field and farm level, deployable diagnostic systems have emerged with practical implications. Bucki et al. [9] validated a smartphone-based machine learning platform for on-farm parasite egg quantification, reporting performance non-inferior to accredited laboratory methods. This approach supports targeted selective treatment strategies and may contribute to mitigating anthelmintic resistance. Likewise, de Souza et al. [53] developed a mobile image-based screening tool for haemonchosis detection, achieving 87% accuracy with SVM classifiers under field conditions. These studies demonstrate that AI can bridge laboratory diagnostics and practical decision support, though sustained validation across diverse production systems remains limited.

At the molecular and cellular scale, AI models have been applied to high-dimensional omics data. Farr et al. [38] reported 99% classification accuracy using circulating miRNA profiles to distinguish pre- and post-procedural pain states in lambs, identifying a five-miRNA signature that classified castrated and tail-docked sheep from pre-treatment with remarkable precision. This demonstrates the potential for objective welfare assessment through molecular biomarkers. Sananmuang et al. [41] employed a hybrid 1D-CNN–GRU architecture to classify granulosa cell fertility potential in goats based on transcriptomic signatures, achieving 98.89% accuracy and identifying higher proportions of fertility-supporting cells in polytocous versus monotocous samples. These investigations highlight the analytical power of AI in extracting structured patterns from complex biological datasets. Nevertheless, such approaches are currently confined to controlled experimental contexts, and their integration into routine farm management remains uncertain due to cost, infrastructure, and sampling constraints.

At the population and systems level, predictive modeling extends toward herd benchmarking and epidemiological risk mapping. Kiouvrekis et al. [39] applied multiple ML algorithms to predict subclinical mastitis prevalence, achieving 96.3% accuracy for prevalence level classification (< 25% vs. ≥ 25%) using SVM. Lianou et al. [55] modeled bulk-tank milk quality in sheep and goat farms, with k-NN achieving the lowest MAPE (3.95%) for protein content prediction. At a broader spatial scale, Assefa et al. [40] developed an ensemble ecological niche model to map peste des petits ruminants virus risk under current and projected environmental conditions, with ensemble performance metrics (Kappa = 0.82, TSS = 0.88, ROC = 0.99) illustrating the role of AI in strategic disease surveillance. Eltahir et al. [72] demonstrated operational impact through a mathematical algorithm for smart vaccination against PPR, increasing coverage to 86% while reducing campaign duration from 70 to 30 days.

Despite this conceptual breadth, performance variability in the health and welfare domain remains substantial, with reported accuracies ranging from 62% [52] to 99% [38] and a mean (89.7%) lower than that observed in behavior or identification domains. This dispersion reflects the intrinsic complexity of disease phenotypes, multifactorial environmental interactions, variable data quality, and the relative scarcity of large, standardized datasets. Unlike identification tasks, which rely on relatively stable morphological features, health prediction often involves indirect inference from noisy or incomplete signals. Emsen et al. [66] achieved only 67.2% accuracy for maternal success prediction using Random Forest, highlighting that even well-executed ML may struggle with complex behavioral phenotypes.

Overall, AI applications in health and welfare demonstrate methodological diversity and multi-scale integration potential, yet translational maturity remains uneven. Progress toward clinically actionable systems will depend on larger multi-center datasets, harmonized annotation protocols, independent external validation, and integration of complementary modalities–including behavioral, physiological, and molecular data–within interpretable modeling frameworks.

The quality assessment (Supplementary Table S8, Part E) further reveals that the Health domain has the highest proportion of D3 = 0 studies (no data separation), which are exclusively Low quality, partly explaining the domain's higher performance variability. This underscores that validation rigor is a key determinant of study quality in health-related AI applications.

In disease detection contexts, false negatives (missed cases) typically carry substantially greater welfare and economic cost than false positives (unnecessary interventions). A model reporting 89% overall accuracy may achieve this figure by correctly classifying the abundant healthy class while misclassifying a substantial proportion of diseased animals – a critical failure mode for clinical applications. We therefore recommend that future health-monitoring studies report sensitivity, specificity, and positive predictive value separately, and define decision thresholds based on the clinical cost asymmetry of error types rather than relying on overall accuracy alone.

### Growth, weight, and body measurement: toward automated phenotyping frameworks

AI applications targeting growth, weight, and body measurement (9.8% of included studies, *n* = 9) address a central component of precision livestock systems: scalable, non-invasive phenotyping capable of supporting genetic selection, nutritional optimization, and market-oriented decision-making. Unlike identification tasks, which rely on relatively stable biometric features, automated phenotyping seeks to quantify dynamic traits influenced by age, management, and environmental variability. As such, this domain sits at the intersection of measurement technology and predictive analytics.

Image-based and 3D reconstruction systems represent one major technological pathway. Wei et al. [20] developed a multi-camera point cloud reconstruction framework using three KinectV2 devices, achieving average relative errors below 4% for morphometric traits including wither height (1.67%), chest girth (3.71%), and body length (3.57%). Importantly, the reduction in hardware complexity enhances feasibility relative to earlier, more sensor-intensive systems. However, the authors noted that irregular postures degrade measurement precision, highlighting a recurring limitation in vision-based phenotyping: morphological estimation remains sensitive to animal stance and movement.

Beyond direct morphometric extraction, machine learning regression models have been widely applied to predict growth traits from structured datasets. Esener and Kal [21] and Erduran et al. [22] employed ensemble tree-based approaches–including Random Forest, XGBoost, CatBoost, and ExtraTree–to predict weaning and yearling weights, reporting R^2^ values up to 0.81. A notable finding across these studies is the contribution of environmental covariates–such as weather parameters, elevation, and forage availability–to predictive performance. Erduran et al. [22] found that ExtraTree achieved the highest R^2^ (0.81) for live weight when incorporating month-9 records, while Esener and Kal [21] demonstrated that external data like weather and grassland features played significant roles. This reinforces the notion that growth traits are emergent outcomes shaped by animal–environment interactions, and that purely morphometric models may be insufficient without contextual integration.

At a more integrative level, multi-omics modeling expands phenotyping beyond observable traits. Alemu et al. [23] combined host genomic markers with rumen microbiome composition in a neural network–augmented GBLUP framework to predict methane emissions and residual feed intake. By incorporating principal components capturing 25–50% of microbial variation, the model improved prediction accuracy for methane from 0.09 to 0.30 and for RFI from 0.25 to 0.34, addressing complex trait architecture arising from host–microbiome interactions. While methodologically advanced, such frameworks currently require high-dimensional data infrastructures that may limit near-term scalability.

Deployment-oriented architectures have also been explored. He et al. [73] implemented a lightweight CNN (LiteHRNet) for weight estimation from RGB-D imagery, achieving moderate prediction error (MAPE 14.6%) with a compact parameter footprint (1.06M parameters), suggesting feasibility for edge computing environments. These efforts reflect an increasing awareness that phenotyping solutions must balance accuracy, hardware requirements, and operational practicality.

Despite encouraging technical performance, generalizability remains a central challenge. Many systems are validated on single breeds, limited age ranges, or controlled acquisition environments. Variability in posture, fleece characteristics, lighting conditions, and management systems can introduce systematic measurement bias. Moreover, cross-study benchmarking is constrained by differences in trait definitions, error metrics, and dataset scale. Vázquez-Martínez et al. [74] demonstrated that SVR and MARS algorithms could effectively predict body weight from biometric measurements, contributing to breed standard detection when weighing tools are unavailable. Haldar et al. [75] used RPART to identify heart girth and punch girth as most important for weight prediction in Indian Black Bengal goats, providing a practical tool for rural conditions.

Overall, automated phenotyping in small ruminants is transitioning from proof-of-concept image-based measurement toward integrative predictive modeling frameworks. Future progress will depend on harmonized validation protocols, pose-aware correction mechanisms, and multi-modal integration that combines morphometric, environmental, and genomic information within scalable and interpretable pipelines suitable for diverse production systems.

### Genomics, technical methods, and environmental applications: emerging but structurally intensive frontiers

Although genomics (8 studies) and technical, production, and environmental applications (15 studies) represent smaller proportions of the reviewed literature, they signal a structural expansion of AI beyond observational phenotyping toward high-dimensional biological modeling and ecosystem-level analytics. These domains differ fundamentally from behavior or identification tasks in that they rely on complex, often multi-layered data infrastructures and interdisciplinary integration.

At the genomic and molecular scale, AI has been applied to extract structured biological meaning from high-dimensional datasets. Sananmuang et al. [41] applied deep learning to single-cell transcriptomic data to classify granulosa cell fertility potential in goats with high reported accuracy. Similarly, Tang et al. [58] integrated RNA-seq analysis, weighted gene co-expression network analysis (WGCNA), and machine learning to investigate gene–environment interactions underlying hypoxia adaptation, identifying key regulatory genes (MAP3K5, TGFBR2, RSPO1, ITGB5) and pathways (HIF-1 signaling). These studies demonstrate the analytical capacity of AI to model complex regulatory networks and genotype–environment interplay.

In disease susceptibility, multi-omics integration has proven valuable. Chen et al. [59] combined microbiome, metabolome, and transcriptome data with Random Forest models to identify potential biomarkers (GlcADG, ethylmalonic acid, FBLIM1) associated with *Escherichia coli* F17 infection in sheep. Chen et al. [76] extended this to non-coding RNAs, using Random Forest and XGBoost to identify 44 circRNAs and 39 miRNAs as potential biomarkers, constructing ceRNA networks that provided insights into intestinal immunity mechanisms.

At the diagnostic level, Wang et al. [42] developed an artificial neural network from four hub genes (ADAM9, CRYL1, NDP52, SERPINA7) achieving 95.6% accuracy in diagnosing intrauterine growth restriction, with an IUGR scoring system attaining AUC 0.97. This demonstrates translational potential of AI-driven genomic biomarkers for clinical application.

For complex trait prediction, Alemu et al. [23] demonstrated that a neural network GBLUP framework demonstrated that integrating host genomic variation with rumen microbial composition improved prediction accuracy for methane emissions and residual feed intake [23], highlighting the value of incorporating microbial data into genomic prediction models. Zhang et al. [60] integrated genome and transcriptome data with neural network–based feature selection (Multilayer Perceptron with Boruta) to identify IRF2BP2 and EGFR as key functional genes underlying double-coated fleece formation in Hetian sheep, with the neural network model achieving high accuracy and AUC on independent test sets.

Technical and production-oriented applications illustrate additional methodological diversification. Ma et al. [61] leveraged foundation models–including ESM-2 protein embeddings and MolFormer chemical representations–to predict peptide transportability (accuracy 0.89) and taste-related properties (accuracy 0.99), outperforming traditional computational pipelines. Guevara et al. [45] applied machine learning regression (SMOreg) to dairy sheep lactation curve modeling, achieving correlation coefficients of 0.96 for total milk yield prediction, substantially outperforming traditional parametric models.

UAV-based computer vision systems further extend AI to remote livestock detection. Sarwar et al. [43] demonstrated that merging FCN with proposed CNN architectures increased recall from 90 to 98% for sheep detection in aerial images, enhancing system reliability. Zhao et al. [57] developed SheepInst, a high-performance instance segmentation model achieving Box AP 89.1% and Mask AP 91.3% for individual sheep extraction in high-density overlap scenarios, addressing challenges of irregular contours and occlusion. Wang et al. [63] extended this to satellite imagery with UGTransformer, achieving at least 1.8% improvement in mean IoU compared to established architectures for large-scale sheep extraction.

At the environmental scale, integration of animal movement data with remote sensing platforms represents a particularly interdisciplinary frontier. Paz-Kagan et al. [24] combined multi-season GPS tracking of goat herds with Landsat-8 satellite imagery and Random Forest modeling to evaluate grazing impacts on vegetation cover. By linking animal-level movement patterns to landscape-level vegetation dynamics, this approach exemplifies cross-scale modeling that connects livestock management with ecosystem processes. Stocking rate and distance to nearest corral emerged as primary predictors of vegetation change, demonstrating feasibility of linking management practices with ecosystem outcomes.

Collectively, these emerging domains highlight AI's capacity to bridge scales–from molecular pathways to landscape dynamics–and to integrate heterogeneous data modalities. However, they are also characterized by high data dimensionality, infrastructural demands, and limited cross-site validation. Compared with behavior recognition or biometric identification, which rely on relatively standardized sensor inputs, genomic and environmental AI systems require coordinated sampling strategies, interoperable databases, and interdisciplinary collaboration.

Thus, while these frontiers illustrate the conceptual breadth of AI in small ruminant systems, their translational trajectory will depend on developing scalable data ecosystems, reproducible pipelines, and cost-effective integration frameworks that connect laboratory-level analytics with field-level decision support. Their future impact may lie less in isolated high-accuracy demonstrations and more in their integration within multi-scale, interoperable precision livestock platforms.

### Implementation barriers and translational constraints

Despite measurable technical progress, translation to operational farm systems remains uneven. Barriers span data infrastructure, methodological rigor, economic feasibility, and institutional trust – collectively explaining the gap between high reported accuracies under controlled conditions and limited real-world deployment. Supplementary Table S7 provides publicly available datasets to support future research.

#### Data and infrastructure fragmentation

At the data level, heterogeneity is pervasive. Studies differ substantially in sensor types, sampling frequencies, annotation protocols, and environmental contexts, producing datasets that are difficult to harmonize or aggregate. As noted by Hu et al. [77], such fragmentation creates structural challenges for multi-modal integration and cross-study benchmarking. This variability contributes directly to the dispersion of reported performance metrics summarized in Sect. "Summary of reported performance metrics".

Moreover, publicly available, large-scale, well-annotated datasets for sheep and goats remain scarce relative to other livestock sectors. While open-access initiatives–such as those described by Billah et al. [78] and Mauny et al. [48]–represent positive steps, community-level standardization efforts remain limited. Without shared benchmark datasets and harmonized annotation frameworks, reproducibility and cross-site validation will remain constrained. Vayssade and Bonneau [79] addressed this through open-source tracking solutions ("Puzzle"), fostering open access, but such initiatives remain exceptions rather than the norm.

#### Methodological inconsistencies

Methodological variability further compounds translational challenges. A substantial proportion of studies rely on single-farm datasets and do not report validation on fully independent cohorts. This raises concerns regarding overfitting and limits inference about generalizability across breeds, climates, and management systems. Bonneau et al. [11] explicitly validated on unseen goats to ensure generalizability, but such rigor is not yet standard practice.

Additionally, reliance on aggregate metrics such as overall accuracy–without complementary reporting of precision, recall, F1-score, or specificity–reduces interpretability, particularly in imbalanced datasets where rare but critical events (e.g., disease onset) are underrepresented. Turner et al. [12] systematically addressed class imbalance and reported comprehensive metrics, demonstrating that BLSTM (F1-score 0.84) significantly outperformed Random Forest (0.65) on unseen sheep generalization.

More rigorous evaluation frameworks are demonstrably feasible. Sananmuang et al. [41] reported comprehensive performance metrics (precision, recall, F1-score) for molecular classification tasks, while Bonneau et al. [11] addressed class imbalance through hierarchical architectures and data augmentation. These examples indicate that methodological robustness is achievable but not yet consistently adopted.

The relationship between validation strategy and study quality is clearly illustrated in Figure S5: studies employing independent hold-out or cross-farm validation (D3 = 2) are predominantly High quality (77.8%), whereas studies with no data separation (D3 = 0) are exclusively Low quality. The distribution of validation strategies across all studies (Figure S10) shows that internal test sets are the most common approach (~ 70%), while true field deployment remains rare (~ 11%).

The domain-level analysis (Figure S4) reveals that D6 (Experimental Design and Confound Control) is the weakest domain across all research groups (grand mean 0.98), indicating that controlling for confounding variables—such as environmental fluctuations, breed differences, or management practices—remains a systematic challenge. This gap is particularly pronounced in health and behavior studies conducted under real-world conditions, where uncontrolled factors may substantially influence model performance.

#### Socioeconomic and adoption constraints

Even when technical feasibility is established, adoption depends on economic and institutional conditions. Sensor networks, imaging systems, and data management infrastructure require upfront capital investment and ongoing technical maintenance. Such requirements may be prohibitive for smallholder or resource-limited producers, potentially widening existing structural disparities in technology access.

Low-cost and edge-computing solutions offer partial mitigation. Smartphone-based parasite diagnostics [53] and microcontroller-optimized monitoring systems [35] illustrate pathways toward more accessible deployment models. Lightweight neural networks optimized for edge deployment [50] achieving high accuracy (91.7%) with minimal parameters demonstrate technical feasibility, but adoption pathways remain underdeveloped.

Environmental compatibility also influences scalability. Vision-based and sensor-intensive systems are frequently validated under controlled or semi-controlled conditions, whereas extensive grazing environments introduce variability in illumination, terrain, animal dispersion, and network connectivity, complicating stable deployment. Hou et al. [80] developed BiAF-YOLOv7 for dynamic goat herd detection and tracking, demonstrating applicability in real-world farming environments, but such field-validated systems remain relatively uncommon.

Model interpretability further affects stakeholder acceptance. Black-box deep learning architectures may limit user confidence in automated decision-support systems. Although explainable AI (XAI) approaches have been introduced in selected studies [18], interpretability frameworks remain inconsistently integrated across applications. Zhang et al. [81] integrated multi-dimensional feature fusion for sheep facial recognition in complex environments, but explicit XAI integration remains nascent.

#### Structural synthesis

Collectively, these barriers suggest that the principal limitation of AI in small ruminant systems is no longer algorithmic capability, but systemic integration. High-accuracy experimental demonstrations do not automatically translate into interoperable, scalable, and economically viable solutions. Bridging this gap will depend on coordinated data standardization, external validation across heterogeneous production systems, cost-aware hardware design, and participatory engagement with producers.

Thus, the transition from prototype to practice should be understood not as a purely technological progression, but as a systems-level transformation requiring alignment among data ecosystems, methodological standards, economic feasibility, and institutional trust.

### Limitations of this review

While this review provides a structured synthesis of AI applications in sheep and goat production systems, several limitations should be acknowledged.

First, the restriction to English-language publications may have introduced language bias, potentially excluding relevant studies published in other languages. Given the geographic diversity of small ruminant production systems, this constraint may have limited representation of region-specific innovations.

Second, the search strategy was confined to three major bibliographic databases–Web of Science, Scopus, and PubMed. Although these platforms index a substantial proportion of peer-reviewed literature in animal science and applied AI, relevant studies indexed in regional databases, conference proceedings, or gray literature sources may not have been captured. Despite a systematic search across three databases and a supplementary update, a small number of eligible studies published in late 2025 may have been inadvertently omitted due to the lag between online publication and database indexing (e.g., Esener & Eşki, 2025 [69]). Such omissions are a recognized limitation of any systematic review and are acknowledged transparently.

Third, the review protocol was registered with the Open Science Framework after completion of the initial literature searches but before screening, data extraction, and final synthesis. Although this registration was transparently reported as partially prospective, retrospective registration carries a potential risk that methodological decisions could be influenced by preliminary knowledge of the retrieved literature. To minimize this risk, predefined eligibility criteria, transparent screening procedures, and reproducible data extraction methods were applied and documented.

Fourth, the marked heterogeneity across the 92 included studies-in terms of experimental design, data sources, AI architectures, validation procedures, and performance metrics-precluded formal meta-analytic synthesis. As a result, comparative evaluation was conducted narratively rather than through pooled effect size estimation. This reflects not only a methodological limitation of the review but also the broader fragmentation within the field, as discussed in Sect. "Implementation barriers and translational constraints".

Fifth, thematic categorization of studies into application domains required structured yet interpretative judgment, particularly for multi-modal or cross-domain studies. Although classification criteria were applied consistently, alternative domain assignments might yield slightly different proportional distributions.

Sixth, the quality assessment framework used in this review was specifically developed for AI-based livestock studies because no universally accepted appraisal tool currently exists for this emerging research area. Although the framework demonstrated reproducibility and enabled structured comparison across studies, it remains an author-developed instrument that has not undergone external validation. Therefore, quality scores should be interpreted as supportive indicators of methodological robustness rather than definitive measures of study quality.

Finally, given the rapid pace of methodological development in artificial intelligence, particularly in foundation models and multi-modal learning, some very recent advances may not be fully represented despite the search being updated in March 2026 to capture late 2025 publications The field’s accelerated evolution implies that periodic updates will be necessary to maintain an accurate synthesis. Our eligibility criteria, which limited inclusion to peer-reviewed original research articles and English language publications, may have excluded some relevant review articles, short communications, conference proceedings, or non-English publications. This is an inherent limitation of systematic reviews in rapidly evolving fields.

Taken together, these limitations reflect both constraints inherent to systematic reviews in rapidly evolving technological domains and structural characteristics of the current literature. Continued standardization in reporting, broader data sharing, and harmonized benchmarking frameworks will enhance the precision and comparability of future evidence syntheses in AI-driven small ruminant research.

### Practical directions for future research

Advancing AI applications in sheep and goat production systems requires coordinated progress across data infrastructure, methodological rigor, and domain-specific integration. Rather than incremental performance improvements within isolated tasks, future research will benefit from structurally aligned strategies that enhance robustness, generalizability, and translational readiness.

In behavior and activity recognition–the most methodologically mature domain–the next phase of development lies in harmonization. The establishment of synchronized, multi-modal datasets integrating wearable inertial measurement units (IMUs), positional tracking, and 3D vision systems would facilitate cross-study comparability and external validation. Shared benchmark datasets and standardized annotation frameworks would enable reproducible evaluation and reduce the performance variability currently observed across studies. Particular attention should be paid to rare-event detection (e.g., lambing, mounting), where class imbalance remains challenging; synthetic data augmentation and hierarchical architectures represent promising methodological pathways.

Within identification and facial recognition, priority should shift from optimizing accuracy under controlled conditions to evaluating robustness across heterogeneous production environments. Cross-breed, multi-age, and multi-farm validation studies are essential to assess real-world generalizability. Recent work by Sun et al. [47] demonstrated progress toward handling arbitrary face directions, but broader validation across breeds and environmental conditions remains needed. Additionally, multi-modal biometric integration–combining facial imagery with vocal or retinal data–may enhance system resilience under occlusion, lighting variation, or partial visibility, thereby moving from single-modality optimization toward fault-tolerant identification architectures. Lightweight, edge-deployable models [50, 51] should be prioritized for practical scalability.

For health, welfare, and disease detection, progress will depend on multi-scale dataset integration. Linking behavioral indicators, physiological measurements, molecular biomarkers, and environmental variables within unified modeling frameworks may improve predictive reliability for multifactorial conditions. The impressive performance of miRNA-based models [38] suggests potential for molecular diagnostics, but cost-effective, field-deployable implementations remain elusive. Rigorous validation protocols–including independent cohort testing and transparent reporting of multiple performance metrics (precision, recall, F1-score, specificity)–are critical for developing clinically actionable tools rather than proof-of-concept classifiers. Cost-aware and field-adapted implementations, such as smartphone-based diagnostics [53], remain particularly important for ensuring accessibility across diverse production contexts.

In automated growth and body measurement, combining 3D morphometric extraction with pose-aware correction mechanisms can mitigate posture-related measurement bias [20]. Integrating environmental, nutritional, and management covariates within predictive models may further improve phenotypic interpretation, reinforcing the transition from static measurement toward context-aware phenotyping pipelines. The contribution of environmental factors to growth variability [21] underscores the need for models that incorporate contextual information rather than relying solely on biometric inputs. Lightweight, low-cost imaging solutions [56, 73] should be prioritized for routine farm deployment.

Genomics and molecular applications represent a high-dimensional frontier where integration is central. Expanding multi-omics datasets across breeds and environments, coupled with machine learning architectures capable of modeling genotype–environment interactions, may strengthen the relevance of AI-driven selection strategies. The integration of host genomic variation with microbial composition [23] illustrates the potential of cross-kingdom modeling for complex trait prediction. However, ensuring reproducibility and interpretability in these data-intensive systems will be essential for their incorporation into breeding programs. Establishing standardized pipelines for omics data processing and model validation should be prioritized.

Environmental and technical applications would benefit from long-term, landscape-scale modeling that integrates animal movement data with remote sensing and climatic indicators. Paz-Kagan et al. [24] demonstrated the feasibility of linking grazing management to vegetation dynamics, but such frameworks require interoperable spatial data infrastructures and sustained monitoring efforts. UAV-based monitoring [43] and satellite-based extraction [63] offer complementary scales of observation; integrating these modalities could provide multi-resolution assessment of livestock-environment interactions. Decision support systems that translate AI outputs into actionable management recommendations [64] should be developed and validated in partnership with producers.

Across all domains, four cross-cutting priorities emerge as essential for translating experimental advances into operational impact. First, the development of standardized data ecosystems constitutes a necessary precondition for reproducible progress. This requires publicly available, well-annotated benchmark datasets governed by consistent acquisition protocols and harmonized metadata standards–an infrastructure currently lacking in small ruminant research. Second, external validation must become standard practice, with systematic evaluation across farms, breeds, and environmental conditions to establish genuine generalizability rather than context-specific overfitting. Third, a shift toward deployment-aware design is imperative: models must be optimized for resource-constrained edge devices, deliberately balancing predictive accuracy against computational efficiency, energy autonomy, and practical deployability within extensive production systems. Fourth and finally, the integration of explainable and trustworthy AI frameworks is critical to bridging the "black-box" trust deficit, particularly in health and welfare applications where clinical decisions carry high stakes and user confidence determines adoption. Collectively, these priorities reflect a maturation of the field–a recognition that sustained impact depends not on incremental accuracy gains, but on systemic alignment among data infrastructure, validation rigor, hardware feasibility, and interpretability.

Collectively, future progress will depend less on isolated algorithmic innovation and more on coordinated ecosystem development–encompassing standardized data practices, multi-modal integration, cross-site validation, and economically viable deployment models. Strengthening these structural foundations will accelerate the transition from experimental AI demonstrations to resilient, farm-integrated systems capable of enhancing productivity, welfare, and environmental stewardship in small ruminant production.

## Conclusions

This systematic narrative review synthesizes 92 peer-reviewed studies published between 2020 and 2025, providing the first structured, domain-spanning overview of AI applications in sheep and goat production systems. The evidence demonstrates strong proof-of-concept performance, particularly within behavior and activity recognition (26.1% of studies, mean accuracy 92.4%) and biometric identification (19.6% of studies, mean accuracy 97.3%). In these foundational domains, consistently high performance suggests that non-invasive monitoring and automated identification are approaching proof-of-concept maturity under controlled or semi-controlled conditions. However, the evidence base for health and welfare monitoring, genomic prediction, and extensive-field deployment is considerably less mature. Operational feasibility must therefore be qualified by application domain and deployment context. The transition to scalable, real-world impact requires: (i) standardized, publicly available annotated datasets representative of diverse breeds and management systems; (ii) rigorous independent cross-farm and cross-breed validation; (iii) transparent, domain-appropriate metric reporting that reflects the clinical or management significance of error types; and (iv) deployment-oriented evaluation under real-world rather than controlled conditions.

The overall landscape, however, remains uneven. Domains addressing health, growth, genomics, and environmental applications exhibit greater performance variability and less translational maturity, consistent with the domain-specific patterns detailed in Sects. "AI applications in health, welfare, and disease detection"–"AI applications in technical methods, production, and environment".

The next phase of development will depend on consolidating fragmented advances into interoperable, multi-modal frameworks. Standardized data protocols, shared benchmarking initiatives, independent external validation across heterogeneous production environments, and economically viable deployment models are critical to ensuring that high experimental accuracy translates into resilient, farm-integrated systems. The methodological diversity documented in this review–from lightweight edge-optimized architectures to multi-modal sensor fusion and explainable AI frameworks–provides a rich foundation for such progress. Moreover, alignment among researchers, technology developers, policymakers, and producers will be essential to bridge technical innovation with practical adoption, particularly in smallholder and extensive production contexts where accessibility and cost-effectiveness are paramount.

Ultimately, AI in small ruminant production should be viewed not merely as a collection of predictive tools, but as an emerging data infrastructure capable of linking animal-level phenotypes, molecular insights, management decisions, and environmental outcomes. Realizing this potential will require sustained methodological rigor, interdisciplinary collaboration, and attention to equitable access. The foundational evidence synthesized here demonstrates technical feasibility; the transformative impact will depend on how effectively the field advances from isolated experimental prototypes to coherent, scalable, and context-adaptive livestock intelligence systems capable of enhancing productivity, welfare, and environmental sustainability across diverse production landscapes.

## Supplementary Information

Supplementary Material 1.

## Data Availability

All data generated or analyzed during this study are included in this published article and its supplementary information files. The complete list of included studies is provided in the reference section. Supplementary Tables S1–S8 and Figures S1–S19 are available in the online version of the article. The study protocol is publicly available in the Open Science Framework (OSF) repository (https://doi.org/10.17605/OSF.IO/VFPQK). Python scripts used for data processing and analysis are available from the corresponding author upon reasonable request.

## Abbreviations

AI: Artificial Intelligence
ANN: Artificial Neural Network
AUC: Area Under the Curve
BLSTM: Bidirectional Long Short-Term Memory
CNN: Convolutional Neural Network
DL: Deep Learning
F1: F1-score
GBLUP: Genomic Best Linear Unbiased Prediction
GNSS: Global Navigation Satellite System
GPU: Graphics Processing Unit
GRU: Gated Recurrent Unit
IMU: Inertial Measurement Unit
k-NN: K-Nearest Neighbors
LSTM: Long Short-Term Memory
MAPE: Mean Absolute Percentage Error
mAP: Mean Average Precision
MCC: Matthews Correlation Coefficient
MeSH: Medical Subject Headings
ML: Machine Learning
MTCNN: Multi-task Cascaded Convolutional Network
OSF: Open Science Framework
PLF: Precision Livestock Farming
PPR: Peste des Petits Ruminants
PRISMA: Preferred Reporting Items for Systematic Reviews and Meta-Analyses
R^2^: Coefficient of Determination
RGB: Red, Green, Blue
RNA-seq: RNA sequencing
SNP: Single Nucleotide Polymorphism
SNR: Systematic Narrative Review
SVM: Support Vector Machine
UAV: Unmanned Aerial Vehicle
XAI: Explainable Artificial Intelligence
YOLO: You Only Look Once
