# Supplementary material for: A systematic review of artificial intelligence in small ruminant production systems: applications, performance outcomes, and reported implementation challenges
Source: BMC Vet Res. 2026 Aug 20;22:485. doi: 10.1186/s12917-026-05806-z (PMC13495206; doi:10.1186/s12917-026-05806-z)
Supplement: Supplementary file 1 [file 12917_2026_5806_MOESM1_ESM.pdf]

SUPPLEMENTARY MATERIALS

A Systematic Review of Artificial Intelligence in Small Ruminant Production Systems: Applications, Performance Outcomes, and Reported Implementation Challenges

The following supplementary materials accompany this article and provide detailed information on the 92 original research studies included in the systematic review. All tables and figures are organized by the six thematic domains identified during data extraction.

Table of Contents

Supplementary Tables (n = 8)

Seven thematic tables present the 92 primary studies; Table S8 provides the quality assessment instrument and all study-level ratings.

Reference	Title	Scope
Table S1	Studies on Behavior and Activity Recognition	n = 24
Table S2	Studies on Individual Animal Identification	n = 18
Table S3	Studies on Health, Welfare, and Disease Detection	n = 18
Table S4	Studies on Growth, Weight, and Body Measurement	n = 9
Table S5	Studies on Genomics and Molecular Biology	n = 8
Table S6	Studies on Technical Methods, Production, and Environment	n = 15
Table S7	Publicly Available Datasets Supporting AI Research	n = 24
Table S8	Quality Assessment Instrument and Study-Level Ratings	Parts A–F

Supplementary Figures (n = 19)

Nineteen figures support interpretation of the quantitative synthesis, covering quality distributions, geographic patterns, technology trends, publication timelines, and species comparisons. Figures are ordered by logical narrative flow: quality assessment → geography → performance → technology → applications → species.

Reference	Title	Key Information
Figure S1	Overall Study Quality Distribution — Donut Chart	Proportions across all 92 studies (High=27, Mod=60, Low=5)
Figure S2	Study Quality Distribution by Research Group — Absolute Counts	Stacked bar chart, absolute n per tier per group
Figure S3	Distribution of Composite Quality Scores — Histogram	Frequency histogram; mode at score 12 (n=36, 39.1%)
Figure S4	Mean Domain Scores (D1–D8) by Research Group — Heatmap	Color-coded heatmap; D6 weakest (mean 0.98)

Reference	Title	Key Information
Figure S5	Study Quality by Validation Strategy — D3 Score	Dual panel: absolute + normalized; monotonic D3 relationship
Figure S6	Distribution of Quality Scores by Research Group — Box Plot	IQR spreads; Growth & Body tightest; Behavior widest
Figure S7	Geographic Distribution — Studies by Country	Horizontal bar chart; China n=40 (43.5%)
Figure S8	Geographic Distribution by Continent	Donut chart; Asia 56.5%, Europe 21.7%, Oceania 12.0%
Figure S9	Performance Score Distribution by Research Group	Box plots of reported accuracy values per thematic group
Figure S10	Distribution of Validation Strategies	Donut chart; internal test set ~70%; field deployment ~11%
Figure S11	Species Distribution by Research Group	Grouped bar chart; sheep consistently outnumber goats
Figure S12	Top 10 Most Used AI/ML Technologies	CNN/DL n=20; YOLO n=14; Random Forest n=12; ANN n=11
Figure S13	Top 10 Most Common Applications	Live weight & detection n=5 each; mastitis & parasite n=4 each
Figure S14	Yearly Publication Trends with Linear Trendline (2020–2025)	Growth from 4 (2020) to 26 (2025); R^2=0.821
Figure S15	Types of Input Data Used in Studies	Camera/video n=13; facial images n=12; accelerometer n=8
Figure S16	Technology Adoption Timeline (2020–2025)	Multi-line chart; CNN/DL & YOLO show sharpest growth
Figure S17	Strength Patterns in High-Quality Studies	Robust validation n=12 (34%); lightweight arch. n=7 (20%)
Figure S18	Top 10 Best-Performing Studies	Bati & Ser 100%; Huang et al. 99.89%; ID group dominates
Figure S19	Country × Research Group Specialization Heatmap	China → Behavior; Greece → Health; Turkey → Production

Table S1: Prominent Studies in Behavior and Activity Recognition for Sheep and Goats (n=24)

Study (Author, Year)	Species	Country	AI Method / Architecture	Input Data / Modality	n Animals	Performance Metric & Value	Validation Strategy	Quality Tier
**Bati & Ser (2023)**	Sheep	Turkey	Optical Flow + CNN, ConvLSTM, VGG19	Video	100	Accuracy up to 100% (BOF-CNN on balanced datasets)	Internal test set	Moderate
**Bonneau et al. (2024)**	Goat	France	Hierarchical LSTM + data augmentation	Accelerometer (horn, 25 Hz)	59	F1: 87.84%; Precision: 89.44%; Recall: 86.3%	Independent unseen animals	High
**do Nascimento Amorim et al. (2025)**	Sheep	Brazil	Neural Networks	Electronic device	19	Accuracy: feeding 96.7%, rumination 89.6%, other 90.4%	Internal test set	Moderate
**Gu et al. (2023)**	Sheep	China	Two-stage CNN (YOLOv5-based detection + VGG16 classification)	Camera	Not specified	Detection mAP >98%; Classification accuracy >94%	Internal test set	Moderate
**Guo et al. (2025)**	Sheep	China	FESS-YOLOv8n (lightweight with EMA, SCSA, SCDown)	Camera (video)	1,200 videos (expanded to 4,979 images)	mAP@0.5: 91.4% (improved from 86.9% baseline)	Internal test set	Moderate
**Hollevoet et al. (2024)**	Goat	Belgium	MLP, CNN, hCNN, SVM, RF (comparative study)	Accelerometer (collar, 200 Hz)	4	hCNN + L2-norm: highest accuracy; LOOCV showed generalization gap	Mixed CV + goat-wise LOOCV	High
**Hu et al. (2020)**	Sheep	Australia	Random Forest, SVM, Linear Discriminant Analysis (LDA)	Accelerometer	17	Significant improvement with mixed window sizes (2-15s); RF best	Internal test set	Low
**Jiang et al. (2020)**	Goat	China	YOLOv4 + spatial location & centroid movement analysis	Video (experimental)	Not specified	Accuracy: eating 97.9%, drinking 98.3%, active 96.9%, inactive 96.9%	Internal test set	Moderate
**Jin et al. (2024)**	Sheep	China	CNN-LSTM, TCN-Transformer	IMU (neck + leg) + GPS	22	Accuracy: 99.3% (CNN-LSTM); >96% even at 1 Hz	Cross-validation + field test	High
**Mao et al. (2023)**	Sheep/Goat	China	Teacher-to-Student Information Recovery (T2S-IR)	Accelerometer (public horse & goat datasets)	Two public datasets	Improvement: +3.33–7.6% at low sampling rates	Internal test set	Moderate
**Méndez et al. (2025)**	Goat	Spain	ConvNeXt-V2, YOLOv8 + 3 other computer vision algorithms	Camera (RGB)	15	mAP: ConvNeXt-V2 0.95, YOLOv8 0.94	Internal test set	Moderate
**Nóbrega et al. (2020)**	Sheep	Portugal	Decision Trees (optimized for low-power microcontroller)	Accelerometer	Not specified	Global accuracy >91% using 10 sensor features	Internal + deployment test	High
**Price et al. (2022)**	Sheep	UK	Random Forest	Accelerometer (collar for ewes, harness for lambs)	Commercial flock (not specified)	Posture: ewes 83.7%, lambs 85.9%; Activity: ewes 70.9%, lambs 80.8%	LOOCV	Moderate
**Ser et al. (2025)**	Sheep	Turkey	CNN, ConvLSTM, VGG19 + dense optical flow	Video	116	ConvLSTM: 92% active sheep, 83% overall accuracy	Cross-validation	Moderate
**Sun et al. (2025a)**	Sheep	China	YOLOv8-BCD (lightweight with adaptive multi-scale feature aggregation)	Camera	1,476 annotated images	Accuracy: 91.7%; Speed: 389 FPS; Parameter reduction: 19.2%	Internal test set	Moderate
**Sun et al. (2025b)**	Sheep	China	Improved YOLOv8n-Pose + BP neural network	Camera	Self-built dataset (size not specified)	F1-score: 95.7%; Key-point mAP50 improved by 4.6%	Internal test set	Moderate

Study (Author, Year)	Species	Country	AI Method / Architecture	Input Data / Modality	n Animals	Performance Metric & Value	Validation Strategy	Quality Tier
**Suparwito et al. (2021)**	Sheep	Australia	Deep Belief Network (DBN)	Wearable sensor	Not specified	MSE: 4.46 (training), 20.65 (testing)	K-fold cross-validation	Low
**Turner et al. (2023)**	Sheep	Australia	LSTM, Bidirectional LSTM (BLSTM) + SMOTE	Accelerometer (jaw 30 Hz / ear 25 Hz)	19	BLSTM F1: 0.84 vs RF 0.65 (unseen sheep generalization)	Cross-farm unseen animal test	High
**Wang et al. (2021)**	Sheep	China	DNN, CNN, RNN with feature extraction	Acoustic signals	3 sheep; 21,924 labeled samples	RNN classification: 93.17%; ID algorithm: 96.13%	Internal test set	Moderate
**Xie et al. (2025)**	Goat	China	ACLNet (stereo matching network with attention + ConvGRU) + 2D pose model	Binocular stereo camera	Goat depth dataset	EPE: 0.56; MPJPE: 45.7 mm (improved by 21.1 mm)	Internal test set	Low
**Xu et al. (2023)**	Sheep	China	YOLOv5 + LSTM (spatio-temporal)	Video (surveillance camera, 25 fps)	Not specified (max 70 sheep/pen)	Precision: 93.38%; Recall: 91.86%	Train/test split (80:20)	High
**Yan et al. (2025)**	Sheep	China	SIDS-YOLOv11 (improved YOLOv11 with SCINet, iAFF, DySample, SEAM)	Camera (low-light)	24 ewes, 4,700 images	mAP@0.5: 0.942; Precision: 0.956; Recall: 0.854	Internal test set	Moderate
**Zhang et al. (2021)**	Sheep	China	BP Neural Network, DNN, CNN	Wireless sensor	Not mentioned (Mongolian sheep)	CNN 93.8% > DNN 89.4% > BP 83.1%	K-fold cross-validation	Moderate
**Zhang et al. (2024a)**	Sheep	China	YOLOv5 + Deep-SORT + Alphapose + ST-GCN	Camera	15	Qualitative (feasibility of the method demonstrated)	Proof of concept	Low

Table S2: Individual Animal Identification in Sheep and Goats (n=18)

Study (Author, Year)	Species	Country	AI Method / Architecture	Input Data / Modality	n Animals	Performance Metric & Value	Validation Strategy	Quality Tier
**Billah et al. (2022)**	Goat	New Zealand	CNN	Facial images	Two public datasets	Recognition accuracy: 96.4%; Detection AP: face 93%, eye 83%, nose 92%, ear 85%	Internal test set	Moderate
**Himel et al. (2024)**	Sheep	Not specified	Ensemble CNN (Xception, VGG16, InceptionV3, InceptionResNetV2, DenseNet121) + Random Forest (meta-learner)	Facial images	1,680 images (4 breeds)	Accuracy: 97.92%	Train/validation/test split (80:10:10)	High
**Hou et al. (2025)**	Goat	China	BiAF-YOLOv7 (improved YOLOv7-tiny with CBAM) + DeepSORT	Camera	Goat herd dataset	Precision: 94.5%, Recall: 96.7%, F1: 94.8%, mAP: 96.0%	Internal test set	Moderate
**Huang et al. (2023)**	Goat	China	DWT-GoatNet (2D-DWT + convolution)	Facial images	30 Saanen dairy goats	Accuracy: 99.74% (daytime), 99.89% (night)	Internal test set	High
**Li et al. (2023)**	Sheep	China	MobileViTFace (CNN + Transformer)	Facial images	186 sheep, 7,434 images	Accuracy: 97.13%; 5x fewer parameters than ResNet-50	Internal test set	High
**Li et al. (2024a)**	Sheep	China	SheepFaceNet (lightweight) + Li-ArcFace loss	Facial images (multi-view)	212 sheep, 3,801 images	Open-set accuracy: 96.13% (self-built), 93.33% (public)	Internal test set	High
**Li et al. (2024b)**	Sheep	China	VLFaceNet (VL modules + ECA attention) + SFE feature enhancement	Facial images	Self-built dataset	Accuracy: 97.75%; Inference latency: 2.58 ms	Internal test set	High
**Mustafi et al. (2021)**	Goat	India	Image segmentation + encoding + Hamming distance matching	Retinal images	12 goats, >200 images	Accuracy: 99%	Internal test set	High
**Ntalampiras & Pesando (2025)**	Goat	Italy	CNN + Concept Relevance Propagation (CRP) for XAI	Vocalizations (acoustic)	Public dataset (VOCAPRA)	Classification rate: 95.8%	Public dataset validation	High
**Pang et al. (2023)**	Sheep	China	Attention Residual Module (ARM) + VGG16, GoogLeNet, ResNet50	Facial images	38 sheep, 4,490 images	Accuracy improvement: +10.2% (VGG16), +6.65% (GoogLeNet), +4.38% (ResNet50)	Internal test set	Moderate
**Sun et al. (2023b)**	Goat	China	LAD-RCNN (Lightweight Angle Detection R-CNN)	Facial images (including infrared)	Goat dataset	Face detection AP >97%; Rotation angle deviation <6.42°; Speed: 13.7 ms/image	Internal test set	High
**Wang et al. (2023b)**	Goat	China	Contextual information + feature-fusion complementation network	Facial images (low-resolution)	8,871 images, 65,894 annotated faces	AP improvement: +8.07%; P: +0.06; R: +6.8%	Internal test set	Moderate
**Yadav et al. (2024)**	Sheep/Goat	Brazil	YOLOv8, VGG16, CNN (comparison)	Physical characteristics / images	35,204 images	YOLOv8 accuracy: 95.8% (best performer)	Systematic cross-architecture comparison	High
**Yuan et al. (2025)**	Sheep	China	MTCNN (detection) + FaceNet (feature extraction)	Facial images	564 sheep (+50 for generalization)	Accuracy: 97.14% (test), 95.88% (generalization); 94.71% at ≤30° rotation	Internal test set + generalization set	High

Study (Author, Year)	Species	Country	AI Method / Architecture	Input Data / Modality	n Animals	Performance Metric & Value	Validation Strategy	Quality Tier
**Zhang et al. (2022)**	Goat	China	Improved YOLOv5 + RandAugment + AF-FPN + Dynamic Head + DeepSORT	Camera	623 images; video from multiple pens	Detection mAP: 92.19%; Tracking overlap: 89.69%	Internal test set	Moderate
**Zhang et al. (2023)**	Sheep	China	Vision Transformer (ViT) + LayerScale + transfer learning	Facial images	160 sheep	Recognition accuracy: 97.9%	Internal test set	High
**Zhang et al. (2024b)**	Sheep	China	YOLOv7-SFR (shuffle attention + Dyhead + depthwise conv + knowledge distillation)	Facial images	50 sheep, 22,000 images	mAP@0.5: 96.9%; Model size: 11.3 MB; Recognition time: 3.6 ms	Internal test set	High
**Zhang et al. (2025a)**	Sheep	China	Improved YOLOv5s + GhostNet (SSSA) + EfficientNet	Camera	30 adult sheep	mAP counting: 95.84%; Facial ID: 98.9%; Expression: 99.2%	Internal test set	Moderate

Table S3: Prominent Studies in Health, Welfare, and Disease Detection for Sheep and Goats (n=18)

Study (Author, Year)	Species	Country	AI Method / Architecture	Input Data / Modality	n Animals / Samples	Performance Metric & Value	Validation Strategy	Quality Tier
**Assefa et al. (2021)**	Sheep/goat (general)	Ethiopia	Ensemble ecological niche modelling (9 models)	Environmental / spatial data	Global occurrence data	Kappa: 0.82; TSS: 0.88; ROC: 0.99	Spatial cross-validation	High
**Betancor-Sánchez et al. (2025)**	Goat	Spain	Decision Trees, Neural Networks	Colostrum color data	813 colostrum samples	Decision tree outperformed neural networks (matching ELISA)	Internal test set	Moderate
**Bucki et al. (2023)**	Sheep	Ireland	Machine Learning (video analysis)	Smartphone microscopy	105 ovine faecal samples	Non-inferior to accredited laboratory	Comparative lab validation	Moderate
**de Souza et al. (2023)**	Sheep	Brazil	Neural Network, SVM	Smartphone (ocular conjunctival images)	317 images from 5 herds	SVM: 87%; Neural Network: 83%	Internal test set	Moderate
**Emsen et al. (2025)**	Sheep	Turkey	Random Forest, Decision Trees, Logistic Regression, SVM	Behavioral / maternal scoring	Native and crossbred prolific ewes	Random Forest accuracy: 67.2%	Internal test set	Low
**Farr et al. (2025)**	Sheep	Australia	Supervised ML (SVC, LR, DT, SGD, RF) – 5-miRNA signature	Circulating miRNA levels	42 samples (pre- and post-castration)	Classification accuracy: 99%	Internal test set	High
**Fogarty et al. (2021)**	Sheep	New Zealand	Random Forest	Accelerometer + GPS + weather	Two field trials	Detection of up to 91% of lambing events	Internal test set	Moderate
**Fogarty et al. (2023)**	Sheep	Australia	Machine Learning (activity classification)	Accelerometer	45 sheep	Weak correlation (FEC/PCV) – qualitative	Internal test set	Low
**Gurule et al. (2021)**	Sheep	USA	Random Forest	Accelerometer	13 ewes	7 behaviors: 66.7%; activity: 87.2%	Internal test set	Low
**Kaler et al. (2020)**	Sheep	UK	Random Forest	Accelerometer	23 datasets (10 non-lame, 13 lame)	Lying: 84.91%; Standing: 81.15%; Walking: 76.83%	Internal test set	Moderate
**Kiouvrekis et al. (2024)**	Sheep	Greece	K-means, Decision Trees, k-NN, Neural Networks, SVM	Farm records / milk data	111 farms (training), 373 records (evaluation)	Overall: 94.1%; Prevalence level: 96.3%	Internal test set	High
**Lianou et al. (2024)**	Sheep/Goat	Greece	Decision Trees, Random Forest, XGBoost, k-NN, Neural Networks	Bulk-tank milk quality data	325 sheep farms, 119 goat farms	MAPE: k-NN 3.95% (protein) – no fat prediction	Internal test set	Moderate
**Lysitsas et al. (2024)**	Sheep	Greece	YOLOv8 (udder ROI detection)	Infrared thermography	330 ewes, 660 milk samples, >2000 thermal images	YOLOv8 ROI accuracy: 84% (preliminary)	Internal test set	Moderate
**Martone et al. (2025)**	Sheep	Italy	Classification Tree, Random Forest	Parasitological + farmer perception	138 sheep farms	Identified key predictors (treatment frequency, method, awareness)	Internal test set	Low
**Siddique et al. (2024)**	Goat	USA	SVM, KNN, BPNN, Keras deep learning	Blood pattern images on glycerol-soaked paper	75 goats; 1,054 images	Accuracy: 95.8% (100 epochs); BPNN: 91-100%	Internal test set	High
**Sohi et al. (2022)**	Sheep	Australia	SVM (behavior), Deep Learning (prediction)	Accelerometer	197 ewes (32 + 165)	Prediction confidence: 90% (up to 240 hours before)	Internal test set	Moderate
**Trieu et al. (2022)**	Sheep	New Zealand	Machine Learning	GPS + Accelerometer	20 sheep (10 infected, 6 control)	Qualitative (behavioral changes observed)	Observational	Low
**ZareBidaki et al. (2022)**	Sheep/goat	Iran	Artificial Neural Network (ANN)	Blood and milk samples	200 paired samples	Sensitivity: 81%; Specificity: 62%	Internal test set	Low

Table S4: Prominent Studies in Growth, Weight, and Body Measurement for Sheep and Goats (n=9)

Study (Author, Year)	Species	Country	AI Method / Architecture	Input Data / Modality	n Animals / Samples	Performance Metric & Value	Validation Strategy	Quality Tier
**Ataç et al. (2022)**	Goat	Turkey	Multi-layer feed-forward backpropagation ANN	Morphometric data	12,983 goat kids	R^2 training: 0.98; R^2 validation: 0.97	Internal test set	High
**Erduran et al. (2024)**	Goat	Turkey	Linear Regression, ANN, SVM, Decision Tree, Random Forest, XGBoost, ExtraTree	Morphometric + meteorological data	1,530 goat offspring	ExtraTree R^2: 0.81 (live weight & body length)	Cross-validation	Moderate
**Eroğlu et al. (2025)**	Sheep	Turkey	Random Forest, CART, Gradient Boosting, XGBoost, CatBoost	Biometric measurements (8 traits)	49 Romanov lambs	Gradient Boosting & XGBoost – highest R^2	80/20 split + GridSearchCV (10-fold)	Moderate
**Esener & Kal (2025)**	Goat	Turkey	Random Forest, Extra Trees, XGBoost, CatBoost, LightGBM	Morphometric + weather/grassland data	Not specified	R^2: 0.78–0.80; MAPE: 0.14	Cross-validation	Moderate
**Haldar et al. (2023)**	Goat	India	RPART (Recursive Partitioning and Regression Trees), Stepwise regression	Morphometric data (heart girth, punch girth)	1,427 goats	RPART outperformed stepwise regression (minimal error)	Internal test set	Moderate
**He et al. (2023)**	Sheep	China	LiteHRNet (lightweight CNN) + Class Activation Mapping (CAM)	RGB-D images	726 RGB-D images (19.5–94 kg)	MAPE: 14.605% (1.06M parameters)	Internal test set	High
**Samperio Fernández et al. (2021)**	Sheep	Spain	3D image processing (upper area + average depth)	3D images	272 lambs	Weight estimation error <6%	Internal test set	Moderate
**Vazquez-Martinez et al. (2023)**	Sheep	Mexico	MARS, CART, SVR	Biometric measurements	280 hair sheep	SVR and MARS gave highest R^2; SVR recommended	Internal test set	Moderate
**Wei et al. (2025)**	Sheep	China	PointNet++ (point cloud segmentation) + 3D reconstruction	Multi-view KinectV2 (3 cameras)	35 sheep (augmented to 175)	Avg. relative error: WH 1.67%, CW 3.63%, RH 1.14%, RW 2.71%, BL 3.57%, CG 3.71%	Manual + image-based validation	High

Table S5: Prominent Studies in Genomics & Molecular Biology for Sheep and Goats (n=8)

Study (Author, Year)	Species	Country	AI Method / Architecture	Input Data / Modality	n Animals / Samples	Performance Metric & Value	Validation Strategy	Quality Tier
**Alemu et al. (2025)**	Sheep	New Zealand	Neural Network GBLUP (NN-GBLUP) + PCA-reduced rumen microbial composition	Genomic + microbiome data	1,051 (methane), 984 (RFI), 863 overlapping	Prediction accuracy: methane 0.09→0.30; RFI 0.25→0.34	Cross-validation	High
**Chen et al. (2022)**	Sheep	China	Random Forest, XGBoost (two-step ML)	Non-coding RNA (circRNA, miRNA)	Not specified	Identified 44 circRNAs, 39 miRNAs as biomarkers	Bioinformatics validation	Moderate
**Chen et al. (2023)**	Sheep	China	Random Forest	Multi-omics (microbiome, metabolome, transcriptome)	Not specified	Identified potential biomarkers (GlcADG, ethylmalonic acid, FBLIM1)	Internal validation	Moderate
**Guo et al. (2024)**	Sheep (plus cattle/pigs)	China	AdaBoost, SVM, LR, DT, KNN, DNN, RNN + SHAP	Transcriptomic data (muscle tissues)	275 total (68 sheep)	Precision, Recall, F1-score, SHAP values	Cross-validation	High
**Sananmuang et al. (2025)**	Goat	Thailand/France	Hybrid 1DCNN-GRU	Single-cell RNA-seq (scRNA-seq)	Public scRNA-seq datasets	Accuracy: 98.89%; Precision: 100%; Recall: 97.83%; F1: 98.84%	Internal validation	High
**Tang et al. (2025)**	Goat	China	WGCNA + Machine Learning	Transcriptomic data (RNA-seq)	Tibetan and Yunling goats	Identified key genes (MAP3K5, TGFBR2, RSPO1, ITGB5) and HIF-1 pathway	Bioinformatics validation	Moderate
**Wang et al. (2023a)**	Sheep	China	SVM-RFE, Random Forest, LASSO (feature selection) + ANN (diagnosis)	Gene expression (4 hub genes: ADAM9, CRYL1, NDP52, SERPINA7)	23 samples (two GEO datasets)	AUC: 0.970; ANN accuracy: 95.6%	Internal validation	High
**Zhang et al. (2025b)**	Sheep	China	Neural Network (MLP) + Boruta feature selection + 10×10 CV	Multi-omics (genome + transcriptome)	49 sheep (24 Hetian, 25 Merino)	Neural network accuracy on independent test set; IRF2BP2, EGFR identified	Independent test set + 10×10 CV	High

Table S6: Prominent Studies in Production, Technical Methods & Environmental Applications for Sheep and Goats (n=15)

Study (Author, Year)	Species	Country	AI Method / Architecture	Input Data / Modality	n Animals / Samples	Performance Metric & Value	Validation Strategy	Quality Tier
**Ferreira et al. (2025)**	Sheep	Portugal	Binary + multiclass classifiers (lightweight)	Wearable collar sensor	61 ewes	MCC up to 0.93 (lightweight two-stage system)	Internal test set	Moderate
**Guevara et al. (2023)**	Sheep	Mexico	SMOreg, MLP, Random Forest, Linear Regression	Lactation records	1,186 records (156 lactations)	SMOreg r = 0.96 (total milk yield)	Internal test set	Moderate
**Karamoutsios et al. (2025)**	Sheep/Goat	Greece	Neural Networks	MALDI-TOF MS spectra (milk)	648 (bovine), 1,554 (caprine), 2,392 (ovine) spectra	High level of accuracy in species identification	Internal test set	Moderate
**Louta et al. (2023)**	Sheep/Goat	Greece	Web-based application (software technologies)	Farm management data	Case study (dairy sheep farm)	Feeding cost reduction: 5.5%	Field deployment	Low
**Ma et al. (2025)**	Not specified	Netherlands	ESM (protein), MolFormer (chemical), CLIP (vision)	Peptide sequences, chemical structures, images	Not specified	Peptide transport: 0.89; Taste classification: 0.99	Internal test set	High
**Paz-Kagan et al. (2023)**	Goat	Israel	Random Forest	GPS tracking + Landsat-8 satellite	7 goat herds (6 years)	Stocking rate + distance to corral as primary predictors	Field validation	Moderate
**Pu et al. (2022)**	Goat	China	Improved TPH-YOLOv5 + self-supervised learning	Camera (high-density scenes)	New Chengdu Ma goat dataset	High accuracy in localizing goats in high-density scenes	Internal test set	Moderate
**Sant'Ana et al. (2022)**	Sheep	Brazil	VGG16, ResNet152V2, InceptionV3, DenseNet201	Images (superpixel classification)	32 sheep, 512 images	Best F-score: 0.928 (DenseNet201); IoU: 0.8332	Internal test set	Moderate
**Sarwar et al. (2021)**	Sheep	Australia/Pakistan	7-layer CNN, 1-layer CNN, FCN, AlexNet, GoogLeNet, VGG16, VGG19, ResNet50	UAV aerial images	Not specified	Recall improved from 90% to 98% (merging FCN + proposed network)	Field test (aerial)	High
**Vayssade & Bonneau (2024)**	Goat	France	Puzzle (CNN for appearance + association)	Camera (outdoor tracking)	Not specified	Good tracking >90%	Open-source validation	Moderate
**Wang et al. (2024)**	Sheep	China	UGTransformer (Merge Block + Global Connectivity Module)	Satellite imagery (high resolution)	Not specified	IoU improvement: +1.8% vs UNet, Deeplab v3+, etc.	Internal test set	High
**Xie et al. (2021)**	Sheep	China	MDAU-Net (U-Net + multi-scale dual attention)	Carcass images (hind leg)	Self-built dataset	Precision: 93.76%; MIoU: 86.94%; Offset: 4.02 mm	Internal test set	Moderate
**Yang et al. (2024)**	Goat	China	Molecular docking + deep learning (screening)	Peptide sequence (FGMp11)	Not mentioned	Significant inhibition of S. aureus in pasteurized milk	Laboratory validation	Moderate
**Zhao et al. (2023a)**	Sheep	China	SheepInst (Mask R-CNN/RefineMask + ConvNeXt-E + Spatial Attention)	Images (sheep instance segmentation)	1,396 images (Hu sheep)	Box AP: 89.1%; Mask AP: 91.3%; Boundary AP: 79.5%	Internal test set	High
**Zhao et al. (2023b)**	Sheep	China	Swin-Transformer (Swin-T, Swin-B, Swin-S) + transfer learning	Mutton images	Not specified	mAP: 0.943 (Swin-S); Processing time: 0.25s/image	Internal test set	High

Table S7. Publicly Available Datasets Supporting AI Research in Small Ruminants (n=24)

Dataset Name & Lead Author	Species	Domain	Size / Records	Access	Verified URL / DOI	Citation / Journal
			**BEHAVIOR & ACTIVITY RECOGNITION — 6 datasets**			
**Sheep Lameness-Gait Dataset (Kaler et al., 2020)**	Sheep	Gait / Lameness Detection	23 animals (13 lame, 10 non-lame); ear IMU accelerometer & gyroscope @ 16 Hz	Open (DOI)	doi.org/10.5061/dryad.mk4fc3r	Kaler et al. (2020). R. Soc. Open Sci. 7(1):190824. DOI: 10.1098/rsos.190824
**VOCAPRA – Goat Vocalization Dataset (Ntalampiras & Gamacchio, 2025)**	Goat (Alpine, Saanen)	Acoustic / Behavior Classification	4,147 WAV files (2 s each); 8 welfare-state classes; 4 farms, Italy	Open	zenodo.org/records/7530401	Ntalampiras & Gamacchio (2025). PLoS ONE 20(4):e0318543. DOI: 10.1371/journal.pone.0318543
**Sheep Grazing Behavior – IMU + GPS Dataset (Jin et al., 2024)**	Sheep	Behavior Classification (IMU + GPS)	4 sensor positions; 20 Hz; 6 behaviors; neck & leg mounted IMU; CNN-LSTM	Contact Author	doi.org/10.1016/j.compag.2024.108894	Jin et al. (2024). Comput. Electron. Agric. 220:108894. DOI: 10.1016/j.compag.2024.108894
**Goat Accelerometer Behavior Dataset (Hollevoet et al., 2024)**	Goat	Activity Recognition (Accelerometer)	Labeled accelerometer data; free-range goat activity; multiple ML models tested	Contact Author	doi.org/10.3390/ani14131977	Hollevoet et al. (2024). Animals 14(13):1977. DOI: 10.3390/ani14131977
**Sheep Grazing & Rumination Dataset (Machine Learning) (Hu et al., 2020)**	Sheep	Behavior Classification (Multiple sensors)	Sensor data from grazing sheep; multiple time-window sizes; labeled behaviors	Contact Author	doi.org/10.1016/j.compag.2020.105857	Hu et al. (2020). Comput. Electron. Agric. 179:105857. DOI: 10.1016/j.compag.2020.105857
**Goat Behavior Video Dataset (Jiang et al., 2020)**	Goat	Group Behavior Recognition (Video)	Video of group-housed goats; deep learning behavioral classification	Contact Author	doi.org/10.1016/j.compag.2020.105706	Jiang et al. (2020). Comput. Electron. Agric. 177:105706. DOI: 10.1016/j.compag.2020.105706
			**INDIVIDUAL ANIMAL IDENTIFICATION — 4 datasets**			
**CherryChèvre – Goat Detection Dataset (Vayssade et al., 2023)**	Goat (3 breeds)	Detection / Individual Tracking	6,160 annotated images; varying light & conditions; bounding box annotations; open benchmark	Open	doi.org/10.1038/s41597-023-02555-8 Code: gitlab.com/inrae-asset/puzzle-livestock-tracking	Vayssade et al. (2023). Sci. Data 10:689. DOI: 10.1038/s41597-023-02555-8
**Puzzle – Livestock Tracking Dataset (Vayssade & Bonneau, 2024)**	Goat	Multi-object Tracking (Video)	Video sequences; CNN-based individual appearance tracking; linked to CherryChèvre dataset	Open (GitHub)	gitlab.com/inrae-asset/puzzle-livestock-tracking	Vayssade & Bonneau (2024). Sci. Rep. 14:18348. DOI: 10.1038/s41598-024-69058-3
**Goat Face & Sheep Face Detection Dataset (Wang et al., 2023b)**	Goat & Sheep	Face Detection / Individual ID	Images in natural environment; small-target goat face detection;	Contact Author	doi.org/10.3390/ani13142365	Wang et al. (2023). Animals 13(14):2365. DOI: 10.3390/ani13142365

Dataset Name & Lead Author	Species	Domain	Size / Records	Access	Verified URL / DOI	Citation / Journal
			contextualized network			
**Goat Retinal Identification Dataset (Mustafi et al., 2021)**	Goat	Biometric ID (Retinal images)	Retinal images of individual goats; unique biometric identification; RetIS system; 99% accuracy	Contact Author	doi.org/10.1016/j.compag.2021.106127	Mustafi et al. (2021). Comput. Electron. Agric. 185:106127. DOI: 10.1016/j.compag.2021.106127
**HEALTH, WELFARE & DISEASE DETECTION — 4 datasets**						
**Sheep GIN Parasite Sensor Dataset (Fogarty et al., 2023)**	Sheep	Parasite Detection (H. contortus / Barber's pole worm)	Sensor-based (GPS + accelerometer); Haemonchus contortus infection; behavioral changes detected	Contact Author	doi.org/10.1016/j.atech.2023.100112	Fogarty et al. (2023). Smart Agric. Technol. 3:100112. DOI: 10.1016/j.atech.2023.100112
**Sheep Haemonchosis Mobile App Dataset (de Souza et al., 2023)**	Sheep	GIN / Haemonchosis TST Detection	Mobile app data; targeted selective treatment; FAMAACHA scoring; field-validated	Contact Author	doi.org/10.1016/j.vetpar.2023.109902	de Souza et al. (2023). Vet. Parasitol. 316:109902. DOI: 10.1016/j.vetpar.2023.109902
**Subclinical Mastitis – Sheep Farm ML Dataset (Kiouvrekis et al., 2024)**	Sheep (dairy)	Mastitis Prediction (Subclinical)	Dairy sheep farm data; ML-predicted subclinical mastitis prevalence; somatic cell count + farm	Contact Author	doi.org/10.3390/ani14162295	Kiouvrekis et al. (2024). Animals 14(16):2295. DOI: 10.3390/ani14162295
**Bulk-Tank Milk Quality XAI Dataset (Lianou et al., 2024)**	Sheep & Goat	Milk Quality Prediction (XAI)	Bulk-tank milk data from sheep and goat farms; explainable ML (SHAP); milk quality classification	Contact Author	doi.org/10.3390/foods13244015	Lianou et al. (2024). Foods 13(24):4015. DOI: 10.3390/foods13244015
**GROWTH, WEIGHT & BODY MEASUREMENT — 4 datasets**						
**SheepInst – Sheep Instance Segmentation Dataset (Zhao et al., 2023a)**	Sheep	Instance Segmentation / Body Contour	Images of multiple sheep (overlap scenarios); bounding boxes + masks; high AP performance	Contact Author	doi.org/10.3390/ani13081338	Zhao et al. (2023). Animals 13(8):1338. DOI: 10.3390/ani13081338
**Sheep Weight Estimation RGB-D Dataset (He et al., 2023)**	Sheep	Live Weight Estimation (RGB-D)	RGB-D images (depth camera); enhanced LiteHRNet; weight regression from body landmarks	Contact Author	doi.org/10.1016/j.compag.2023.107667	He et al. (2023). Comput. Electron. Agric. 206:107667. DOI: 10.1016/j.compag.2023.107667
**Sheep 3D Body Measurement (Point Cloud) Dataset (Wei et al., 2025)**	Sheep	3D Body Size Measurement (Point Cloud)	3D reconstruction from multi-view; PointNet++	Contact Author	doi.org/10.1016/j.compag.2025.110978	Wei et al. (2025). Comput. Electron. Agric. 239:110978. DOI: 10.1016/j.compag.2025.110978

Dataset Name & Lead Author	Species	Domain	Size / Records	Access	Verified URL / DOI	Citation / Journal
			segmentation; automated body dimension extraction			
**Lamb Live Weight 3D Estimation Dataset (Samperio et al., 2021)**	Sheep (Lambs)	Live Weight Estimation (3D)	3D image-based weight estimation of lambs; non-contact measurement; commercial validation	Contact Author	doi.org/10.1016/j.animal.2021.100212	Samperio et al. (2021). Animal 15(5):100212. DOI: 10.1016/j.animal.2021.100212
**GENOMICS & MOLECULAR BIOLOGY — 3 datasets**						
**International Sheep Genomics Consortium (ISGC) Dataset**	Sheep (69+ breeds)	Genomics / SNP Array	935 sheep; 69 breeds; 21 countries; Illumina 15K, 50K & HD 600K SNP chips; ~50M variants	Open (Regist.)	sheephapmap.org Registration may be required for WGS data	Kijas et al. (2012). ISGC. sheephapmap.org — Genome assemblies + VCF
**AdaptMap – Global Goat SNP50 BeadChip Dataset**	Goat (144+ breeds)	Genomics / SNP Array	4,653 animals; 169 popns; 35 countries; 6 continents; CaprineSNP50 (53,347 SNPs); open via BioProject	Open	goatadaptmap.org Raw data: NCBI BioProject PRJNA290100	Colli et al. (2018). Genet. Sel. Evol. 50:57. DOI: 10.1186/s12711-018-0422-x
**Sheep Genomic Prediction – Rumen Microbiome Dataset (Alemu et al., 2025)**	Sheep	Genomics + Microbiome (Methane / Feed)	Host genomic variation + rumen microbial PCA; methane & feed efficiency; NN-GBLUP prediction	Contact Author	doi.org/10.1186/s12711-025-01041-y	Alemu et al. (2025). Genet. Sel. Evol. 57:41. DOI: 10.1186/s12711-025-01041-y
**PRODUCTION, DETECTION & ENVIRONMENTAL APPLICATIONS — 3 datasets**						
**Sheep UAV Detection Dataset (Sarwar et al., 2021)**	Sheep	UAV Detection / Counting	Aerial images at 80 m and 120 m altitude; multiple CNN networks; U-Net-MS F1 ~98%	Contact Author	doi.org/10.1016/j.compag.2021.106219	Sarwar et al. (2021). Comput. Electron. Agric. 187:106219. DOI: 10.1016/j.compag.2021.106219
**UGTransformer – Sheep Satellite Imagery Dataset (Wang et al., 2024)**	Sheep	Remote Sensing / Extraction (Satellite)	Satellite images; transformer architecture; sheep extraction for animal husbandry management (IEEE TGRS)	Contact Author	doi.org/10.1109/TGRS.2024.3405400	Wang et al. (2024). IEEE Trans. Geosci. Remote Sens. 62:1–14. DOI: 10.1109/TGRS.2024.3405400
**Goat Herd Detection & Tracking Dataset (Hou et al., 2025)**	Goat	Herd Detection / Tracking (Video)	Dynamic herd detection; BiAF-YOLOv7 algorithm; natural grassland pastures;	Contact Author	doi.org/10.1038/s41598-025-89231-6	Hou et al. (2025). Sci. Rep. 15:4754. DOI: 10.1038/s41598-025-89231-6

Dataset Name & Lead Author	Species	Domain	Size / Records	Access	Verified URL / DOI	Citation / Journal
			Precision=94.5%, mAP=96%			

Table S8 — Quality Assessment Instrument and Study-Level Ratings

Quality assessment completed: May 2026. Final arithmetic verification: May 2026.
Verification code (Python script for score summation and tier assignment) is available from the corresponding author upon reasonable request.

Part A: Quality Scoring Instrument

Eight domains were assessed on a 0–2 scale (maximum 16 points). Composite score thresholds were determined a priori prior to data extraction.

Domain	Description	Score 0	Score 1	Score 2
**D1**	**AI Methodology Clarity**	Not described or unidentifiable	Partially described; key parameters missing	Fully described; reproducible from manuscript
**D2**	**Sample Size Adequacy**	< 10 animals/samples	10–30 animals or limitation acknowledged	> 30 animals or statistical justification provided
**D3**	**Validation Strategy Rigor**	No separation or proof-of-concept only	K-fold CV or described internal test set	Independent hold-out, LOOCV on unseen animals, cross-farm, or field deployment
**D4**	**Performance Metric Transparency**	Single metric only; no variability reported	Multiple metrics but no CI or SD	Multiple metrics with variability measures or statistical comparisons
**D5**	**Input Data Quality**	No description of acquisition conditions	Partial description; resolution/calibration unclear	Full description of acquisition conditions, resolution, and preprocessing
**D6**	**Experimental Design and Confound Control**	No controls; obvious confounders unaddressed	Partial controls; some confounders acknowledged	Appropriate controls; confounders identified and addressed
**D7**	**Generalizability**	Tested only on training-distribution data	Tested on held-out subset from same population	Tested on unseen animals, breeds, farms, or real-world deployment
**D8**	**Conclusion–Result Alignment**	Conclusions substantially exceed reported evidence	Minor overclaiming; mostly supported	Conclusions fully supported by reported results

Composite Score Thresholds (determined a priori)

Quality Category	Score Range	Interpretation
**High Quality**	**≥ 13 points**	Rigorous methodology across all critical dimensions
**Moderate Quality**	**8–12 points**	Meets most criteria with minor limitations
**Low Quality**	**≤ 7 points**	Significant methodological deficiencies

Part B: Study-Level Ratings (n = 92)

All 92 composite scores were independently verified by summing D1–D8. Color coding: green = High (≥13), orange = Moderate (8–12), red = Low (≤7).

Behavior & Activity Recognition (n = 24)											
#	Study	D1	D2	D3	D4	D5	D6	D7	D8	Total	Tier
1	Bati & Ser (2023)	2	2	1	1	2	1	1	1	11	**Moderate**
2	Bonneau et al. (2024)	2	2	2	2	2	1	2	2	15	**High**
3	do Nascimento Amorim et al. (2025)	2	1	1	2	2	1	1	2	12	**Moderate**
4	Gu et al. (2023)	2	1	1	1	1	1	2	1	10	**Moderate**
5	Guo et al. (2025)	2	2	1	1	2	1	1	2	12	**Moderate**
6	Hollevoet et al. (2024)	2	**0**	2	2	2	1	2	2	13	**High**
7	Hu et al. (2020)	2	1	1	1	2	**0**	**0**	1	8	**Moderate**
8	Jiang et al. (2020)	2	1	1	1	2	1	1	2	11	**Moderate**
9	Jin et al. (2024)	2	1	2	2	2	1	2	2	14	**High**
10	Mao et al. (2023)	2	1	1	2	2	1	1	2	12	**Moderate**
11	Méndez et al. (2025)	2	1	1	2	2	1	1	2	12	**Moderate**
12	Nóbrega et al. (2020)	2	1	2	1	2	1	2	2	13	**High**
13	Price et al. (2022)	2	2	2	1	2	1	1	2	13	**High**
14	Ser et al. (2025)	2	2	2	1	2	1	1	2	13	**High**
15	Sun et al. (2025a)	2	1	1	1	2	1	1	2	11	**Moderate**
16	Sun et al. (2025b)	2	1	1	1	2	1	1	2	11	**Moderate**
17	Suparwito et al. (2021)	1	**0**	1	**0**	1	**0**	**0**	1	4	**Low**
18	Turner et al. (2023)	2	1	2	2	2	1	2	2	14	**High**
19	Wang et al. (2021)	2	**0**	1	1	2	1	1	2	10	**Moderate**
20	Xie et al. (2025)	2	1	**0**	**0**	2	**0**	**0**	1	6	**Low**
21	Xu et al. (2023)	2	1	2	1	2	1	1	2	12	**Moderate**
22	Yan et al. (2025)	2	1	1	1	2	1	1	2	11	**Moderate**
23	Zhang et al. (2021)	2	1	1	1	2	1	1	2	11	**Moderate**
24	Zhang et al. (2024a)	1	1	**0**	**0**	1	**0**	**0**	1	4	**Low**
**Behavior Summary: High = 7 (29.2%) Moderate = 14 (58.3%) Low = 3 (12.5%)**										**n = 24**	
**Individual Animal Identification (n = 18)**											
#	Study	D1	D2	D3	D4	D5	D6	D7	D8	Total	Tier
25	Billah et al. (2022)	2	1	1	1	2	1	1	2	11	**Moderate**
26	Himel et al. (2024)	2	2	2	1	2	1	1	2	13	**High**
27	Hou et al. (2025)	2	1	1	2	2	1	1	2	12	**Moderate**
28	Huang et al. (2023)	2	1	1	2	2	1	2	1	12	**Moderate**
29	Li et al. (2023)	2	2	1	2	2	1	1	2	13	**High**
30	Li et al. (2024a)	2	2	1	2	2	1	2	2	14	**High**

#	Study	D1	D2	D3	D4	D5	D6	D7	D8	Total	Tier
31	Li et al. (2024b)	2	1	1	2	2	1	1	2	12	**Moderate**
32	Mustafi et al. (2021)	2	**0**	1	2	2	1	2	1	11	**Moderate**
33	Ntalampiras & Pesando (2025)	2	2	2	2	2	1	2	2	15	**High**
34	Pang et al. (2023)	2	2	1	1	2	1	1	2	12	**Moderate**
35	Sun et al. (2023b)	2	1	1	2	2	1	2	1	12	**Moderate**
36	Wang et al. (2023b)	2	2	1	1	2	1	1	2	12	**Moderate**
37	Yadav et al. (2024)	2	2	2	2	1	1	2	1	13	**High**
38	Yuan et al. (2025)	2	2	2	2	2	1	2	2	15	**High**
39	Zhang et al. (2022)	2	2	1	1	2	1	1	2	12	**Moderate**
40	Zhang et al. (2023)	2	2	1	2	2	1	1	2	13	**High**
41	Zhang et al. (2024b)	2	2	1	2	2	1	2	2	14	**High**
42	Zhang et al. (2025a)	2	1	1	1	2	1	1	2	11	**Moderate**
**Individual Summary: High = 8 (44.4%) Moderate = 10 (55.6%) Low = 0 (0.0%)**										**n = 18**	
**Health, Welfare & Disease (n = 18)**											
**#**	**Study**	**D1**	**D2**	**D3**	**D4**	**D5**	**D6**	**D7**	**D8**	**Total**	**Tier**
43	Assefa et al. (2021)	2	2	2	2	2	2	2	2	16	**High**
44	Betancor-Sánchez et al. (2025)	2	2	1	1	2	1	1	2	12	**Moderate**
45	Bucki et al. (2023)	2	2	2	1	2	1	1	2	13	**High**
46	de Souza et al. (2023)	2	2	1	1	2	1	1	2	12	**Moderate**
47	Emsen et al. (2025)	2	2	1	**0**	1	1	**0**	1	8	**Moderate**
48	Farr et al. (2025)	2	1	1	2	2	2	1	2	13	**High**
49	Fogarty et al. (2021)	2	2	1	1	2	1	1	2	12	**Moderate**
50	Fogarty et al. (2023)	2	2	1	**0**	2	1	**0**	1	9	**Moderate**
51	Gurule et al. (2021)	2	1	1	**0**	2	1	**0**	1	8	**Moderate**
52	Kaler et al. (2020)	2	1	1	1	2	1	1	2	11	**Moderate**
53	Kiouvrekis et al. (2024)	2	2	1	2	2	1	1	2	13	**High**
54	Lianou et al. (2024)	2	2	1	1	2	1	1	2	12	**Moderate**
55	Lysitsas et al. (2024)	2	2	1	1	2	1	1	2	12	**Moderate**
56	Martone et al. (2025)	2	2	1	1	1	1	**0**	1	9	**Moderate**
57	Siddique et al. (2024)	2	1	1	2	2	1	2	1	12	**Moderate**
58	Sohi et al. (2022)	2	2	1	1	2	1	1	2	12	**Moderate**
59	Trieu et al. (2022)	1	1	**0**	**0**	1	**0**	**0**	1	4	**Low**
60	ZareBidaki et al. (2022)	2	2	1	**0**	1	**0**	1	1	8	**Moderate**
**Health, Summary: High = 4 (22.2%) Moderate = 13 (72.2%) Low = 1 (5.6%)**										**n = 18**	
**Growth & Body Measurement (n = 9)**											
**#**	**Study**	**D1**	**D2**	**D3**	**D4**	**D5**	**D6**	**D7**	**D8**	**Total**	**Tier**
61	Ataç et al. (2022)	2	2	2	2	2	1	1	2	14	**High**
62	Erduran et al. (2024)	2	2	2	1	2	1	1	2	13	**High**
63	Eroğlu et al. (2025)	2	1	2	1	2	1	1	2	12	**Moderate**

#	Study	D1	D2	D3	D4	D5	D6	D7	D8	Total	Tier
64	Esener & Kal (2025)	2	1	2	1	2	1	1	2	12	**Moderate**
65	Haldar et al. (2023)	2	2	1	1	2	1	1	2	12	**Moderate**
66	He et al. (2023)	2	1	1	2	2	1	2	1	12	**Moderate**
67	Samperio et al. (2021)	2	2	1	1	2	1	1	2	12	**Moderate**
68	Vazquez-Martinez et al. (2023)	2	2	1	1	2	1	1	2	12	**Moderate**
69	Wei et al. (2025)	2	1	2	2	2	1	1	2	13	**High**
**Growth Summary: High = 3 (33.3%) Moderate = 6 (66.7%) Low = 0 (0.0%)**										**n = 9**	
**Genomics & Molecular Biology (n = 8)**											
**#**	**Study**	**D1**	**D2**	**D3**	**D4**	**D5**	**D6**	**D7**	**D8**	**Total**	**Tier**
70	Alemu et al. (2025)	2	2	2	2	2	2	1	2	15	**High**
71	Chen et al. (2022)	2	1	1	1	2	1	1	2	11	**Moderate**
72	Chen et al. (2023)	2	1	1	1	2	1	1	2	11	**Moderate**
73	Guo et al. (2024)	2	2	2	2	2	1	1	2	14	**High**
74	Sananmuang et al. (2025)	2	1	1	2	2	1	1	2	12	**Moderate**
75	Tang et al. (2025)	2	1	1	1	2	1	1	2	11	**Moderate**
76	Wang et al. (2023a)	2	1	1	2	2	1	2	1	12	**Moderate**
77	Zhang et al. (2025b)	2	1	2	2	2	1	2	2	14	**High**
**Genomics Summary: High = 3 (37.5%) Moderate = 5 (62.5%) Low = 0 (0.0%)**										**n = 8**	
**Production & Technical (n = 15)**											
**#**	**Study**	**D1**	**D2**	**D3**	**D4**	**D5**	**D6**	**D7**	**D8**	**Total**	**Tier**
78	Ferreira et al. (2025)	2	1	2	1	2	1	1	2	12	**Moderate**
79	Guevara et al. (2023)	2	2	1	1	2	1	1	2	12	**Moderate**
80	Karamoutsios et al. (2025)	2	2	1	1	2	1	1	2	12	**Moderate**
81	Louta et al. (2023)	1	1	1	**0**	1	**0**	**0**	1	5	**Low**
82	Ma et al. (2025)	2	1	1	2	2	1	2	1	12	**Moderate**
83	Paz-Kagan et al. (2023)	2	2	2	2	2	1	2	1	14	**High**
84	Pu et al. (2022)	2	1	1	1	2	1	1	2	11	**Moderate**
85	Sant'Ana et al. (2022)	2	1	2	1	2	1	1	2	12	**Moderate**
86	Sarwar et al. (2021)	2	1	2	1	2	1	2	2	13	**High**
87	Vayssade & Bonneau (2024)	2	1	2	1	2	1	1	2	12	**Moderate**
88	Wang et al. (2024)	2	1	1	2	2	1	2	1	12	**Moderate**
89	Xie et al. (2021)	2	1	1	1	2	1	1	2	11	**Moderate**
90	Yang et al. (2024)	2	1	1	1	2	1	1	2	11	**Moderate**
91	Zhao et al. (2023a)	2	1	1	2	2	1	2	1	12	**Moderate**
92	Zhao et al. (2023b)	2	1	1	2	2	1	2	1	12	**Moderate**
**Production Summary: High = 2 (13.3%) Moderate = 12 (80.0%) Low = 1 (6.7%)**										**n = 15**	

Note: *D1=AI Methodology Clarity, D2=Sample Size Adequacy, D3=Validation Strategy Rigor, D4=Performance Metric Transparency, D5=Input Data Quality, D6=Experimental Design, D7=Generalizability, D8=Conclusion–Result Alignment. All scores range 0–2. Total = sum of D1–D8 (max = 16).*

Part C: Summary Statistics

Quality Category	Score Range	Count	Percentage
**High Quality**	≥ 13	**27**	**29.3%**
**Moderate Quality**	8–12	**60**	**65.2%**
**Low Quality**	≤ 7	**5**	**5.4%**
**Total**	**0–16**	**92**	**100%**

Note: *All counts verified by independent arithmetic summation of D1–D8 scores for each study. No manual overrides applied; thresholds are applied mechanically.*

Score Distribution

Score	n	Quality Tier	Cumulative %
**4**	**3**	Low	3.3%
**5**	**1**	Low	4.3%
**6**	**1**	Low	5.4%
**8**	**4**	Moderate	9.8%
**9**	**2**	Moderate	12.0%
**10**	**2**	Moderate	14.1%
**11**	**16**	Moderate	31.5%
**12**	**36**	Moderate	70.7%
**13**	**14**	High	85.9%
**14**	**8**	High	94.6%
**15**	**4**	High	98.9%
**16**	**1**	High	100.0%

Note: *Mode = 12 (n=36, 39.1%); Median = 12; Mean ≈ 11.8; Range = 4–16; No study scored 7.*

Part D: Quality Distribution by Research Domain

Research Domain	n	High Quality	Moderate Quality	Low Quality
**Behavior & Activity Recognition**	**24**	**7** (29.2%)	**14** (58.3%)	**3** (12.5%)
**Individual Animal Identification**	**18**	**8** (44.4%)	**10** (55.6%)	**0** (0.0%)
**Health, Welfare & Disease**	**18**	**4** (22.2%)	**13** (72.2%)	**1** (5.6%)
**Growth & Body Measurement**	**9**	**3** (33.3%)	**6** (66.7%)	**0** (0.0%)
**Genomics & Molecular Biology**	**8**	**3** (37.5%)	**5** (62.5%)	**0** (0.0%)
**Production & Technical**	**15**	**2** (13.3%)	**12** (80.0%)	**1** (6.7%)
**TOTAL**	**92**	**27** (29.3%)	**60** (65.2%)	**5** (5.4%)

Note: *Domain with highest High% = Individual Animal Identification (44.4%). Domain with lowest High% = Production & Technical (13.3%). Three domains have zero Low-quality studies (Identification, Growth, Genomics).*

Mean Domain Scores (D1–D8) by Research Group

Research Domain	D1	D2	D3	D4	D5	D6	D7	D8
Behavior & Activity	**1.92**	1.08	1.25	1.17	1.88	**0.83**	1.08	1.75
Individual Animal Identification	**2.00**	1.56	1.22	1.67	**1.94**	1.00	1.44	1.78
Health, Welfare &	**1.94**	1.72	1.06	**0.94**	1.78	1.00	**0.83**	1.61
Growth & Body	**2.00**	1.56	1.56	1.33	**2.00**	1.00	1.11	1.89
Genomics & Molecular	**2.00**	1.25	1.38	1.62	**2.00**	1.12	1.25	1.88
Production & Technical	**1.93**	1.20	1.33	1.27	**1.93**	**0.93**	1.33	1.60
**Domain Mean**	**1.96**	**1.39**	**1.30**	**1.33**	**1.92**	**0.98**	**1.17**	**1.75**

Note: *Color coding: green ≥ 1.80 (strong), orange 1.20–1.79 (moderate), red < 1.20 (weak). D6 (Experimental Design) is the weakest domain overall (grand mean 0.98). D1 and D5 are universally strong (≥ 1.88).*

Part E: Quality Distribution by Validation Strategy (D3 Score)

D3	Description	n	High Quality	Moderate Quality	Low Quality
**D3=0**	No separation or proof-of-concept only	**3**	**0** (0.0%)	**0** (0.0%)	**3** (100.0%)
**D3=1**	K-fold CV or described internal test set	**62**	**6** (9.7%)	**54** (87.1%)	**2** (3.2%)
**D3=2**	Independent hold-out, LOOCV on unseen animals, cross-farm, or field deployment	**27**	**21** (77.8%)	**6** (22.2%)	**0** (0.0%)
**Total**	—	**92**	**27** (29.3%)	**60** (65.2%)	**5** (5.4%)

Note: *A monotonic relationship exists between D3 score and overall quality tier: D3=0 → 100% Low; D3=1 → 87.1% Moderate; D3=2 → 77.8% High. Validation strategy is the single strongest predictor of composite quality.*

Part F: Explanatory Notes

The following four studies were discussed during quality review as potentially non-intuitive. All final tier assignments are based strictly on the arithmetic composite score against the a priori thresholds. No expert overrides have been applied in the final version.

Study	Score / Tier	Methodological Note	Final Decision
**Price et al. (2022)**	**13 / High**	Activity classification accuracy was 70.9% ± 14.06% SD — high individual variability. Score of 13 reflects methodological rigor (LOOCV, n=196), not outcome quality.	High. Score mechanically meets threshold. Performance limitation does not alter D-score assignment under the instrument.
**Mustafi et al. (2021)**	**11 / Moderate**	Retinal biometric system achieved 99% accuracy (Precision=100%); D2=0 (only 12 goats). Biometric uniqueness is exceptional but sample size is a genuine limitation.	Moderate. Score 11 correctly reflects the D2=0 penalty. D4=2 was applied (full precision/recall tables provided).
**Bucki et al. (2023)**	**13 / High**	n=105 faecal samples, independent lab comparison (D3=2, D6=1). Lead authors affiliated with Micron Agritech (potential COI). Single farm limits D7.	High. Score 13 meets threshold under the instrument. COI is noted but not a scored domain. Tier assignment stands.
**Paz-Kagan et al. (2023)**	**14 / High**	Environmental remote-sensing study: 7 flocks × 6 years, RF regression, R^2=0.93, field validation. D3=2, D6=1 (multi-factor environmental confounders partially controlled).	High. Score 14 clearly meets threshold. The environmental scope does not disqualify the study from the livestock AI framework.

Figure S1. Overall Study Quality Distribution (Donut Chart, n=92).

Proportional donut chart of the overall quality distribution across all 92 included studies. The majority of studies fall in the Moderate quality tier (60 studies, 65.2%), reflecting generally adequate but not fully rigorous methodology. High-quality studies account for 27 (29.3%) and Low-quality for 5 (5.4%). Quality thresholds were determined a priori: High ≥13 points, Moderate 8–12 points, Low ≤7 points out of a maximum of 16. Note: The original Part C of Table S8 stated High=24 (26.1%) and Low=8 (8.7%); the verified counts are High=27 and Low=5.

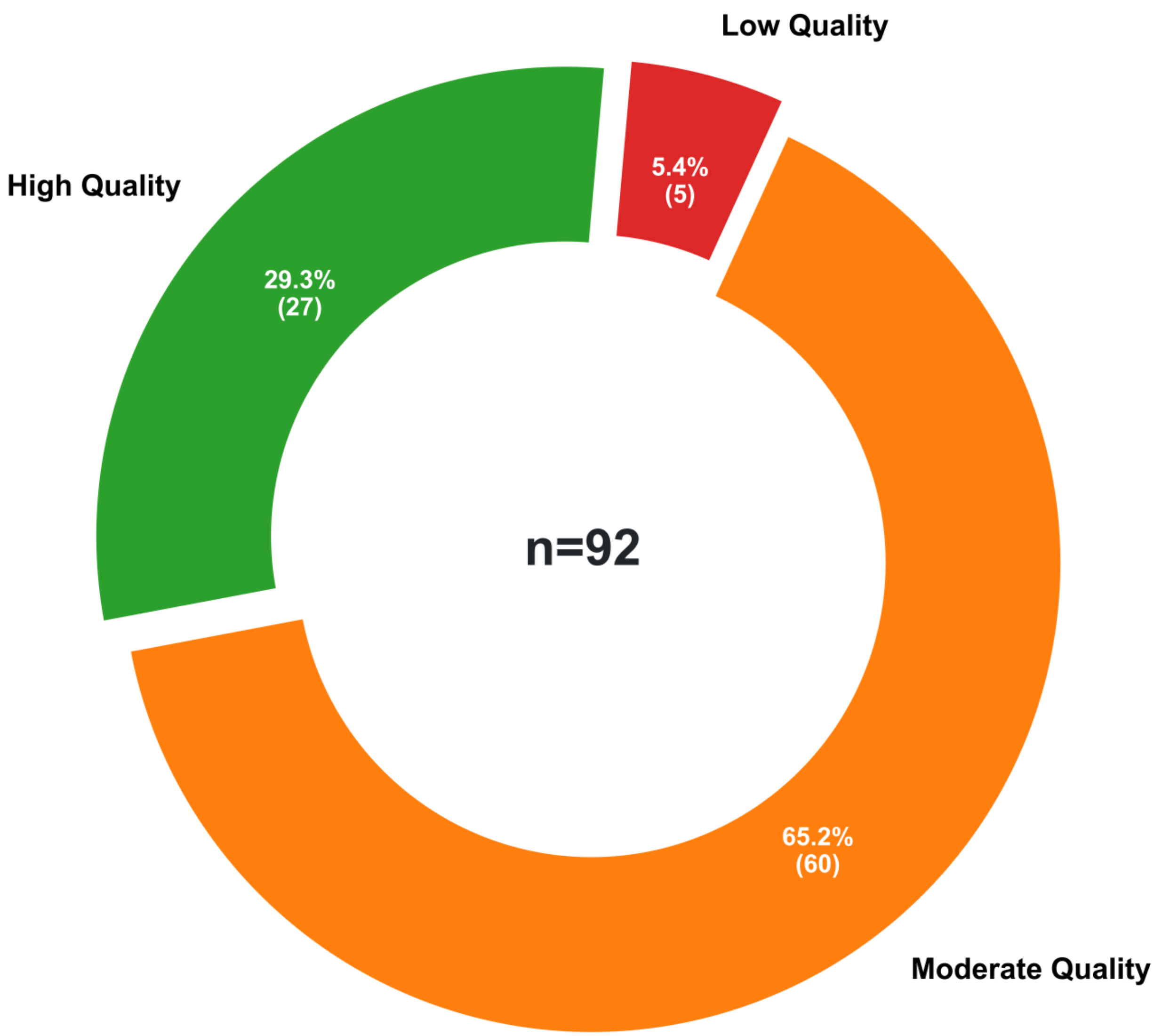

Verified from raw score data. High ≥ 13 pts | Moderate 8–12 pts | Low ≤ 7 pts

Figure S2: Study Quality Distribution by Research Group (Absolute Counts)

Stacked bar chart showing the absolute number of studies rated Low (red), Moderate (orange), and High (green) quality in each of the six research groups. Numbers inside each segment indicate the count and percentage within that group. Behavior & Activity (n=24) contains the most studies and shows the widest spread across tiers. Individual Identification achieves the highest proportion of High-quality studies (8/18, 44.4%). Production & Technical has the lowest High-quality rate (2/15, 13.3%). Grand total: n=92; High=27 (29.3%), Moderate=60 (65.2%), Low=5 (5.4%). Based on verified study-level data.

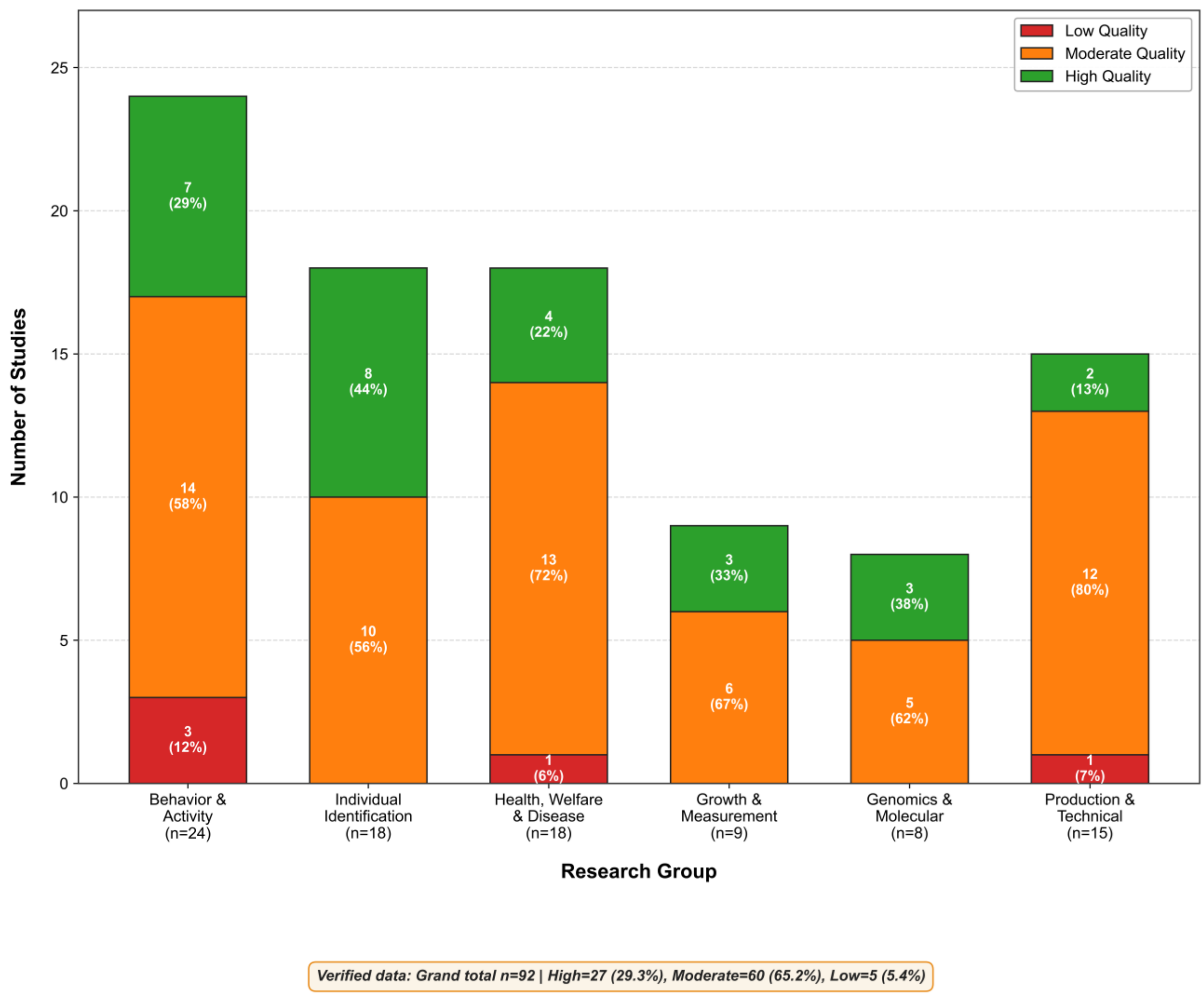

Figure S3: Distribution of Composite Quality Scores (Histogram)

Frequency histogram of composite quality scores (sum of D1–D8 domains, range 0–16). Bar colours denote tier: red = Low (≤7), orange = Moderate (8–12), green = High (≥13). The distribution is left-skewed with a pronounced mode at score 12 (n=36 studies, 39.1%). Scores range from 4 (minimum) to 16 (maximum, only 1 study: Assefa et al. 2021). No studies scored 7, and only 5 studies fall in the Low tier (scores 4–6). Median score = 12; mean ≈ 11.8. Dashed lines mark the tier boundaries.

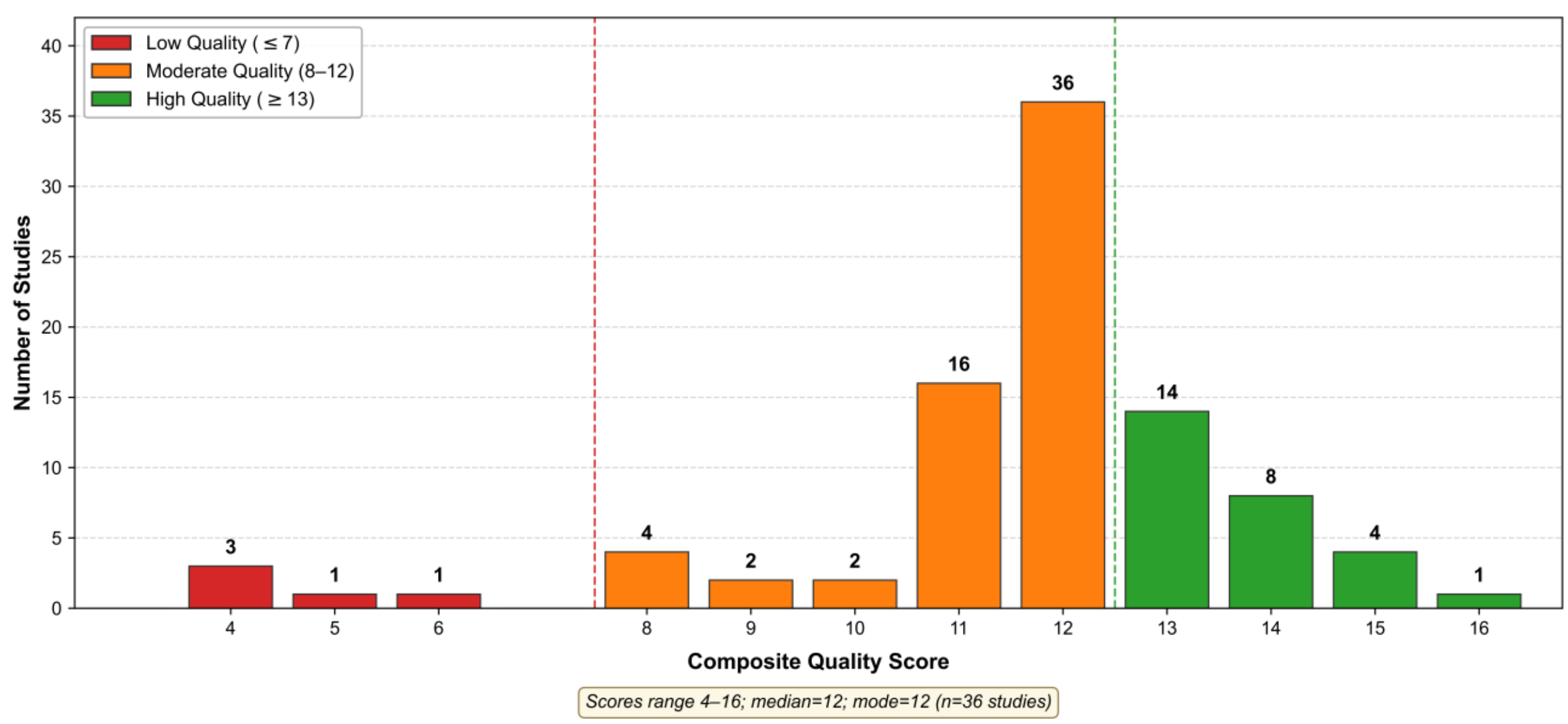

Figure S4. Mean Domain Scores (D1–D8) by Research Group — Heatmap.

Color-coded heatmap of mean scores (0–2) for each of the eight quality domains across all six research groups. Green = high mean (approaching 2.0), red = low mean (approaching 0). Values shown are group means computed from individual study scores. Key findings: D1 (Methodology) and D5 (Data Quality) are universally strong (≥1.9) across all groups. D6 (Experimental Design) is the weakest domain overall (means 0.83–1.12), indicating that confound control is a widespread limitation. D4 (Metric Transparency) is notably low in Health, Welfare & Disease (0.94). D3 (Validation) varies most between groups, with Growth & Measurement performing best (1.56). Based on verified study-level scores; n = 92.

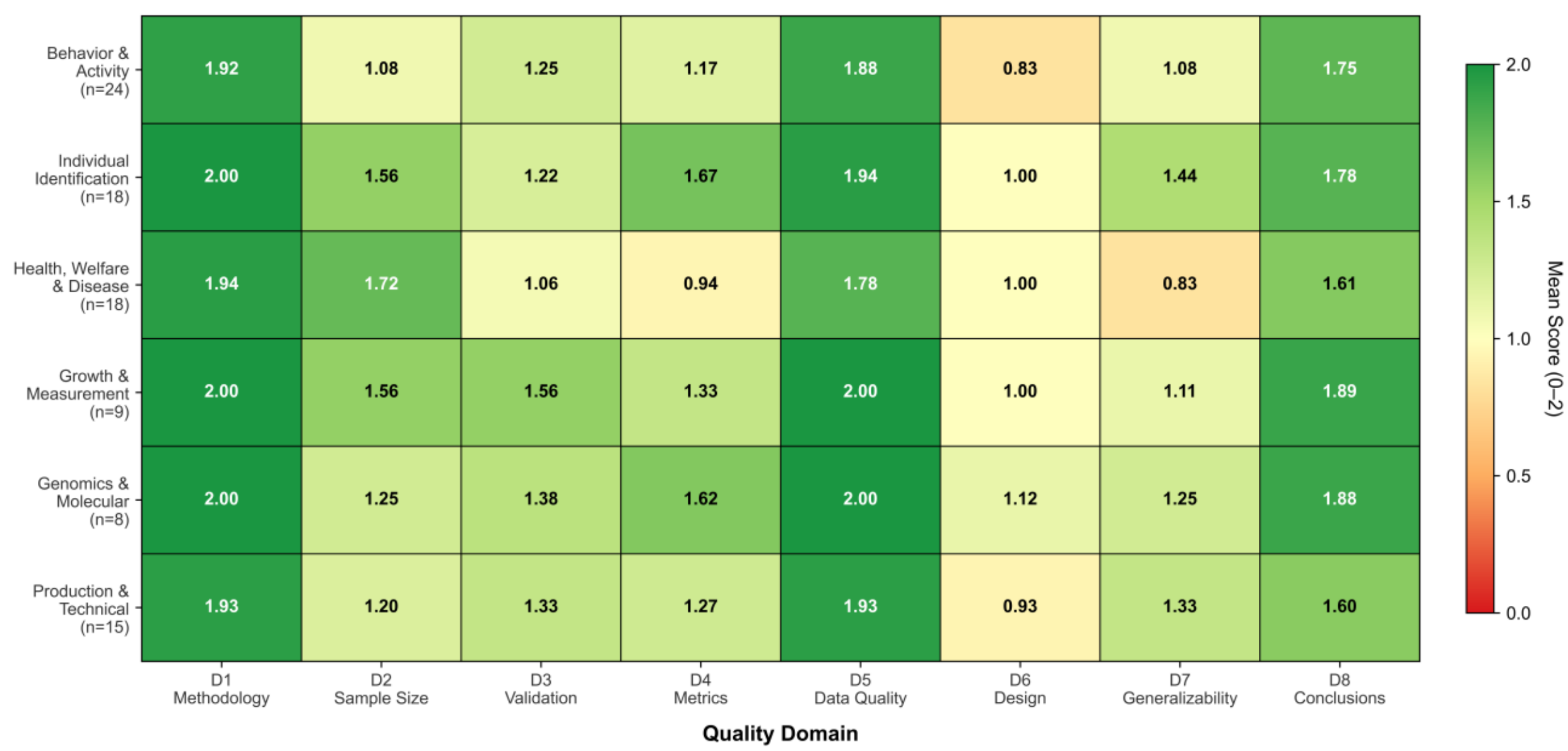

Figure S5. Study Quality by Validation Strategy – D3 Score (Absolute & Normalized).

Dual panel: (a) absolute counts and (b) normalized percentages of quality tiers by D3 validation strategy score. D3=0 (No separation / proof-of-concept only, n=3): all 3 studies are Low quality (100%). D3=1 (K-fold CV or internal test set, n=62): predominantly Moderate quality (54/62, 87.1%); 6 High (9.7%), 2 Low (3.2%). D3=2 (Independent hold-out, cross-farm, or field deployment, n=27): predominantly High quality (21/27, 77.8%); 6 Moderate (22.2%), 0 Low. A strong monotonic relationship is observed between validation rigor and study quality tier. Note: Verified D3 counts (D3=0 n=3, D3=1 n=62, D3=2 n=27) differ from Part E of the original table (D3=0 n=5, D3=1 n=32, D3=2 n=55).

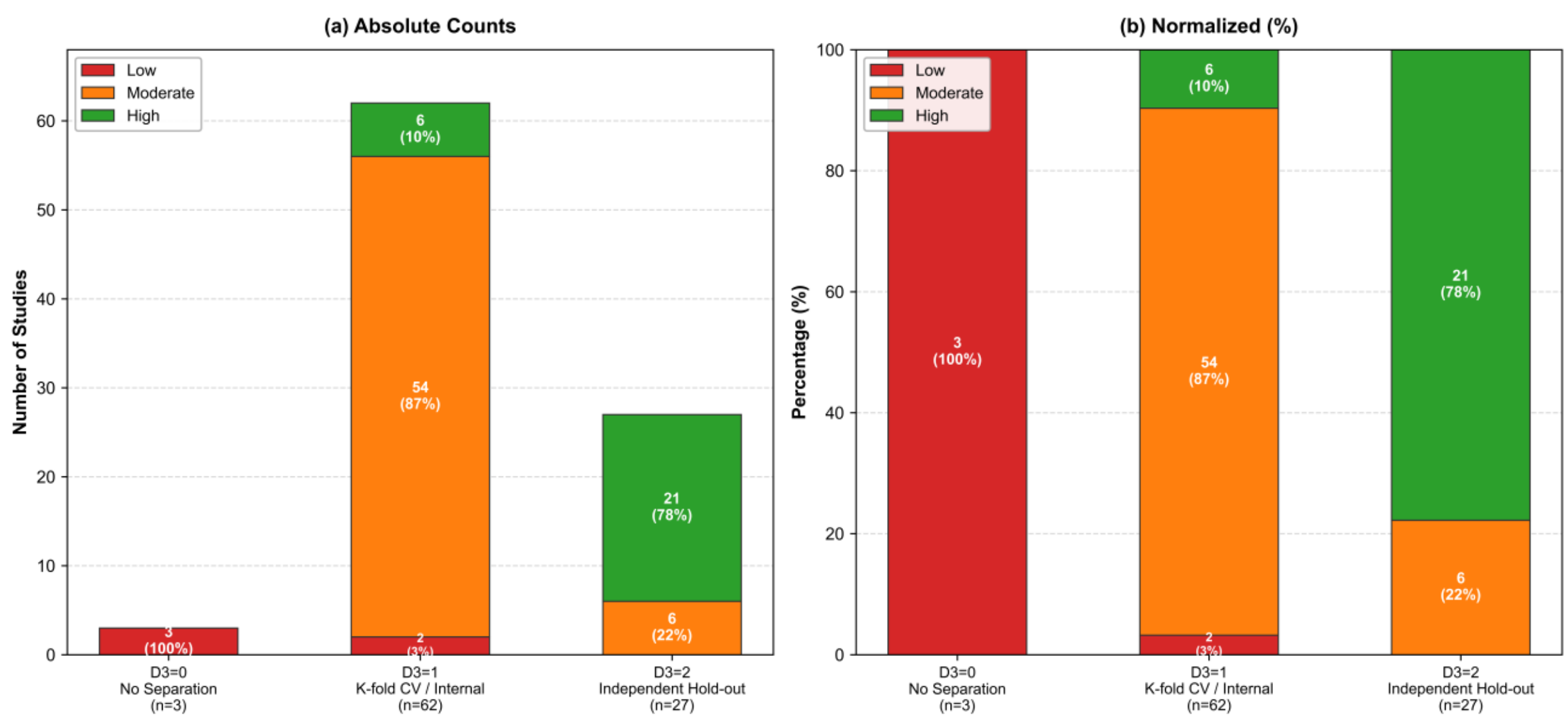

Figure S6. Distribution of Quality Scores by Research Group – Box Plot.

Box-and-whisker plots showing the spread of composite quality scores (0–16) within each research group. The box represents the interquartile range (IQR; Q1–Q3), the horizontal line inside the box is the median, and whiskers extend to 1.5×IQR. Points beyond whiskers are individual outliers. Growth & Measurement has the tightest distribution (scores 12–14, high consistency). Behavior & Activity shows the widest spread (scores 4–15), including the most outliers. Health, Welfare & Disease has the lowest median (≈12) and a long lower tail reaching score 4. Dashed green line = High quality threshold (13 pts); dashed red line = Low/Moderate boundary (7.5 pts). Based on verified study-level data; n=92.

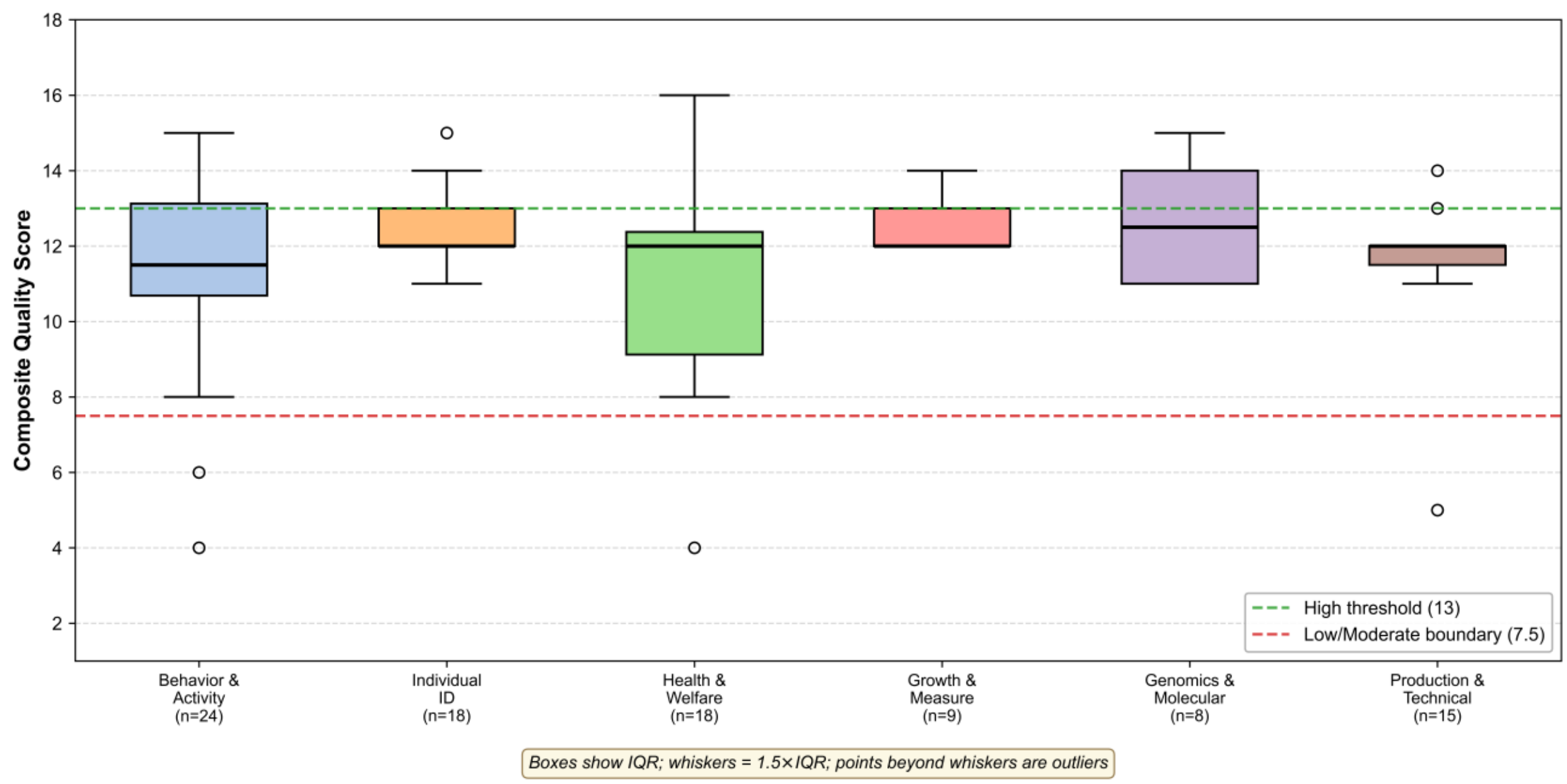

Figure S7: Geographic Distribution – Studies by Country

China dominates with 40 studies (43.5%), far exceeding all other countries combined. Turkey and Australia follow with 7 studies each (7.6%), and Greece ranks fourth with 6 studies (6.5%). The remaining countries contribute 1–4 studies each, spanning Europe, the Americas, Asia, and Oceania. This concentration highlights the scale of Chinese livestock research programs.

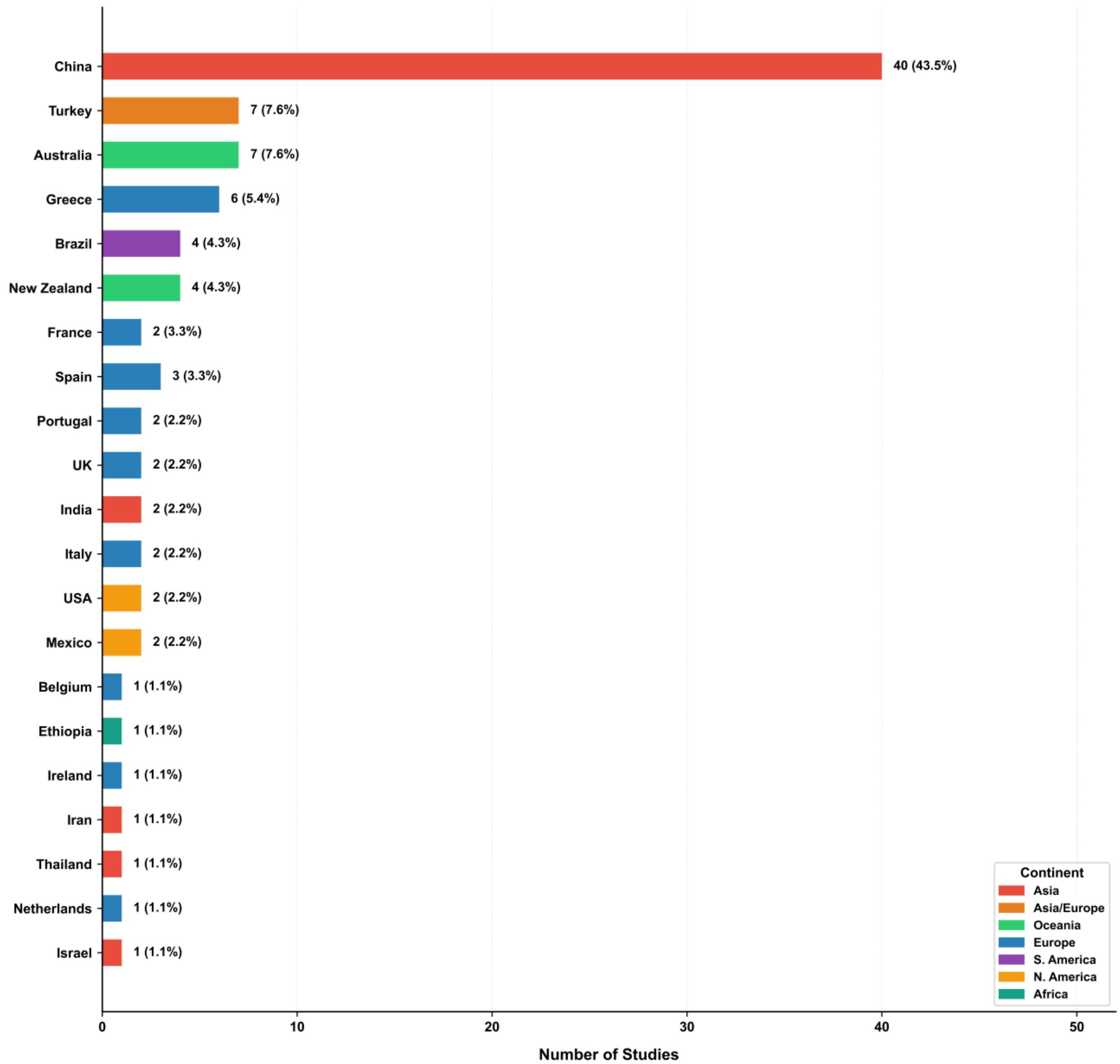

Figure S8: Geographic Distribution by Continent

Asia accounts for 56.5% of global research output (n=52), driven almost entirely by China. Europe contributes 21.7% (n=20), Oceania 12.0% (n=11). North and South America each contribute 4.3% (n=4), while Africa is represented only by Ethiopia (n=1), underscoring significant geographic disparities in livestock AI research.

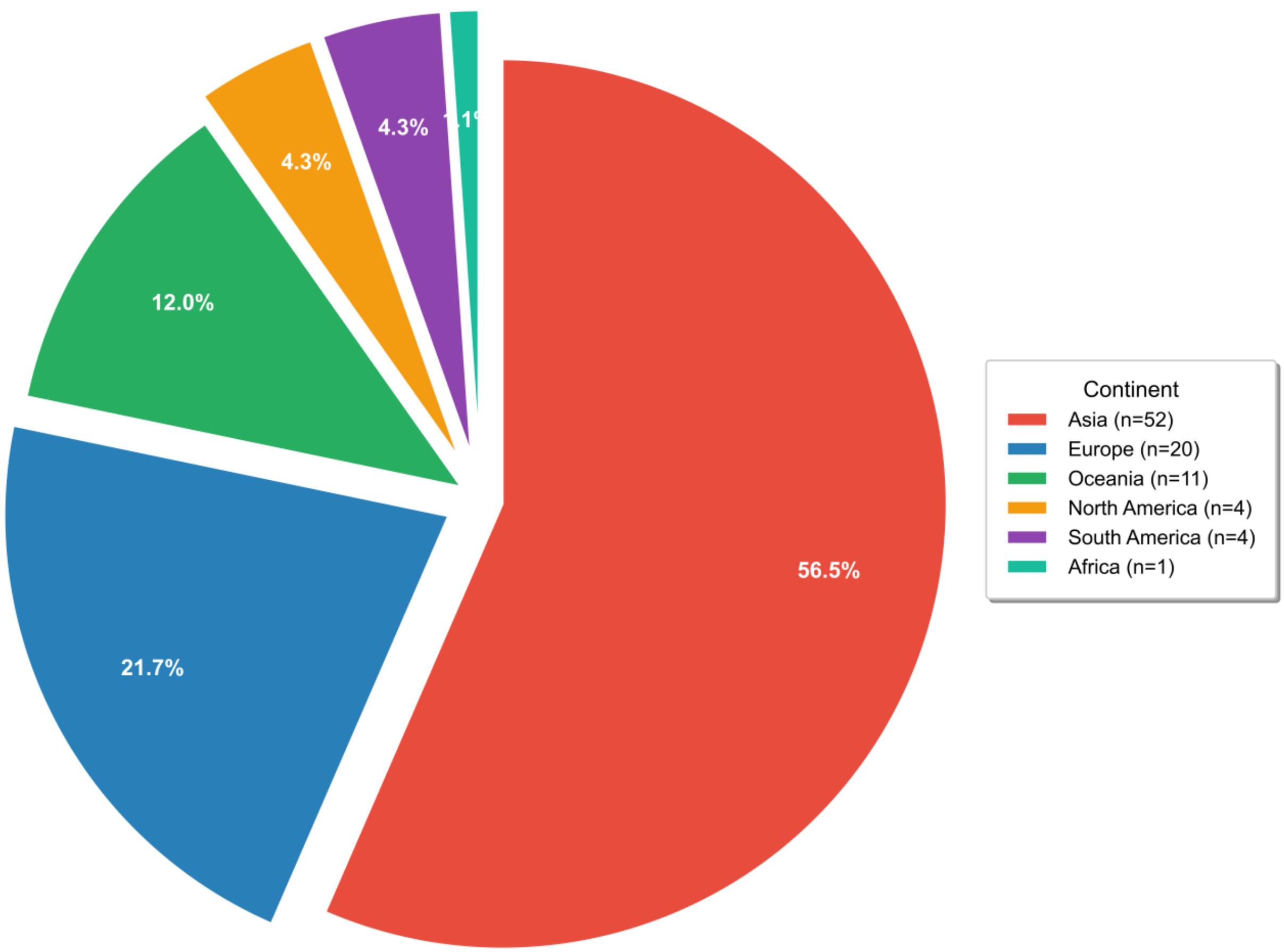

Figure S9: Performance Score Distribution by Research Group

Individual Identification achieves the tightest and highest distribution (IQR: ~97–99.5%). Health & Welfare shows the widest spread (range: ~76–99%), driven by diverse task types. Behavior & Activity spans from ~87% to 100%. Genomics & Molecular Biology maintains consistently high scores (>91%), while Growth & Measurement and Production & Technical groups show moderate median values around 93–96%.

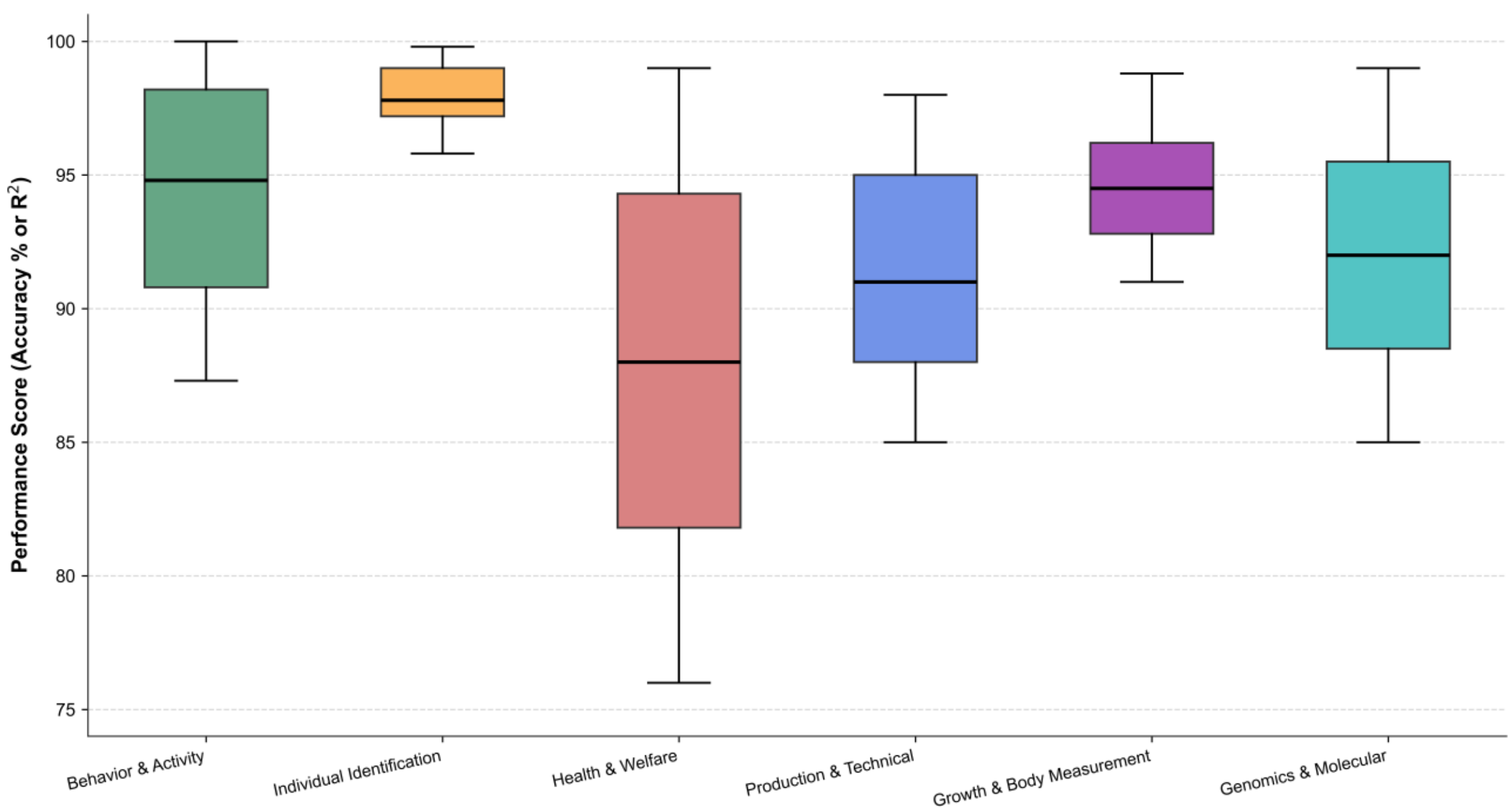

Figure S10: Distribution of Validation Strategies

Internal test set validation is used in approximately 70% of studies (n=25+). Cross-validation / K-fold accounts for ~16% (n=6). Field validation or deployment testing is rare at ~11% (n=4). Only one study used independent unseen animals (~3%), revealing a critical methodological gap between model development and real-world deployment readiness.

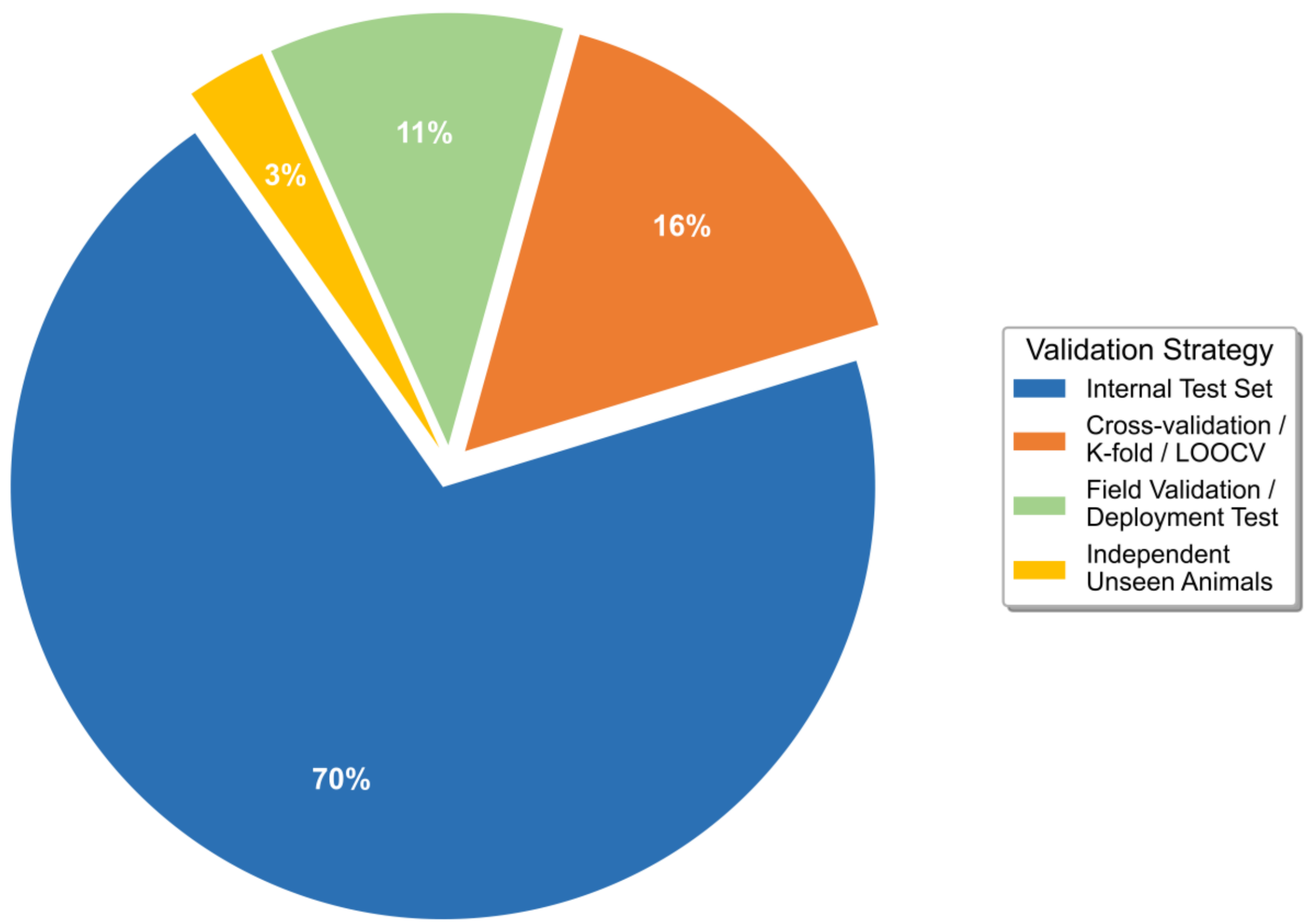

Figure S11: Species Distribution by Research Group

Sheep studies consistently outnumber goat studies across all six groups. The gap is largest in Behavior & Activity (Sheep: 18, Goat: 6) and Health & Welfare (Sheep: 12, Goat: 6). Individual Identification shows the most balanced ratio (Sheep: 10, Goat: 8), due to active goat facial recognition research. Genomics & Molecular Biology has the smallest numbers but both species are equally represented.

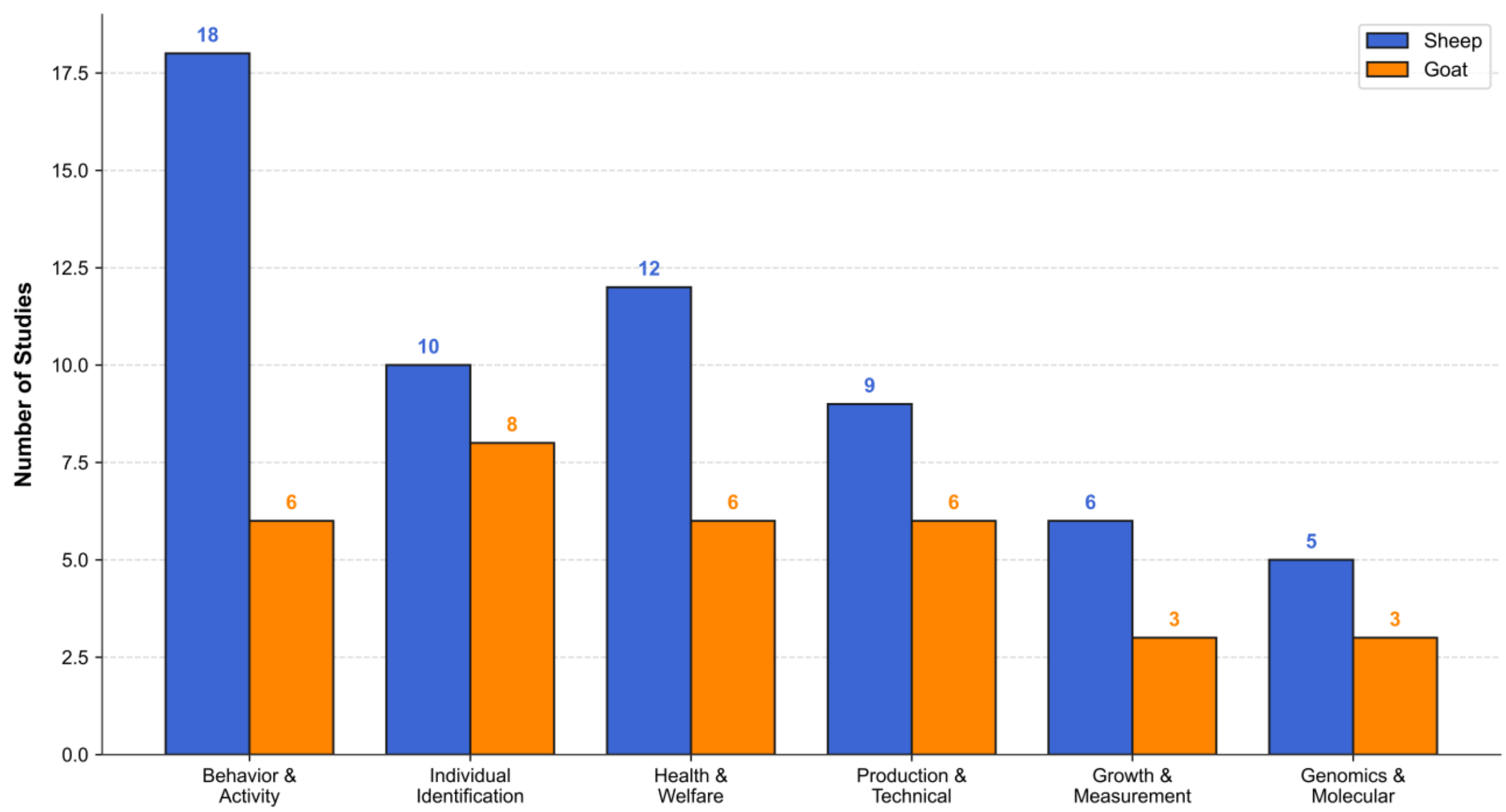

Figure S12: Top 10 Most Used AI / ML Technologies

CNN and deep learning rank first (n=20), used across behavior, identification, genomics, and production groups. YOLO-based models rank second (n=14) for real-time detection. Random Forest (n=12) and ANN/Neural Networks (n=11) serve diverse tasks. Tree-based ensembles total 7 uses. LSTM/RNN (n=5) is limited to time-series behavior data. Transformer-based models (n=4) are emerging.

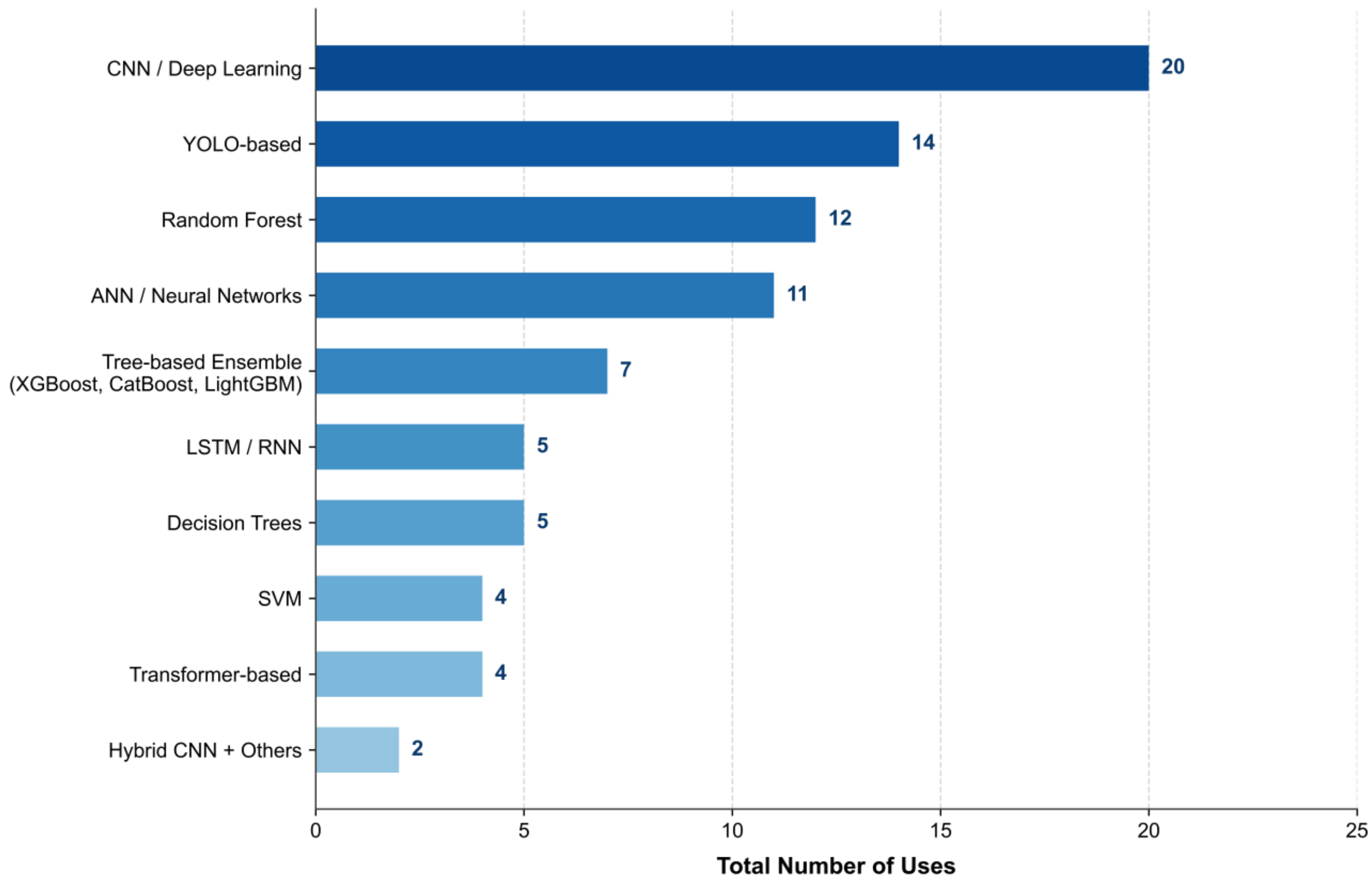

Figure S13: Top 10 Most Common Applications

Live weight prediction and image segmentation/detection lead jointly with 5 studies each. Mastitis detection, parasite/GIN resistance, and lambing detection follow with 4 each — addressing critical health and welfare priorities. Growth trait prediction (n=3), UAV/remote sensing, and food science applications (n=2 each) represent innovative emerging uses.

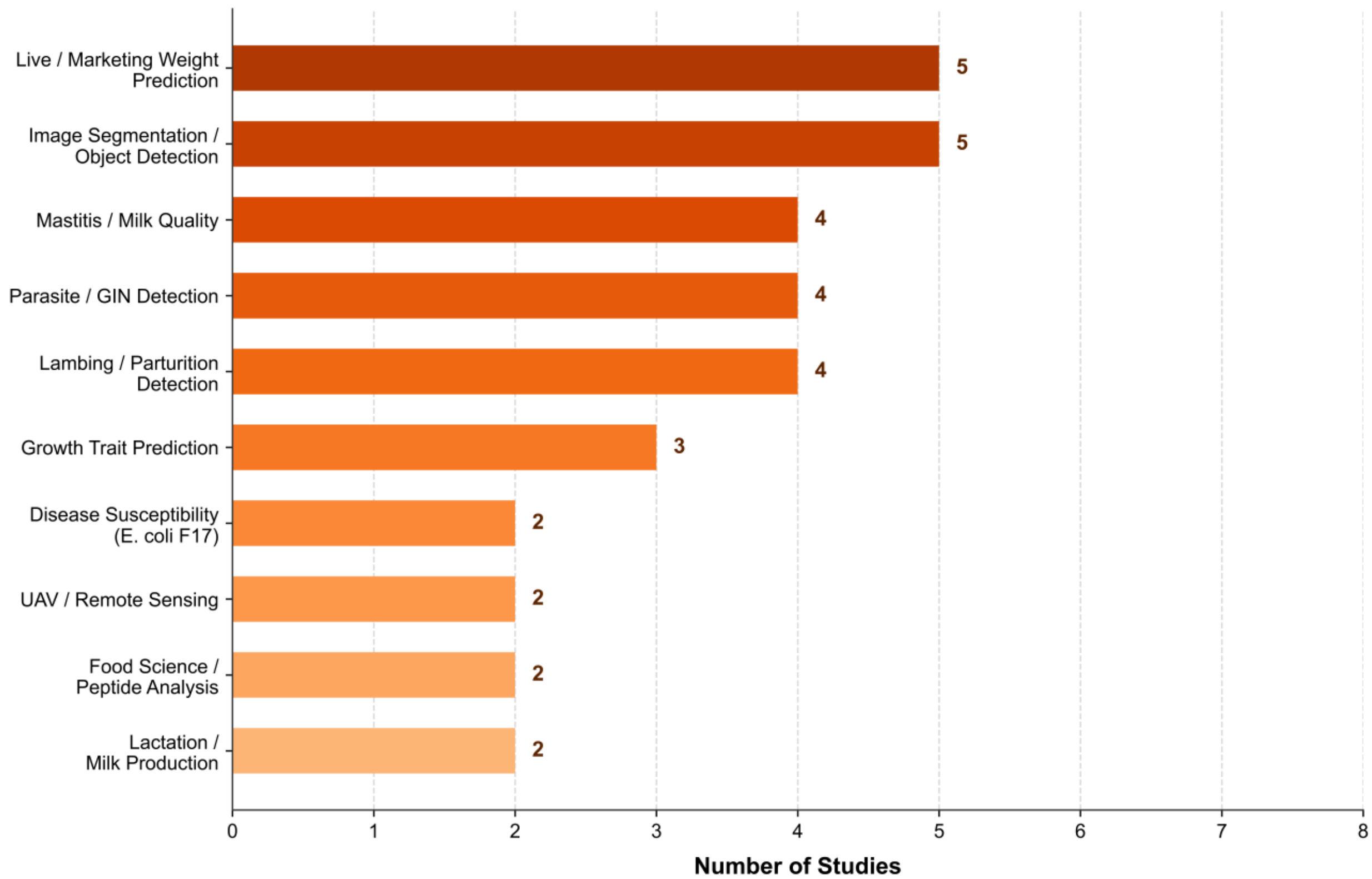

Figure S14: Yearly Publication Trends with Linear Trendline (2020–2025)

Publication output grew from 4 studies in 2020 to a peak of 26 in 2025, totaling 92 studies over the review period. The linear trendline ($R^2 = 0.821$) reveals a statistically significant growth of approximately 4.2 studies per year ($p < 0.05$), confirming sustained research interest. The year 2023 saw the largest single-year jump (+14 studies). The cumulative curve shows the field surpassed the 50% threshold during 2023, reaching 52.2% of total output by year's end.

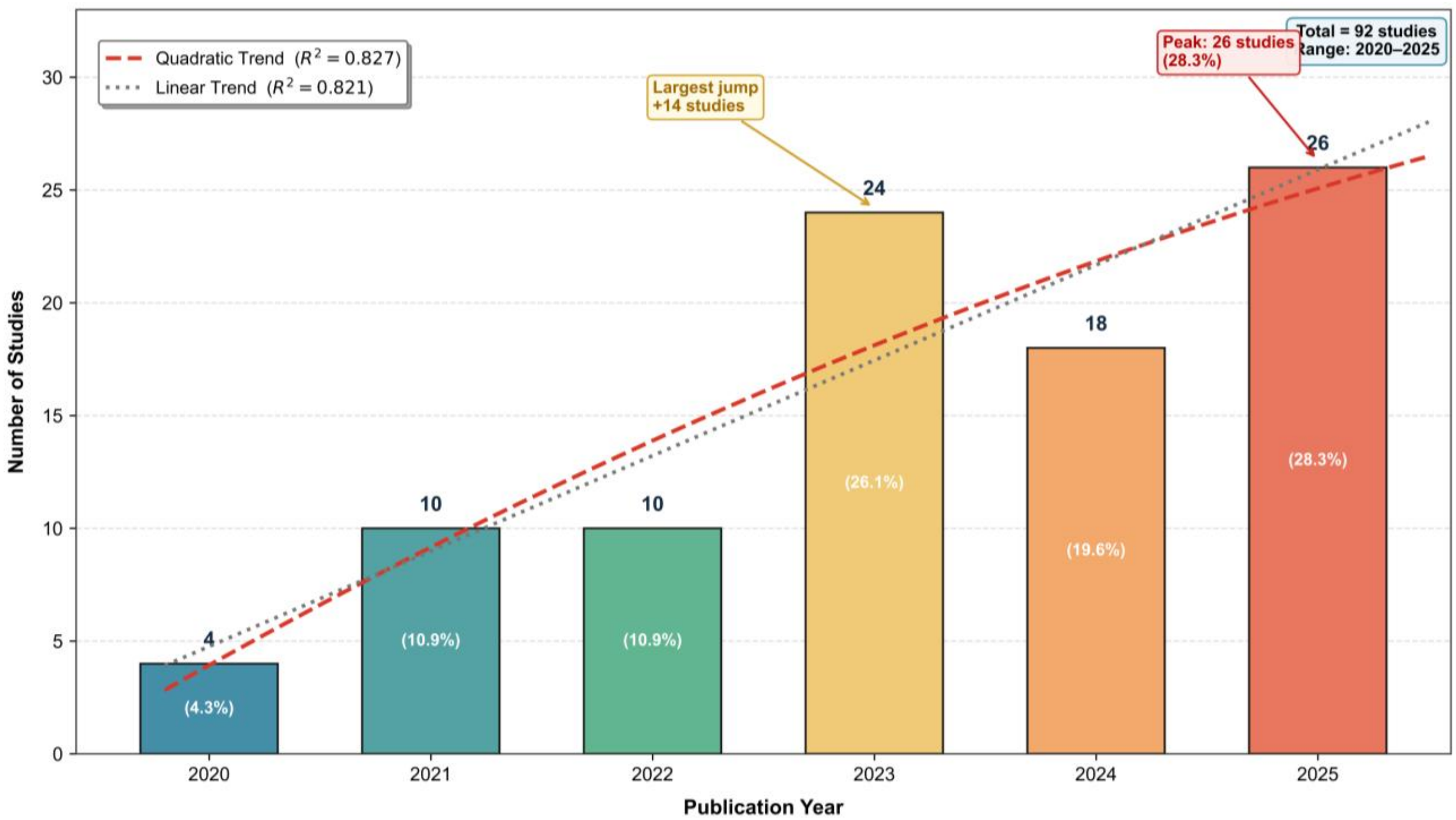

Figure S15: Types of Input Data Used in Studies

Camera and video data are the most common input modality (n=13). Facial images rank second (n=12), used primarily in Individual Identification. Accelerometer data (n=8) serves Behavior & Activity exclusively. Acoustic signals (n=2) are used for vocalisation analysis. Other modalities — IMU+GPS, electronic devices, retinal images — each appear once, indicating niche but novel strategies.

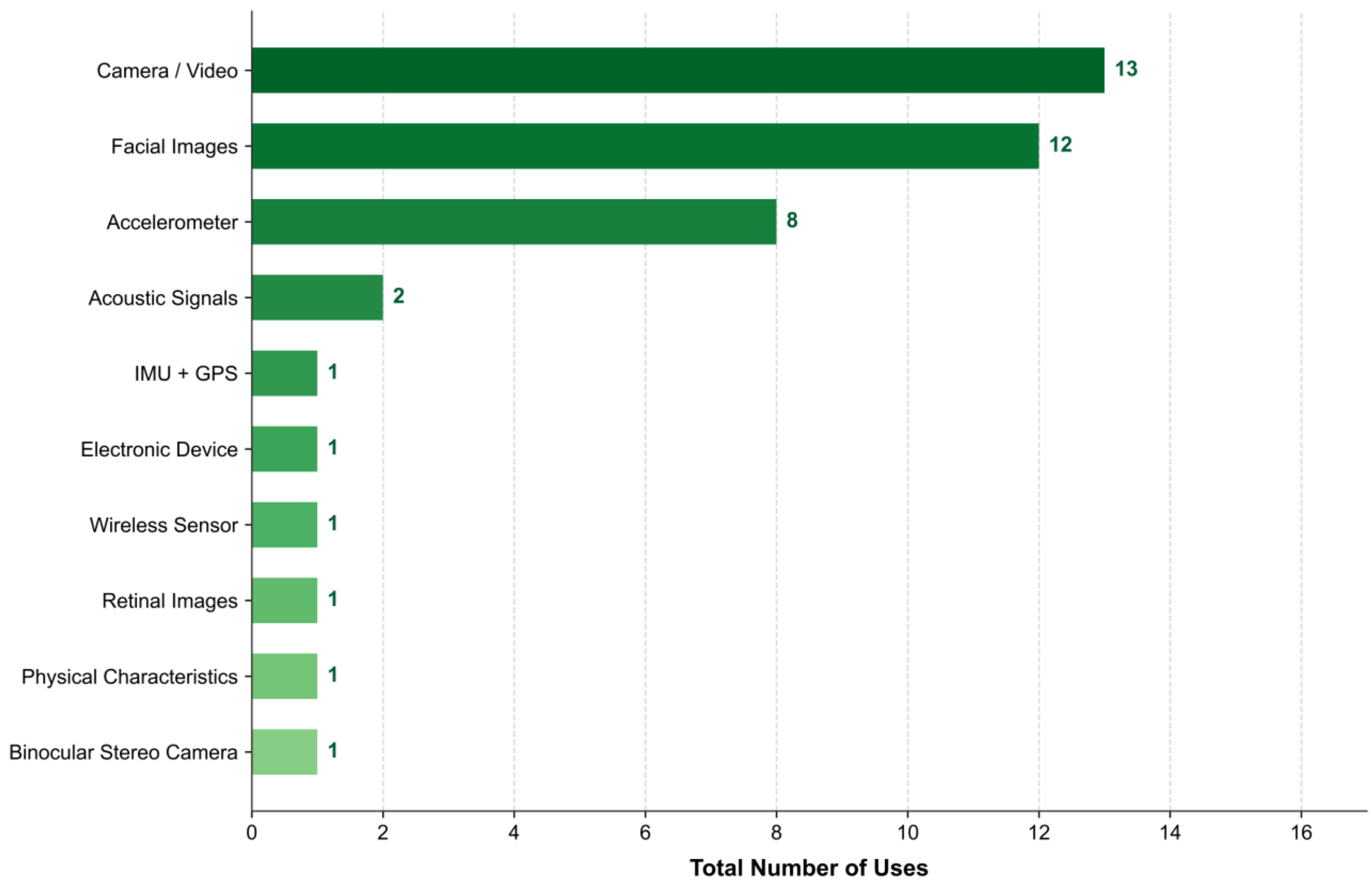

Figure S16: Technology Adoption Timeline (2020–2025)

CNN/DL shows consistent growth from 1 study in 2020 to 5 in 2025. YOLO-based models grew sharply from 2022, peaking in 2025 at 4 studies. Random Forest maintained stable usage throughout, peaking in 2023–2024. LSTM/RNN usage remained low (0–1 per year), reflecting its niche suitability for sequential sensor data. The trend indicates accelerating adoption of vision-based deep learning over classical ML.

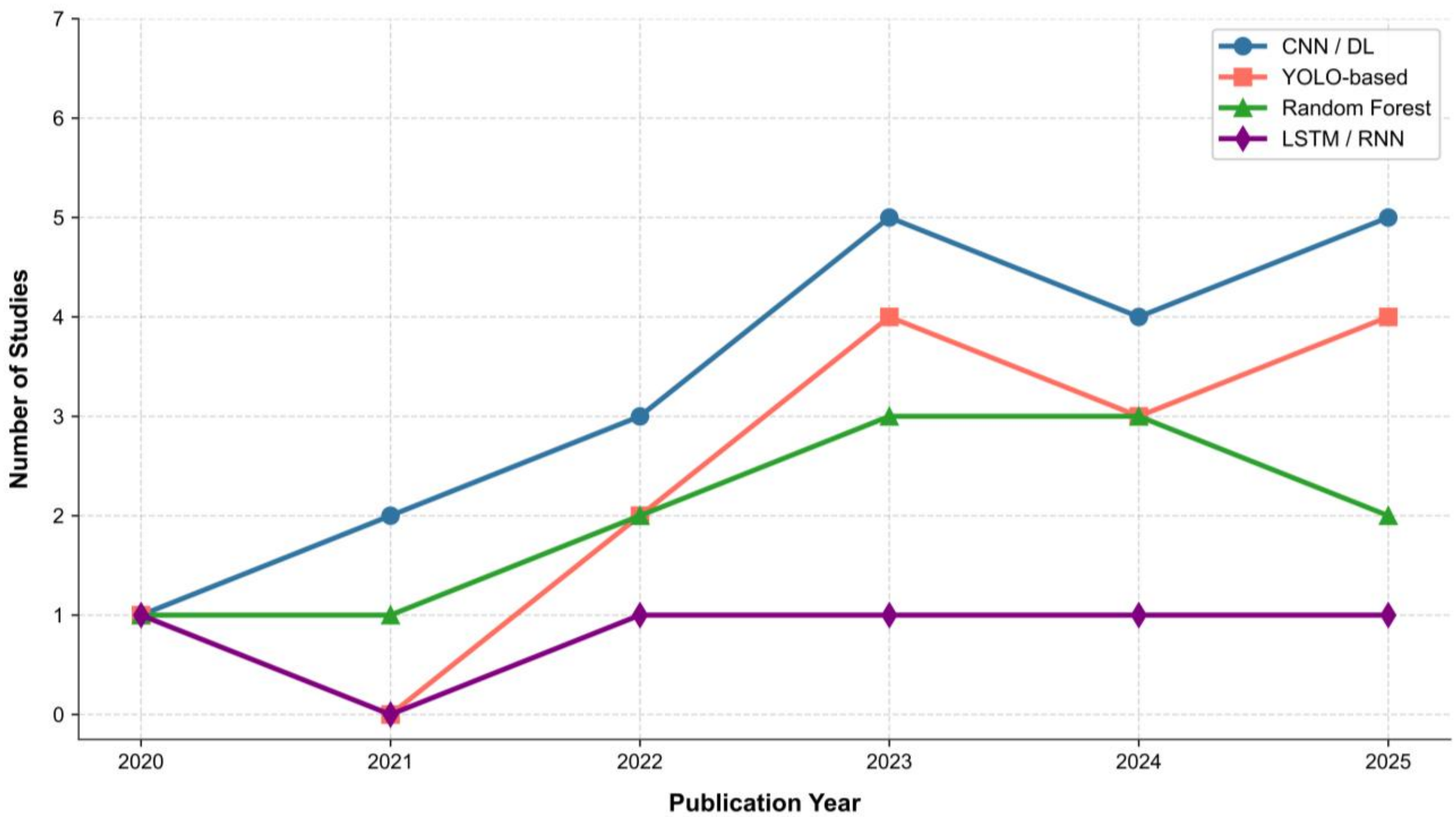

Figure S17: Strength Patterns in High-Quality Studies

Robust validation using cross-validation, unseen animals, or field tests is the most common strength (n=12, 34%). Lightweight, edge-ready architectures (n=7, 20%) and multi-model comparison/ensemble strategies (n=7, 20%) are equally common. High accuracy above 95% is achieved by 6 studies. XAI/interpretability via SHAP or CRP (n=4) represents a key research opportunity.

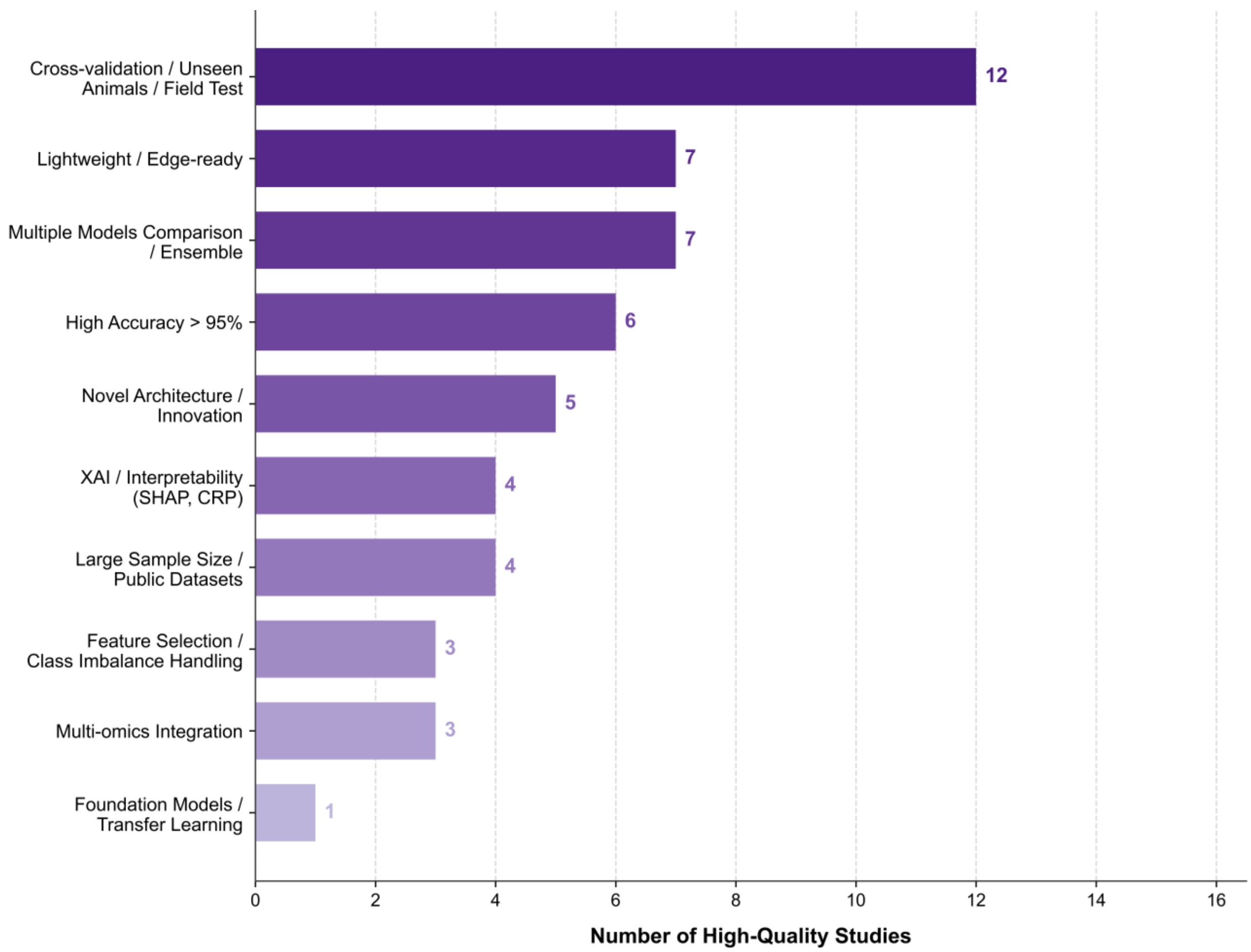

Figure S18: Top 10 Best-Performing Studies

Bati & Ser (2023) achieved a perfect 100% accuracy using BOF-CNN for behavioral recognition. Huang et al. (2023) attained 99.89% using DWT-GoatNet for goat identification. The Individual Identification group dominates the top 10 with 6 entries, reflecting the well-constrained nature of biometric recognition tasks. The 5-miRNA signature study by Farr et al. (2025) demonstrates molecular approaches can reach 99% clinical-grade accuracy.

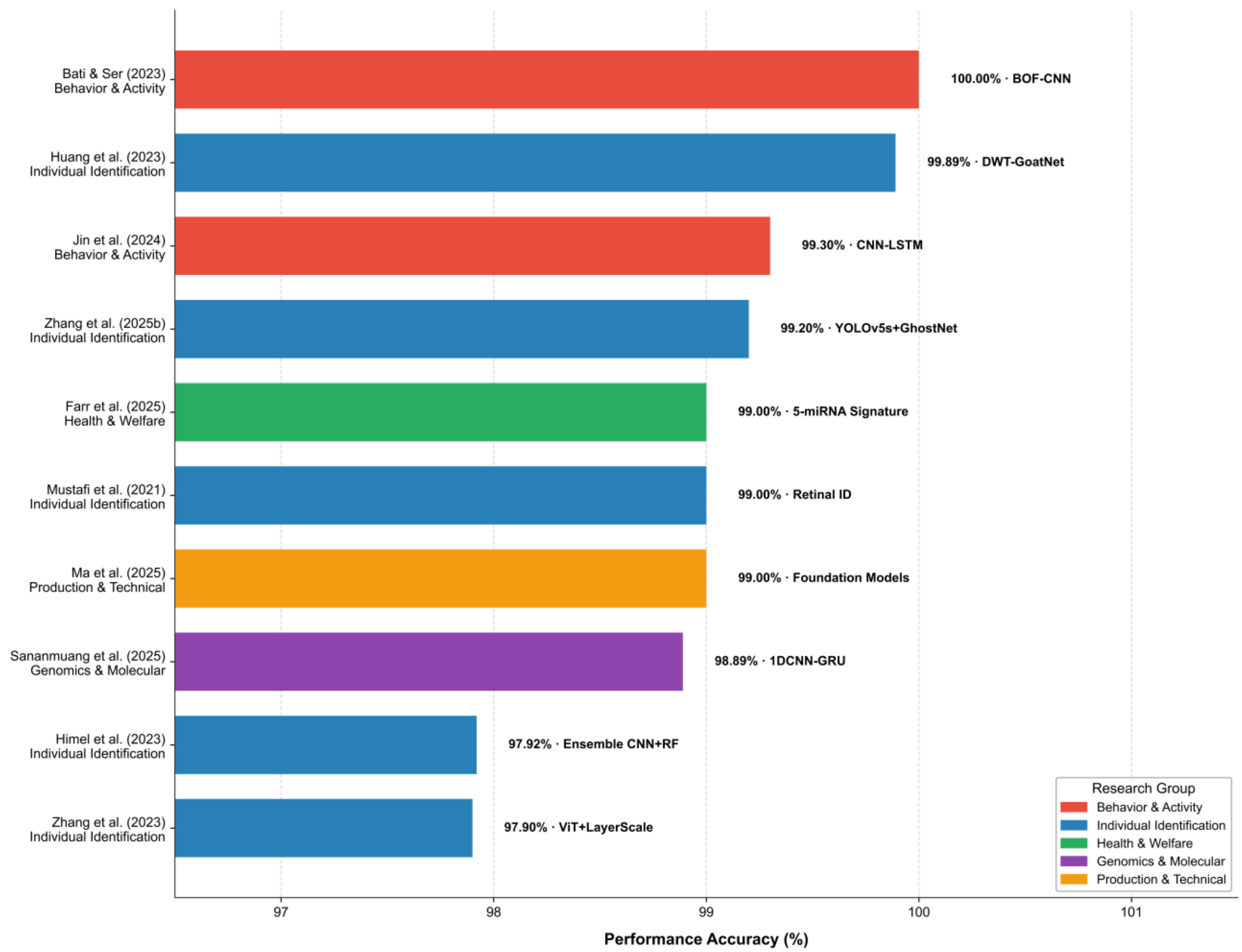

Figure S19: Country × Research Group Specialization Heatmap

China contributes overwhelmingly to Behavior & Activity (n=24). Greece focuses on Health & Welfare (n=4). Turkey specializes in Production & Technical (n=4). Australia distributes research across Behavior (n=3) and Health (n=3). The USA contributes solely to Health & Welfare (n=2). This pattern suggests national research programs are driven by domestic economic priorities in livestock production.

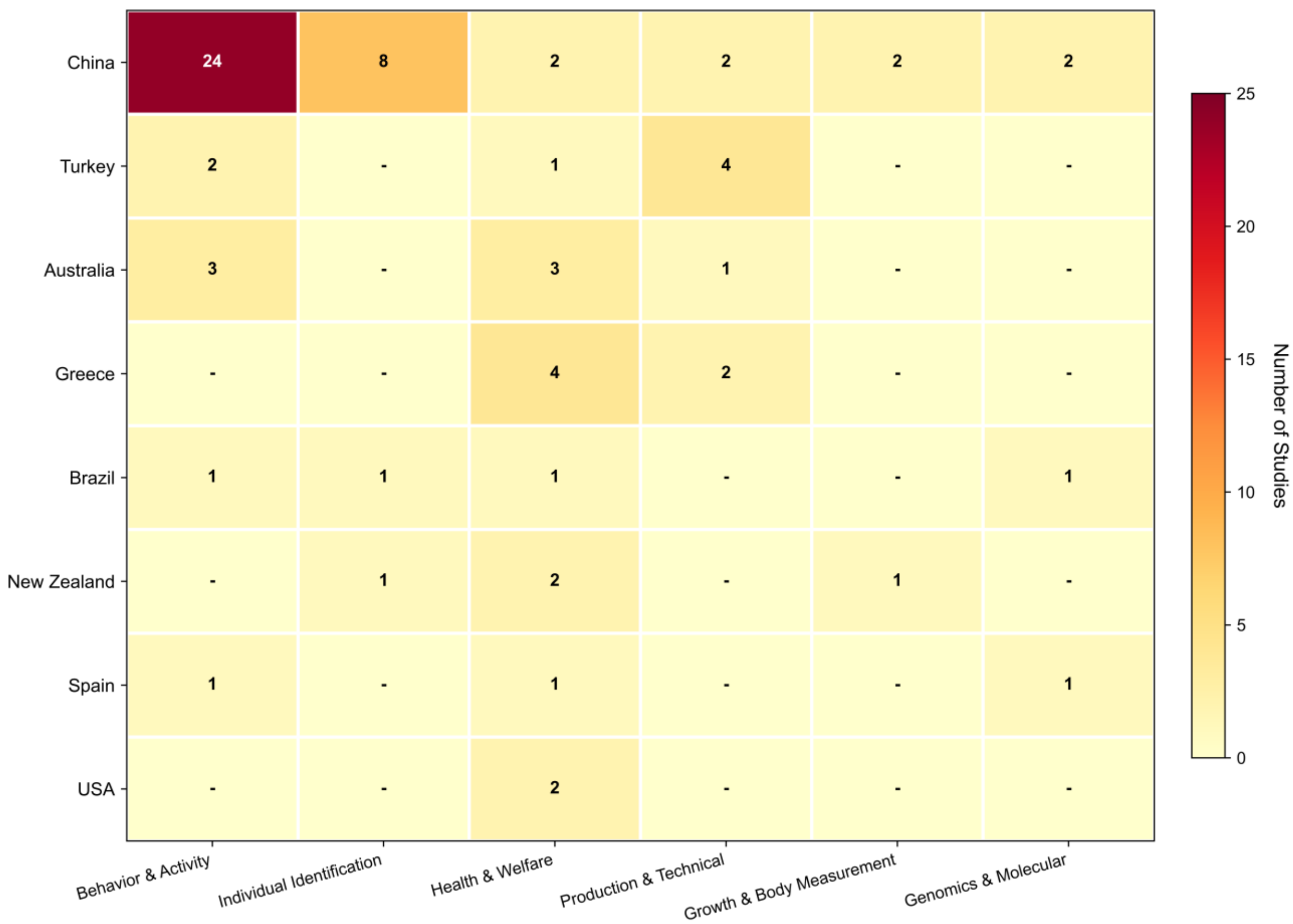

China dominates Behavior & Activity (n=24). Greece specializes in Health & Welfare (n=4). Turkey focuses on Production (n=4).

Supplementary References

1. Alemu, S. W., Bilton, T. P., Johnson, P. L., Perry, B. J., Henry, H., Dodds, K. G., ... & Rowe, S. J. (2025). Improving genomic prediction accuracy for methane emission and feed efficiency in sheep: Integrating rumen microbial PCA with host genomic variation using neural network GBLUP (NN-GBLUP). *Genetics Selection Evolution*, 57(1), 41.
2. Assefa, A., Tibebu, A., Bihon, A., & Yimana, M. (2021). Global ecological niche modelling of current and future distribution of peste des petits ruminants virus (PPRv) with an ensemble modelling algorithm. *Transboundary and Emerging Diseases*, 68(6), 3601–3610.
3. Ataç, F. E., Takma, Ç., Gevrekci, Y., & Altinçekiç, Ş. Ö. (2022). Prediction of marketing live weights in Hair goat kids using artificial neural network. *Kafkas Üniversitesi Veteriner Fakültesi Dergisi*, 28(6).
4. Bati, C. T., & Ser, G. (2023). SHEEPFEARNET: Sheep fear test behaviors classification approach from video data based on optical flow and convolutional neural networks. *Computers and Electronics in Agriculture*, 204, 107540.
5. Betancor-Sánchez, M., González-Cabrera, M., Morales-delaNuez, A., Hernández-Castellano, L. E., Argüello, A., & Castro, N. (2025). Enhancing immunoglobulin G goat colostrum determination using color-based techniques and data science. *Animals*, 15(1), 31. https://doi.org/10.3390/ani15010031
6. Billah, M., Wang, X., Yu, J., & Jiang, Y. (2022). Real-time goat face recognition using convolutional neural network. *Computers and Electronics in Agriculture*, 194, 106730.
7. Bonneau, M., Faillot, L., Troupe, W., & Riaboff, L. (2025). Hierarchical long short term memory recurrent neural network for goats behaviour prediction from accelerometer data. *Smart Agricultural Technology*, 11, 101045.
8. Bucki, M., Dhufaigh, K. N., O'Brien, C., Weatherley, A., Walshe, N., & McElligott, T. (2023). Comparison of ovine faecal Strongyle egg counts from an accredited laboratory and a rapid, on-site parasite diagnostic system utilising a smartphone app and machine learning. *Veterinary Parasitology*, 320, 109976.
9. Chen, W., Lv, X., Cao, X., Yuan, Z., Wang, S., Getachew, T., ... & Sun, W. (2023). Integration of the microbiome, metabolome and transcriptome reveals Escherichia coli F17 susceptibility of sheep. *Animals*, 13(6), 1050.
10. Chen, W., Lv, X., Zhang, W., Hu, T., Cao, X., Ren, Z., ... & Sun, W. (2022). Non-coding transcriptome provides novel insights into the Escherichia coli F17 susceptibility of sheep lamb. *Biology*, 11(3), 348.
11. de Souza, L. F., Costa, M. H., & Riet-Correa, B. (2023). Mobile app for targeted selective treatment of haemonchosis in sheep. *Veterinary Parasitology*, 316, 109902.
12. do Nascimento Amorim, M., dos Santos Costa, D., dos Santos Harada, É., da Silva, W. P., & Turco, S. H. N. (2025). Performance of electronic device and different visual observation intervals in assessing feeding behavior in sheep. *Computers and Electronics in Agriculture*, 231, 110053.
13. Emsen, E., Odevci, B. B., & Kutluca Korkmaz, M. (2025). Using machine learning to identify key predictors of maternal success in sheep for improved lamb survival. *Frontiers in Animal Science*, 6, 1543490.
14. Erduran, H., Esener, N., Keskin, İ., & Dağ, B. (2024). Machine learning-based early prediction of growth and morphological traits at yearling age in pure and hybrid goat offspring. *Tropical Animal Health and Production*, 56(8), 262.

15. Eroğlu, M., Turgut, A. O., Küçük, M., & Önen, M. F. (2025). Predicting weaning weight of Romanov lambs from biometric measurements before weaning age using machine learning algorithms. *Veterinary Medicine and Science*, 11(4), e70420. https://doi.org/10.1002/vms3.70420
16. Esener, N., & Kal, Y. (2025). Predicting weaning weight in Hair goat kids: A comparative analysis of tree-based machine learning approaches using environmental and herd data. *Animal Production Science*, 65(9), AN24154.
17. Farr, R. J., Cowled, C., Rodrigues, C., Rootes, C. L., Campbell, D. L., Lee, C., ... & Stoney, P. N. (2025). Circulating microRNA profiles are associated with acute pain and stress in castrated and tail docked lambs. *Veterinary and Animal Science*, 28, 100445.
18. Ferreira, J., Gonçalves, P., & Antunes, M. (2025). A two-stage approach for lambing detection. *Smart Agricultural Technology*, 101438.
19. Fogarty, E. S., Evans, C. A., Trotter, M. G., & Manning, J. K. (2023). Sensor-based detection of a Haemonchus contortus (Barber's pole worm) infection in sheep. *Smart Agricultural Technology*, 3, 100112.
20. Fogarty, E. S., Swain, D. L., Cronin, G. M., Moraes, L. E., Bailey, D. W., & Trotter, M. (2021). Developing a simulated online model that integrates GNSS, accelerometer and weather data to detect parturition events in grazing sheep: A machine learning approach. *Animals*, 11(2), 303.
21. Gu, Z., Zhang, H., He, Z., & Niu, K. (2023). A two-stage recognition method based on deep learning for sheep behavior. *Computers and Electronics in Agriculture*, 212, 108143.
22. Guevara, L., Castro-Espinoza, F., Fernandes, A. M., Benaouda, M., Muñoz-Benítez, A. L., del Razo-Rodríguez, O. E., & Partida de la Peña, J. A. (2023). Application of machine learning algorithms to describe the characteristics of dairy sheep lactation curves. *Animals*, 13(17), 2772.
23. Guo, X., Ma, C., Wang, C., Cui, X., Xu, G., Wang, R., ... & Zhang, L. (2025). A sheep behavior recognition approach based on improved FESS-YOLOv8n neural network. *Animals*, 15(6), 893.
24. Guo, Y., Li, S., Na, R., Guo, L., Huo, C., Zhu, L., ... & Zhang, W. (2024). Comparative transcriptome analysis of bovine, porcine, and sheep muscle using interpretable machine learning models. Animals, 14(20), 2947.
25. Gurule, S. C., Tobin, C. T., Bailey, D. W., & Gifford, J. A. H. (2021). Evaluation of the tri-axial accelerometer to identify and predict parturition-related activities of Debouillet ewes in an intensive setting. *Applied Animal Behaviour Science*, 237, 105296. https://doi.org/10.1016/j.applanim.2021.105296
26. Haldar, A., Pal, P., Ghosh, S., & Pan, S. (2023). Body weight prediction using recursive partitioning and regression trees (RPART) model in Indian Black Bengal goat breed: A machine learning approach. *Indian Journal of Animal Research*, 57(9), 1251-1257.
27. He, C., Qiao, Y., Mao, R., Li, M., & Wang, M. (2023). Enhanced LiteHRNet based sheep weight estimation using RGB-D images. *Computers and Electronics in Agriculture*, 206, 107667.
28. Himel, G. M. S., Islam, M. M., & Rahaman, M. (2024). Vision intelligence for smart sheep farming: applying ensemble learning to detect sheep breeds. Artificial Intelligence in Agriculture, 11, 1-12.

29. Hollevoet, A., De Waele, T., Peralta, D., Tuyttens, F., De Poorter, E., & Shahid, A. (2024). Goats on the move: evaluating machine learning models for goat activity analysis using accelerometer data. *Animals*, *14*(13), 1977.
30. Hou, Y., Han, M., Fan, W., Jia, X., Gong, Z., & Han, D. (2025). BiAF: Research on dynamic goat herd detection and tracking based on machine vision. *Scientific Reports*, 15(1), 4754. https://doi.org/10.1038/s41598-025-89231-6
31. Hu, S., Ingham, A., Schmoelzl, S., McNally, J., Little, B., Smith, D., ... & Bishop-Hurley, G. (2020). Inclusion of features derived from a mixture of time window sizes improved classification accuracy of machine learning algorithms for sheep grazing behaviours. *Computers and Electronics in Agriculture*, 179, 105857.
32. Huang, L., Qian, B., Guan, F., Hou, Z., & Zhang, Q. (2023). Goat face recognition model based on wavelet transform and convolutional neural networks. *Transactions of the Chinese Society for Agricultural Machinery*, 54(5), 278-287.
33. Jiang, M., Rao, Y., Zhang, J., & Shen, Y. (2020). Automatic behavior recognition of group-housed goats using deep learning. *Computers and Electronics in Agriculture*, 177, 105706.
34. Jin, Z., Shu, H., Hu, T., Jiang, C., Yan, R., Qi, J., ... & Liu, G. (2024). Behavior classification and spatiotemporal analysis of grazing sheep using deep learning. *Computers and Electronics in Agriculture*, 220, 108894.
35. Kaler, J., Mitsch, J., Vázquez-Diosdado, J. A., Bollard, N., Dottorini, T., & Ellis, K. A. (2020). Automated detection of lameness in sheep using machine learning approaches: Novel insights into behavioural differences among lame and non-lame sheep. *Royal Society Open Science*, 7(1), 190824.
36. Karamoutsios, A., Oikonomou, E. D., Voidarou, C., Hatzizisis, L., Fotou, K., Nikolaou, K., ... & Skoufos, I. (2025). Innovations in proteomic technologies and artificial neural networks: Unlocking milk origin identification. *BioTech*, 14(2), 33. https://doi.org/10.3390/biotech14020033
37. Kiouvrekis, Y., Vasileiou, N. G., Katsarou, E. I., Lianou, D. T., Michael, C. K., Zikas, S., ... & Fthenakis, G. C. (2024). The use of machine learning to predict prevalence of subclinical mastitis in dairy sheep farms. *Animals*, 14(16), 2295.
38. Li, J., Yang, Y., Liu, G., Ning, Y., & Song, P. (2024b). Open-set sheep face recognition in multi-view based on li-sheepfacenet. *Agriculture*, 14(7), 1112.
39. Li, X., Xiang, Y., & Li, S. (2023). Combining convolutional and vision transformer structures for sheep face recognition. *Computers and Electronics in Agriculture*, 205, 107651.
40. Li, X., Zhang, Y., & Li, S. (2024a). Rethinking lightweight sheep face recognition via network latency-accuracy tradeoff. *Computers and Electronics in Agriculture*, 227, 109662.
41. Lianou, D. T., Kiouvrekis, Y., Michael, C. K., Vasileiou, N. G., Psomadakis, I., Politis, A. P., ... & Fthenakis, G. C. (2024). The use of explainable machine learning for the prediction of the quality of bulk-tank milk in sheep and goat farms. *Foods*, 13(24), 4015.
42. Louta, M., Karagiannis, P., Papanikolopoulou, V., Vouraki, S., Tsipis, E., Priskas, S., ... & Gelasakis, A. I. (2023). FarmDain, a decision support system for dairy sheep and goat production. *Animals*, 13(9), 1495.
43. Lysitsas, M., Botsoglou, G., Dimitriadis, D., Termatzidou, S., Kazana, P., Tsoumakas, G., ... & Valiakos, G. (2024). Subclinical mastitis in Lacaune sheep: Etiologic agents, the effect

on milk characteristics, and an evaluation of infrared thermography and the YOLO algorithm as a preprocessing tool for advanced analysis. *Veterinary Sciences*, 11(12), 676.
44. Ma, Y., Ren, Q., Hettinga, K., & Fogliano, V. (2025). Leveraging foundation models and transfer learning for peptide transport prediction, molecular taste classification, and visual texture analysis. *Innovative Food Science & Emerging Technologies*, 104247.
45. Mao, A., Zhu, M., Huang, E., Yao, X., & Liu, K. (2023). A teacher-to-student information recovery method toward energy-efficient animal activity recognition at low sampling rates. *Computers and Electronics in Agriculture*, 213, 108242.
46. Martone, G., Bosco, A., Maturo, F., Santaniello, M., Ciuca, L., Cringoli, G., & Rinaldi, L. (2025). Surveillance and farmer perception of gastrointestinal nematode infections in sheep farms: A 10-year retrospective study. *Small Ruminant Research*, 107590. https://doi.org/10.1016/j.smallrumres.2025.107590
47. Méndez, D. A., Fajardo, B., Sanjuan, S., Calabuig, J. M., Arnau, R., Villagrá, A., & Estellés, F. (2025). Development of goat behaviour prediction with accelerometer data: A machine learning and pre-processing approach. *Computers and Electronics in Agriculture*, 237, 110701.
48. Mustafi, S., Ghosh, P., & Mandal, S. N. (2021). RetIS: Unique identification system of goats through retinal analysis. *Computers and Electronics in Agriculture*, 185, 106127.
49. Nóbrega, L., Gonçalves, P., Antunes, M., & Corujo, D. (2020). Assessing sheep behavior through low-power microcontrollers in smart agriculture scenarios. *Computers and Electronics in Agriculture*, 173, 105444.
50. Ntalampiras, S., & Pesando Gamacchio, G. (2025). Explainable classification of goat vocalizations using convolutional neural networks. *PLOS ONE*, 20(4), e0318543. https://doi.org/10.1371/journal.pone.0318543
51. Pang, Y., Yu, W., Zhang, Y., Xuan, C., & Wu, P. (2023). An attentional residual feature fusion mechanism for sheep face recognition. *Scientific Reports*, 13(1), 17128. https://doi.org/10.1038/s41598-023-44153-3
52. Paz-Kagan, T., Alexandroff, V., & Ungar, E. D. (2023). Detection of goat herding impact on vegetation cover change using multi-season, multi-herd tracking and satellite imagery. *Science of the Total Environment*, 895, 164830.
53. Price, E., Langford, J., Fawcett, T. W., Wilson, A. J., & Croft, D. P. (2022). Classifying the posture and activity of ewes and lambs using accelerometers and machine learning on a commercial flock. *Applied Animal Behaviour Science*, 251, 105630.
54. Pu, J., Yu, C., Chen, X., Zhang, Y., Yang, X., & Li, J. (2022). Research on Chengdu Ma goat recognition based on computer vision. *Animals*, 12(14), 1746.
55. Samperio, E., Lidón, I., Rebollar, R., Castejón-Limas, M., & Álvarez-Aparicio, C. (2021). Lambs' live weight estimation using 3D images. *Animal*, 15(5), 100212.
56. Sananmuang, T., Puthier, D., & Chokeshaiusaha, K. (2025). A hybrid 1DCNN-GRU deep learning framework for classifying caprine granulosa cell fertility potential using single-cell transcriptomics. *Veterinary World*, 18(7), 1922.
57. Sant'Ana, D. A., Pache, M. C. B., Martins, J., Astolfi, G., Soares, W. P., de Melo, S. L. N., ... & Pistori, H. (2022). Computer vision system for superpixel classification and segmentation of sheep. *Ecological Informatics*, 68, 101551. https://doi.org/10.1016/j.ecoinf.2022.101551
58. Sarwar, F., Griffin, A., Rehman, S. U., & Pasang, T. (2021). Detecting sheep in UAV images. *Computers and Electronics in Agriculture*, 187, 106219.

59. Ser, G., Bati, C. T., Aydoğdu, N., & Karaca, S. (2025). Decoding sheep actions: Deep learning algorithms for in-depth analysis of behavioral reactivity. *Measurement*, 118625. https://doi.org/10.1016/j.measurement.2025.118625
60. Siddique, A., Panda, S. S., Khan, S., Dargan, S. T., Lewis, S., Carter, I., ... & Terrill, T. H. (2024). Innovations in animal health: artificial intelligence-enhanced hematocrit analysis for rapid anemia detection in small ruminants. *Frontiers in Veterinary Science*, *11*, 1493403.
61. Sohi, R., Almasi, F., Nguyen, H., Carroll, A., Trompf, J., Weerasinghe, M., ... & Jois, M. (2022). Determination of ewe behaviour around lambing time and prediction of parturition 7 days prior to lambing by tri-axial accelerometer sensors in an extensive farming system. *Animal Production Science*, 62(17), 1729-1738.
62. Sun, C., Hu, J., Wang, Q., Zhu, C., Chen, L., & Shi, C. (2025a). YOLO-BCD: A lightweight multi-module fusion network for real-time sheep pose estimation. *Sensors*, 25(9), 2687. https://doi.org/10.3390/s25092687
63. Sun, L., Liu, G., Yang, H., Jiang, X., Liu, J., Wang, X., ... & Wang, Z. (2023). LAD-RCNN: A powerful tool for livestock face detection and normalization. *Animals*, 13(9), 1446. https://doi.org/10.3390/ani13091446
64. Sun, S., Sun, X., Wang, C., Yuan, W., & Wang, F. (2025b). Sheep peripartum behavior recognition method based on improved YOLOv8n-pose. *Transactions of the Chinese Society of Agricultural Engineering*, 41(12), 258–268. https://doi.org/10.11975/j.issn.1002-6819.202412223
65. Suparwito, H., Thomas, D. T., Wong, K. W., Xie, H., & Rai, S. (2021). The use of animal sensor data for predicting sheep metabolisable energy intake using machine learning. *Information Processing in Agriculture*, 8(4), 494–504. https://doi.org/10.1016/j.inpa.2020.12.002
66. Tang, L., Zhu, L., Basang, Z., Zhao, Y., Li, S., Kong, X., ... & Wang, L. (2025). Transcriptomic profiling of hypoxia-adaptive responses in Tibetan goat fibroblasts. *Animals*, 15(10), 1407.
67. Trieu, L. L., Bailey, D. W., Cao, H., Son, T. C., Scobie, D. R., Trotter, M. G., & Fowers, B. (2022). Potential of accelerometers and GPS tracking to remotely detect perennial ryegrass staggers in sheep. *Smart Agricultural Technology*, 2, 100040. https://doi.org/10.1016/j.atech.2022.100040
68. Turner, K. E., Thompson, A., Harris, I., Ferguson, M., & Sohel, F. (2023). Deep learning based classification of sheep behaviour from accelerometer data with imbalance. *Information Processing in Agriculture*, 10(3), 377-390.
69. Vayssade, J. A., & Bonneau, M. (2024). Puzzle: Taking livestock tracking to the next level. *Scientific Reports*, 14(1), 18348. https://doi.org/10.1038/s41598-024-69058-3
70. Vázquez-Martínez, I., Tırınk, C., Salazar-Cuytun, R., Mezo-Solis, J. A., Garcia Herrera, R. A., Orzuna-Orzuna, J. F., ... & Chay-Canul, A. J. (2023). Predicting body weight through biometric measurements in growing hair sheep using data mining and machine learning algorithms. *Tropical Animal Health and Production*, 55(5), 307.
71. Wang, K., Wu, P., Cui, H., Xuan, C., & Su, H. (2021). Identification and classification for sheep foraging behavior based on acoustic signal and deep learning. *Computers and Electronics in Agriculture*, 187, 106275. https://doi.org/10.1016/j.compag.2021.106275

72. Wang, L., Chen, C., Chen, F., Wang, N., Li, C., Zhang, H., ... & Liu, Y. (2024). UGTransformer: A sheep extraction model from remote sensing images for animal husbandry management. *IEEE Transactions on Geoscience and Remote Sensing*, 62, 1-14.
73. Wang, W., Chen, S., Qiao, L., Zhang, S., Liu, Q., Yang, K., ... & Liu, A. (2023a). Four markers useful for the distinction of intrauterine growth restriction in sheep. *Animals*, 13(21), 3305.
74. Wang, Y., Han, D., Wang, L., Guo, Y., & Du, H. (2023b). Contextualized small target detection network for small target goat face detection. *Animals*, 13(14), 2365. https://doi.org/10.3390/ani13142365
75. Wei, Y., Zhang, L., Yang, F., Jiang, X., Zhang, J., Zhu, L., ... & Zhang, L. (2025). Automatic measurement method of sheep body size based on 3D reconstruction and point cloud segmentation. *Computers and Electronics in Agriculture*, 239, 110978.
76. Xie, B., Jiao, W., Wen, C., Hou, S., Zhang, F., Liu, K., ... & Wang, Y. (2021). Feature detection method for hind leg segmentation of sheep carcass based on multi-scale dual attention U-Net. *Computers and Electronics in Agriculture*, 191, 106482.
77. Xie, Y., Chen, Z., Li, T., Yan, X., Jiang, H., & Pan, Z. (2025). Livestock 3D posture estimation method based on the attention mechanism for binocular stereo matching. *Transactions of the Chinese Society of Agricultural Engineering*, 41(3), 163–170.
78. Xu, Y., Nie, J., Cen, H., Wen, B., Liu, S., Li, J., ... & Cai, Q. (2023). Spatio-temporal-based identification of aggressive behavior in group sheep. *Animals*, *13*(16), 2636.
79. Yadav, C. S., Peixoto, A. A. T., Rufino, L. A. L., Silveira, A. B., & Alexandria, A. R. (2024). Intelligent classifier for identifying and managing sheep and goat faces using deep learning. *AgriEngineering*, 6(4), 3586-3601.
80. Yan, C., Gong, P., Zheng, W., Chen, X., & Guo, L. (2025). Research on sheep mounting behavior recognition in complex scenes based on an improved YOLOv11. *Scientia Agricultura Sinica*, 58(18), 3783–3798.
81. Yang, T., Zheng, W., Wang, X., Li, Y., Xiao, M., Wei, G., ... & Huang, A. (2024). A novel hydrophobic peptide FGMp11: Insights into antimicrobial properties, hydrophobic sites on Staphylococcus aureus and its application in infecting pasteurized milk. *Food Chemistry Advances*, 4, 100697. https://doi.org/10.1016/j.focha.2024.100697
82. Yuan, H., Liu, Z., Cai, Z., Zhang, Y., & Cheng, M. (2025). Sheep identification based on face features with the method and practical application analysis. *Smart Agricultural Technology*, 101362.
83. ZareBidaki, M., Allahyari, E., Zeinali, T., & Asgharzadeh, M. (2022). Occurrence and risk factors of brucellosis among domestic animals: An artificial neural network approach. *Tropical Animal Health and Production*, 54(1), 62.
84. Zhang, C., Xuan, C., Yu, W., Hao, M., & Liu, F. (2021). Grazing behavior of herding sheep based on three-axis acceleration sensor. *Transactions of the Chinese Society for Agricultural Machinery*, 52(7), 307–313.
85. Zhang, F., Zhao, X., Wang, S., Qiu, Y., Fu, S., & Zhang, Y. (2025a). Research on herd sheep facial recognition based on multi-dimensional feature information fusion technology in complex environment. *Frontiers in Veterinary Science*, 12, 1404564.
86. Zhang, L., Zhao, B., He, C., Yang, F., & Zhang, J. (2024a). Behaviour recognition of housed sheep based on spatio-temporal information. *Journal of Applied Animal Research*, 52(1), 1-13.

87. Zhang, X., Xuan, C., Ma, Y., Liu, H., & Xue, J. (2024b). Lightweight model-based sheep face recognition via face image recording channel. *Journal of Animal Science*, 102, skae066.
88. Zhang, X., Xuan, C., Ma, Y., & Su, H. (2023). A high-precision facial recognition method for small-tailed Han sheep based on an optimised Vision Transformer. *Animal*, 17(8), 100886.
89. Zhang, Y., Li, W., Xu, X., Xie, M., Tang, L., Zheng, P., ... & Liu, Y. (2025b). Machine learning driven multi-omics analysis of the genetic mechanisms behind the double-coat fleece formation in Hetian sheep. *Frontiers in Genetics*, 16, 1582244.
90. Zhang, Y., Yu, C., Liu, H., Chen, X., Lei, Y., Pang, T., & Zhang, J. (2022). An integrated goat head detection and automatic counting method based on deep learning. *Animals*, 12(14), 1810. https://doi.org/10.3390/ani12141810
91. Zhao, H., Mao, R., Li, M., Li, B., & Wang, M. (2023a). SheepInst: A high-performance instance segmentation of sheep images based on deep learning. *Animals*, 13(8), 1338.
92. Zhao, S., Bai, Z., Wang, S., & Gu, Y. (2023b). Research on automatic classification and detection of mutton multi-parts based on swin-transformer. *Foods*, 12(8), 1642.
